# Gut microbiota restoration with oral pooled fecal microbiotherapy after intensive chemotherapy: the phase 1b CIMON trial^∗^

**DOI:** 10.1182/bloodadvances.2024015571

**Published:** 2025-04-10

**Authors:** Florent Malard, Sylvain Thepot, Thomas Cluzeau, Martin Carré, Delphine Lebon, Pierre Bories, Ollivier Legrand, Marianne Schwarz, Michael Loschi, Mathieu Meunier, Magalie Joris, Cyrielle Gasc, Juliette Jouve, Benoît Levast, Emilie Plantamura, Emmanuel Prestat, Antoine Sabourin, Béatrice Gaugler, Joel Dore, Christian Récher, Mohamad Mohty

**Affiliations:** 1 Department of Clinical Hematology and Cellular Therapy, Saint-Antoine Hospital, Assistance Publique - Hôpitaux de Paris, Sorbonne University, INSERM Unité mixte de recherche 938, Centre de Recherche Saint-Antoine, Paris, France; 2 Clinical Hematology, Angers University Hospital, Angers, France; 3 Hematology Department, Côte D'Azur University, Nice Hospital, Nice, France; 4 Hematology Department, Grenoble Alpes University Hospital, La Tronche, France; 5 Hematology Department, Amiens University Hospital, Amiens, France; 6 Hematology Department, Toulouse University Hospital, Institut Universitaire du Cancer de Toulouse Oncopole, Toulouse III Paul Sabatier University, Toulouse, France; 7 MaaT Pharma, Lyon, France; 8 MetaGenoPolis, Institut national de recherche pour l'agriculture, l'alimentation et l'environnement, Paris-Saclay University, Jouy-en-Josas, France

## Abstract

Intensive induction chemotherapy (IC) combined with broad-spectrum antibiotics for acute myeloid leukemia (AML) leads to gut microbiota dysbiosis, promoting pathological conditions and an increased incidence of complications, possibly limiting eligibility to allogeneic hematopoietic cell transplantation (alloHCT). The purpose of this dose-ranging phase 1 study (CIMON) was to evaluate the first-in-man use of MaaT033, a pooled, allogeneic, lyophilized, and standardized fecal microbiotherapeutic product, formulated as a delayed-release capsule for oral administration. Primary objectives of the study were to evaluate the maximum tolerable dose of MaaT033 in 21 patients with AML having undergone IC and antibiotics. Secondary objectives were to assess MaaT033 safety, its efficacy in restoring the patients’ gut microbiome using shotgun sequencing to evaluate the recommended dose regimen, and patient compliance. MaaT033 was shown to be safe and effective for gut microbiota restoration in patients with AML receiving IC and antibiotics, with an excellent gut microbiota reconstruction based on diversity indices at the species level and restoration of microbial communities close to the composition of the drug product. The maximum tolerable dose of MaaT033 was not determined because the interim results suggested adequate efficacy as measured by engraftment at lower doses (3 capsules per day). Moreover, inflammatory markers (C-reactive protein, interleukin-6) decrease with treatment, whereas short-chain fatty acids increase over time. A randomized, placebo-controlled phase 2b trial, in recipients of alloHCT patients is ongoing. This trial was registered at www.clinicaltrials.gov as #NCT04150393.

## Key Points

• MaaT033 is a pooled, allogeneic, lyophilized, and standardized fecal microbiotherapy.
• MaaT033 is safe and effective in restoring gut microbiota after intensive chemotherapy.

## Visual Abstract

**Figure:**
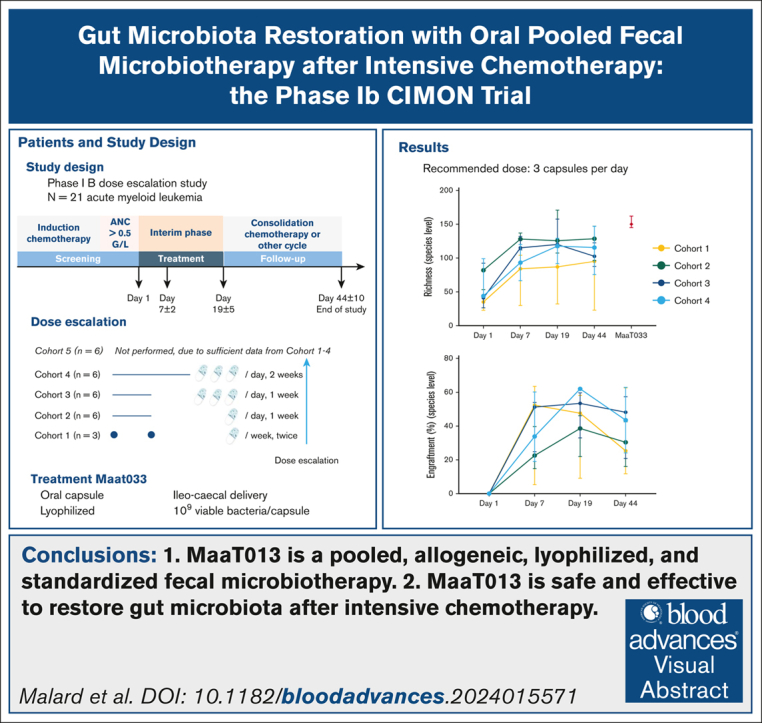

## Introduction

Host-microbiome symbiosis, in particular the interplay between the microbiome and the immune system, is associated with health and disease.^1^ Several lines of evidence support the notion that the gut microbiome regulates immune and clinical responses to cancer,^2^^,^^3^ in particular to immunotherapy such as immune checkpoint inhibitors or allogeneic hematopoietic cell transplantation (alloHCT).^2^, ^3^, ^4^, ^5^, ^6^, ^7^ Furthermore, the use of broad-spectrum antibiotics is associated with impaired outcomes after alloHCT or chimeric antigen receptor T cells.^8^, ^9^, ^10^ Therefore, therapeutic strategies developed to restore or preserve the gut microbiota composition are worth evaluating given that they could improve the outcome of patients undergoing immunochemotherapy.

Fecal microbiotherapy showed promising results, in particular in melanoma patients, where it circumvented primary and secondary immune checkpoint inhibitor resistance,^11^^,^^12^ but also for the treatment of graft-versus-host disease (GVHD).^13^ Nevertheless, fecal microbiotherapy essentially relies on stool bank availability, and the development of standardized microbiome-based therapeutics is essential to improve accessibility. Furthermore, fecal microbiotherapy usually requires in-patient administration, which may additionally contribute to reducing accessibility. With this background, we developed MaaT033, an oral, delayed-release size-0 capsule containing lyophilized pooled, full-ecosystem fecal microbiota that is readily available and allows outpatient delivery.

We decided to investigate this fecal microbiotherapy in patients with acute myeloid leukemia (AML) after intensive induction chemotherapy (IC). During IC, most patients receive broad-spectrum antibiotics, which leads to a dramatically altered gut microbiota.^14^^,^^15^ This results in a disruption of beneficial bacteria and a rise in dominance of some pathogens, pathobionts, and multidrug-resistant bacteria.^15^, ^16^, ^17^, ^18^, ^19^ Furthermore, these microbial shifts persist after the end of the IC.^20^^,^^21^ Loss of microbial diversity over the course of IC has clinical consequences with higher infection rates within the 90 days after IC neutrophil recovery.^16^ Therefore, the patient with post-IC AML is an optimal setting in which to evaluate fecal microbiotherapy safety and efficacy. This is particularly relevant because many patients with AML undergo alloHCT weeks to months after IC, and reduced gut microbiota diversity at the time of alloHCT predicts a higher risk of death after transplantation.^6^

This dose-ranging phase 1 study (CIMON) evaluated the first-in-man use of oral fecal microbiotherapy MaaT033 and aimed to demonstrate its safety and efficacy in restoring gut microbiota diversity in patients with AML treated with IC (ClinicalTrials.gov identifier: NCT04150393).

## Methods

### CIMON study design

The CIMON study was a phase 1, open-label, single-arm, multicenter, prospective, interventional trial to evaluate the first-in-man use of the pooled allogeneic oral microbiotherapy MaaT033 in patients with diagnosed AML and having undergone IC (see supplemental Table 1 for complete list of criteria). The primary objective of the study was to evaluate the maximum tolerable dose of MaaT033 after IC and antibiotic therapy given during aplasia. The secondary objectives were to evaluate the overall safety of MaaT033, to evaluate the product activity (evaluation of the microbiota modification and MaaT013 engraftment) to identify the recommended phase 2 dose, and to evaluate patient compliance. Dose-limiting toxicity (DLT) was defined as any related adverse event (AE) grade >3 based on Common Terminology Criteria for Adverse Events v5.0 criteria for blood and lymphatic system disorders (DLT when grade 4 or 5) and immune system disorders (DLT when grade 4 or 5) and any related AE grade ≥3 for all other system organ.

**Figure 1.**
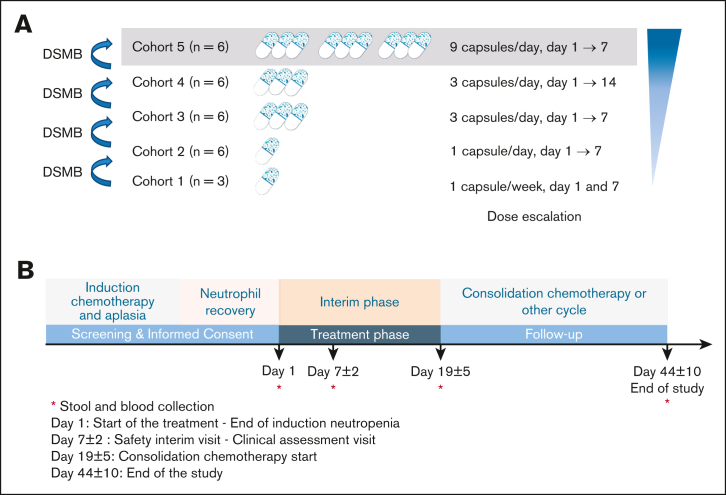
**Dose ranging and study flowcharts.** (A) Details of dose regimen per cohort. Of note, no patient was treated in cohort 5. (B) Study flowchart. The dose-escalation process was monitored by the data and safety monitoring board. Visit 1: treatment start (D1) at the end of neutropenia. Feces and blood collection. Visit 2: safety interim visit (D7 ± 2), clinical assessment visit. Feces and blood collection. Visit 3: start of consolidation or other cycle of chemotherapy (D19 ± 5), clinical follow-up. Feces and blood collection. Visit 4: end of study (D44 ± 10) corresponding to the end of chemotherapy. Feces and blood collection. The recommended timing for MaaT033 administration was 1 capsule before breakfast (for cohorts 1 and 2), 3 capsules before breakfast (for cohorts 3 and 4), and 3 capsules before breakfast, 3 before lunch, and 3 before dinner (for cohort 5).

The treatment group was planned to comprise 27 patients in total, distributed into 5 cohorts. The dose escalation was performed in a step-up dosing design from 2 capsules 7 days apart for cohort 1 to 9 capsules per day for 7 days for cohort 5 (Figure 1A). Dose-escalation rules regarding MaaT033 production are provided in the supplemental Methods. MaaT033 was started within 2 days after the documented end of neutropenia (ie, absolute neutrophil count ≥0.5 × 10^9^ cells per liter). Feces and blood were collected before the start of MaaT033 treatment on day 1 (D1), day 7 (D7) (D7 ± 2 days), and day 19 (D19 ± 5 days), before the start of a new chemotherapy cycle and shotgun metagenomic sequencing was conducted. A list of parameters and methods is provided in the supplemental Methods. The end of study was planned at day 44 (D44) (D44 ± 10 days), at the end of a chemotherapy cycle (Figure 1B for study flowchart). In addition, centers were contacted in November 2023 to collect long-term follow-up data, after D44, regarding survival or death, including date of relapse, date and cause of death, alloHCT (and date) or not, and the occurrence or not of acute GVHD (aGVHD) and chronic GVHD after alloHCT.

### Statistical analyses

For most visits and parameters, the normality distribution assumption was rejected by the Shapiro-Wilk test. Given the limited sample size, subsequent to the phase 1 study design not allowing the evaluation of the statistical significance, we only report descriptive results, except for the assessment of engraftment of MaaT033 at each visit by richness measured at D1 (at species level), whose statistical significance was performed using the Pearson correlation test. Overall survival was estimated by the Kaplan-Meier method.

## Results

### Patient characteristics

A total of 22 patients with newly diagnosed AML were screened in 6 different centers, 21 were treated with MaaT033, and 20 were considered as the per-protocol population (see supplemental Figure 2 for CONSORT diagram). Cohort 5 (initially planned in the study protocol) was not enrolled owing to the product activity (colonization of the gastrointestinal [GI] tract by different bacterial species and intermediate product microbial engraftment results) with identification of the minimum active dose. Therefore, the Data Safety and Monitoring Board (DSMB) (the DSMB charter and study protocol are provided in the Supplementary data) recommended not to enroll cohort 5 and to terminate the study early. The baseline characteristics of all patients are presented in Table 1 and for each patient in supplemental Table 3. Between the start of IC and first administration of MaaT033, all but 2 patients received broad-spectrum antibiotic treatments (piperacillin-tazobactam and/or carbapenem and/or third-generation cephalosporins) for either febrile neutropenia or documented infection. None of the antibiotics received during IC were related to an AE. Chemotherapies and antibiotics received between the start of IC and end of study are listed per patient in supplemental Tables 4 and 5.

**Table 1.** **Baseline demographics and clinical characteristics of patients in the CIMON study**

	Treated patients (N = 21)
**Sex, n (%)**	
Male	16 (76)
Female	5 (24)
**Age at inclusion, y**	
Median (range)	62 (22-77)
**Body mass index at inclusion, kg/m^2^**	
Median (range)	23.9 (16.7-33.1)
**Leukemia risk category (ELN 2022), n (%)**	
Favorable	7 (33)
Intermediate	6 (29)
Adverse	8 (38)
**Karyotype, n (%)**	
Normal	10 (48)
Abnormal	11 (52)
**Induction chemotherapy, n (%)**	
Cytarabine + anthracycline	20 (95)
Azacitidine + venetoclax	1 (5)
**Consolidation chemotherapy, n (%)**	
Cytarabine	14 (67)
Cytarabine + anthracycline	3 (15)
Daunorubicin	1 (5)
Azacitidine + venetoclax	1 (5)
None	1 (5)
Unknown (premature discontinuation)	1 (5)
**Patients who received antibiotics**∗**during IC, n (%)**	
Yes	19 (90)
No	2 (10)
**Patients who received antibiotics during consolidation chemotherapy, n (%)**	
Therapeutic antibiotics	10 (48)
Prophylactic antibiotics	3 (14)
No systemic antibiotics	7 (33)
Unknown (premature discontinuation)	1 (5)

ELN, European Leukemia Network.

∗ Piperacillin-tazobactam and/or carbapenem and/or third-generation cephalosporins.

### Administration of MaaT033 is feasible and safe

We first evaluated the maximum tolerable dose as the primary end point of the study. Patient compliance was evaluated as a secondary end point: a compliance of <60% of capsules that were supposed to be ingested would not be considered acceptable to validate the corresponding dose regimen. All patients but 1 received the full treatment sequence, demonstrating good study drug compliance. One patient (cohort 4) was not included in the per-protocol analysis, given that he received <60% of the study drug owing to a serious AE (SAE), an infectious diarrhea caused by enteropathogenic *Escherichia coli* (EPEC), leading the investigator to withdraw the patient owing to safety considerations. This event was evaluated by the independent DSMB and was considered a DLT.

The overall safety was the main secondary end point and AEs were monitored throughout the study in all 21 treated patients. In total, 136 AEs occurring after the first administration of MaaT033 were reported in 19 patients during the CIMON study, 83% of which were considered mild or moderate (supplemental Table 6). The remaining 2 patients did not develop AE. The most frequent AEs are presented in Table 2 and were expected toxicities in patients recovering from IC or receiving consolidation chemotherapy, including GI AEs (supplemental Table 7). Four SAEs were reported in 4 patients after MaaT033 treatment (supplemental Table 8): 2 in cohort 2 (hyperkalemia owing to potassium intake, febrile neutropenia during consolidation), 1 in cohort 3 (neutropenic colitis during consolidation), and 1 in cohort 4 (infectious diarrhea caused by EPEC leading to study drug withdrawal). This infectious diarrhea was caused by EPEC, considered an SAE, and appeared 3 days after the patient started MaaT033. The drug was withdrawn and the patient fully recovered with appropriate treatment. This patient’s fecal analysis before MaaT033 treatment did not detect the presence of EPEC. Quality control of the MaaT033 batch administered to the patient was negative for EPEC, and 4 capsules randomly selected from the patient’s pillbox were all negative for EPEC by polymerase chain reaction analysis, suggesting a potential external contamination. EPEC infection from external/environment factors such as food is an expected toxicity after IC. Nevertheless, a formal relationship with MaaT033 could not be fully excluded and the event was considered by the investigator and the DSMB as probably related to the study treatment, based on timing.

**Table 2.** **Most frequent AEs and severe AEs**

Event	Any grade, n (%)	Grade ≥ 3, n (%)
Constipation	10 (48)	—
Thrombocytopenia	10 (48)	9 (43)
Abdominal distension	9 (43)	—
Diarrhea	7 (33)	1 (5)
Nausea	7 (33)	—
Abdominal pain	6 (29)	—
Gastroesophageal reflux disease	5 (24)	—
Anemia	4 (19)	3 (14)
Pyrexia	4 (19)	1 (5)
Febrile neutropenia	3 (14)	3 (14)
Hemorrhoids	3 (14)	—
Blood phosphorus decreased	2 (10)	—
Decreased appetite	2 (10)	—
Deep vein thrombosis	2 (10)	—
Dizziness	2 (10)	—
Dry eye	2 (10)	—
Dyspepsia	2 (10)	—
Hypercholesterolemia	2 (10)	—
Hypertriglyceridemia	2 (10)	—
Hypokalemia	2 (10)	—
Mucosal inflammation	2 (10)	—
Rash	2 (10)	—

Data are n (%) per patient. Shown are the AEs occurring in ≥2 patients. AEs are classified by frequency and then by alphabetical order.

In total, 6 infectious AEs were reported during the study in 5 patients. Five infectious AEs were reported during the consolidation cycle, after the MaaT033 treatment period (supplemental Table 6); none of them were qualified as serious and potentially related and were an expected side effect of the consolidation chemotherapy. The sixth AE was the infectious diarrhea caused by EPEC.

### Clinical outcomes

Nineteen patients received consolidation chemotherapy after last MaaT033 administration, of whom 8 (42%) were treated with broad-spectrum antibiotics for febrile neutropenia or documented infection. At last follow-up during the study (D44 ± 10 days) and after consolidation chemotherapy for patients who received 1, 16 patients (76%) had a complete remission, and 5 (24%) were in relapse. No patient died during the period of study.

Long-term follow-up data after the end of study (D44 ± 10 days) were also collected, as a post hoc analysis. The median follow-up time for all patients was 23.2 months (range, 2.9-34.9). Twelve patients underwent alloHCT at a median of 5.7 months (range, 2.3-21.7) after inclusion. Among these patients, 3 developed grade 2 to 4 aGVHD: 1 grade 2 (skin stage 2, GI stage 1), 2 grade 3 (1 stage 2 skin and GI aGVHD, 1 stage 3 liver aGVHD). No patient developed moderate/severe chronic GVHD. Among the 12 patients who underwent alloHCT, 8 were still alive at last follow-up.

In total, 11 patients died. Death was caused by relapse in 4 patients, extensively drug-resistant *Pseudomonas aeruginosa* pneumonia in 1 patient, multiorgan failure in 2 patients, cerebral hemorrhage in 1 patient, fungal infection in 1 patient, pneumopathy in 1 patient, and an unknown cause in 1 patient. The overall survival rate was 71% at 6 months, 67% at 1 year, and 46% at 2 years.

### Gut microbiota reconstitution with MaaT033

**Figure 2.**
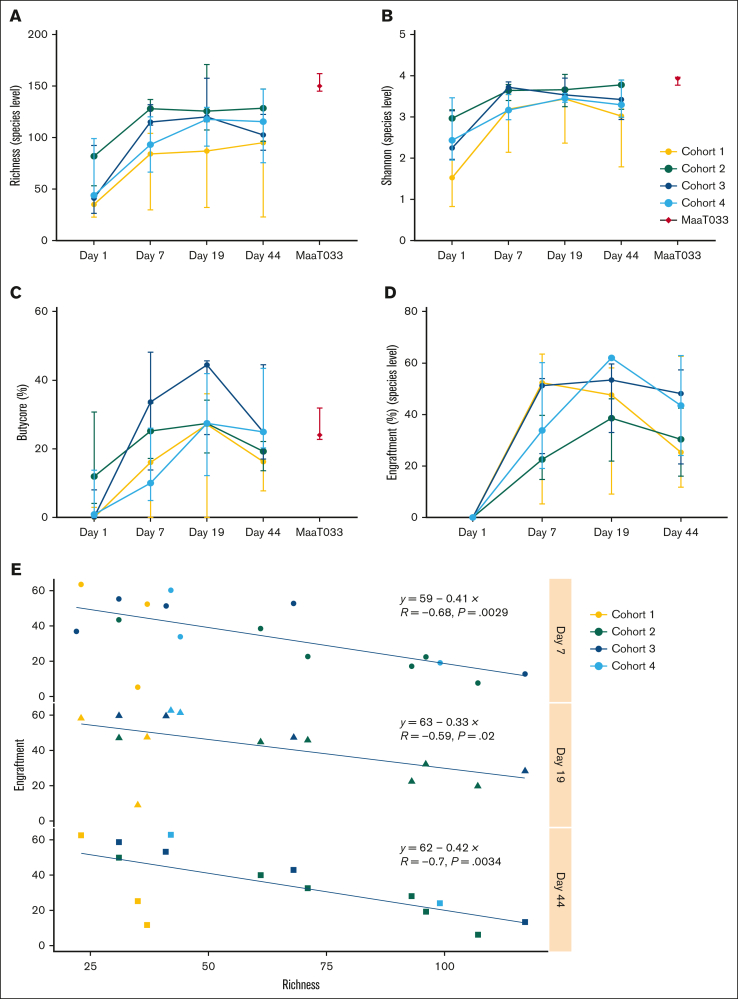
**MaaT033 impact on patients’ gut microbiota richness, engraftment, and Butycore levels relies on baseline microbiota (n = 21).** For each patient’s cohort, for MaaT033, and at each visit (D1, D7, D19, and D44): (A) Richness index at species level. (B) Shannon index at species level. (C) Relative abundance of Butycore, a group of 15 SCFA (mainly butyrate)-producing bacterial genera (*Blautia*, *Faecalibacterium*, *Alistipes*, *Eubacterium*, *Bifidobacterium*, *Ruminococcus*, *Clostridium*, *Coprococcus*, *Odoribacter*, *Roseburia*, *Anaerostipes*, *Oscillibacter*, *Subdoligranulum*, *Butyrivibrio*, *and Holdemanella*). (D) Engraftment at species level. The definition of engraftment is linked with the detection of donor-specific bacterial species that were not present in the patient’s feces before MaaT033 administration. For panels A to D, medians with interquartile ranges are provided. (E) Engraftment of MaaT033 at each visit by richness measured at D1 (at species level). Statistical significance was performed using Pearson correlation test.

**Figure 3.**
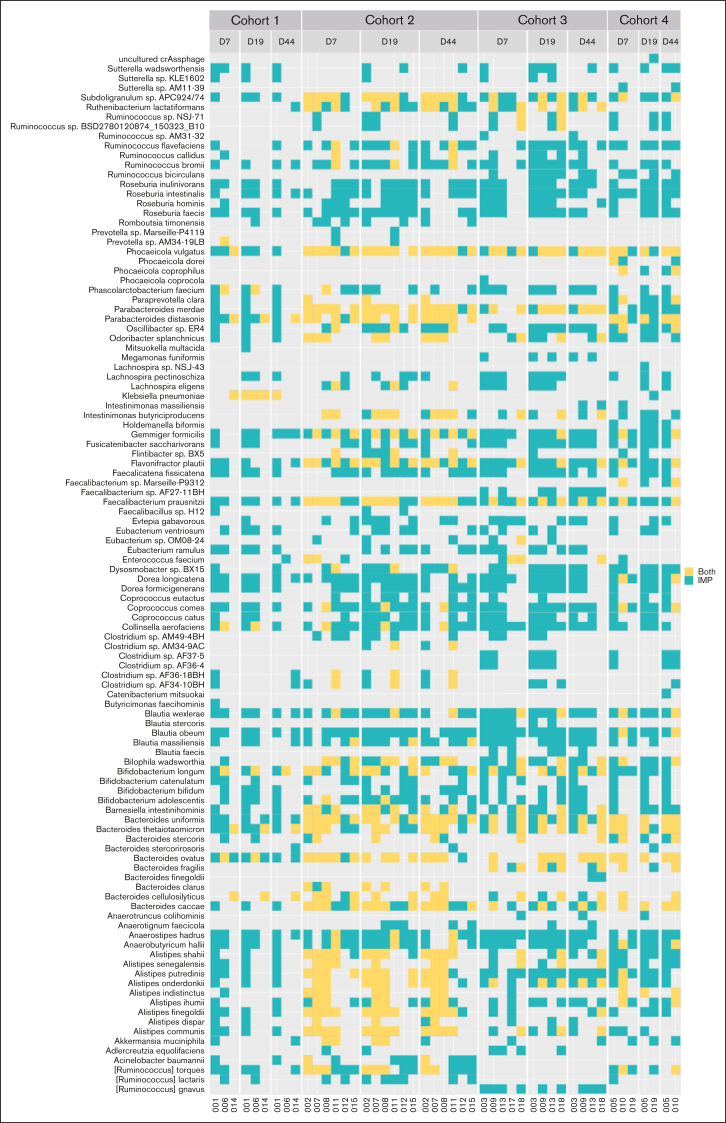
**List of species engrafted in patients.** Each column corresponds to a patient (n = 17), sorted by cohort (1, 2, 3, 4) and time point (D7, D19, D44). Each line corresponds to a species that engrafted in at least 1 patient at a given time point. Blue indicates that the species has engrafted (ie, present in the product and found in the microbiota of the patient at a given time point when not present at baseline), whereas yellow indicates that the strain was found in the microbiota of the patient at a given time point but is not considered engrafted because it was present in both the patient at baseline and the product.

Shotgun sequencing was performed on stool specimens collected at D1 (upon hematologic recovery from IC and before first MaaT033 administration), at D7 (during MaaT033 treatment), at D19 (after MaaT033 treatment completion), and at D44 after patients received subsequent consolidation chemotherapy. We found that, as expected, the gut microbiota of patients having undergone IC and broad-spectrum antibiotics was altered, with low α-diversity indices: richness index (species level, median, 44 for all patients) and Shannon index (species level, median, 1.52 for all patients) (Figures 2A and 3B). Treatment with MaaT033, at all dose levels, was associated with an increase of gut microbiota richness and diversity, as demonstrated by the increase of α-diversity indices in all cohorts. In particular, patients who received 3 capsules per day of MaaT033 (cohorts 3 and 4) had a sustained increase of richness and Shannon indices, up to D44 compared to patients who received only 1 capsule of MaaT033 weekly for 2 weeks. Of note, in cohort 2, the baseline richness index was higher, making it difficult to compare this cohort with the others.

Furthermore, the Bray-Curtis similarity index showed that MaaT033 treatment induced a shift in patients’ gut microbial communities, the composition of which became closer to MaaT033 composition as soon as D7 (supplemental Figure 3). We also looked at the relative abundance of Butycore, a group of 15 butyrate-producing bacterial genera. Treatment with MaaT033 was able to restore the Butycore from D7 in all cohorts, as demonstrated by the increase of this index to a level similar to that of the administered products (Figure 2C).

We then evaluated MaaT033 species engraftment in patients’ gut, by measuring the fraction of MaaT033 species (presence/absence, not relative abundance) found in patients’ feces coming specifically from the administered MaaT033 product. MaaT033 treatment induced a strong engraftment of MaaT033 species that persisted over time, even after consolidation chemotherapy, especially in cohorts 3 and 4 (Figure 2D). Interestingly, MaaT033 bacterial engraftment in patients at each visit was inversely correlated with their baseline microbiota richness (Pearson correlation test, *P* = .0029 at D7, *P* = .02 at D19, *P* = .0034 at D44) (Figure 2E).

The analysis of the species that engrafted showed that 108 different species were specifically engrafted in the patients, and product-specific species implanted in patients differed from 1 patient to another (Figure 3). This suggests that having a diversified product allows the engraftment of the microorganisms from the pool that is relevant to the patient’s ecosystem. Furthermore, we found that a significant number of bacteria that produce short-chain fatty acids (SCFAs) (particularly butyrate) engrafted. Indeed, 14 of the 15 genera composing the Butycore were present in MaaT033 and all engrafted in patients. We also found that, once a species had engrafted, it was often found in the same patient at subsequent time points, illustrating the persistence of the engraftment over time. Indeed, for 16 patients for which engraftment could be measured at all time points, an average of 60.7% of the species that engrafted at D9 were maintained in the patients’ microbiota at D19 and D44.

**Figure 4.**
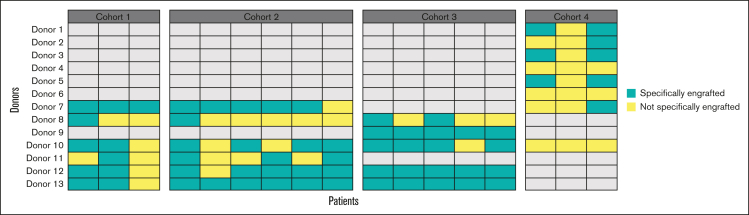
**Number of donors determined to have specifically contributed to the engraftment of MaaT033 species in patients’ gut (n = 17).** For cohorts 1 and 2, MaaT033 batch comprised fecal material from 6 individual donors. For cohort 3, MaaT033 batch comprised fecal material from 5 individual donors. For cohort 4, MaaT033 batch comprised fecal material from 8 individual donors. The definition of specific engraftment (in green) is linked with the detection of donor-specific bacterial species and does not include the bacteria, which may be shared by at least 2 donors (ie, not specifically engrafted, in yellow).

Regarding the effect of pooling fecal material from healthy donors on bacterial engraftment, 11 of the 13 donors provided specific species that engrafted in patients in a stochastic manner, and there was no single donor for which at least 1 bacterial species engrafted specifically in all patients. This illustrates the absence of a superdonor profile in the study and a possible “recipient effect,” that is, a compatibility between a donor’s microbial community and specific host factors, which is not yet predictable (Figure 4). In addition, for all donors, we found engraftment of bacteria species shared by several donors of the batch, precluding us from knowing which donor (1 or several) contributed the bacteria that engrafted. Of note, in cohort 4, the batch used was constituted by 8 donors leading to nonspecific engraftment for most donors.

Finally, we mapped the total metagenomic sequencing reads on the MEGARes antibiotic resistance gene database to evaluate the presence of antibiotic resistance-related genes. We found evidence that all patients had various proportions of several reads mapped against antibiotic resistance genes, especially against β-lactam resistance genes (supplemental Figure 4A-B). MaaT033 treatment was associated with a reduction in the number of reads mapped over time, including at D44 after consolidation chemotherapy, suggesting that MaaT033 had a beneficial effect in reducing resistome carriage induced by IC and antibiotics.

### MaaT033 promotes an increase in intestinal SCFAs

Using shotgun metagenomic data, we found that fecal acetate, propionate, and butyrate were increased during and after MaaT033 treatment, indicating that increased gene richness (and particularly those involved in SCFA metabolism observed after MaaT033) translates to increased levels of SCFAs in the stool (supplemental Results; supplemental Figures 4-6).

### MaaT033 is associated with a decreased inflammatory profile

**Figure 5.**
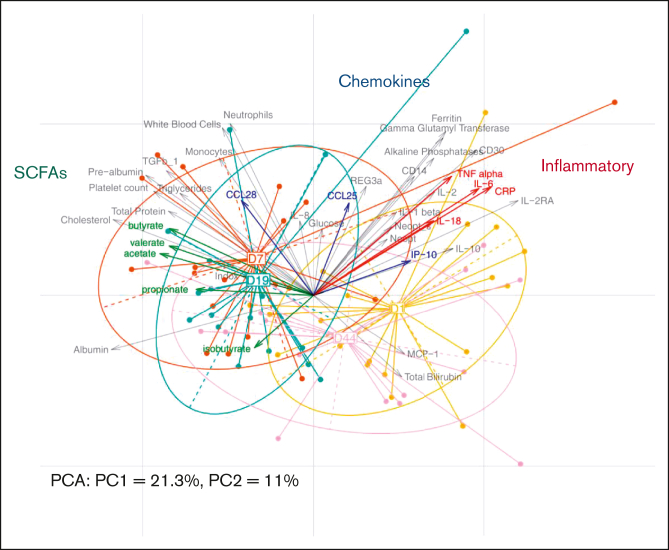
**PCA performed with the host parameters data set (n = 21).** Individual samples are represented by points colored and linked according to the corresponding visit. The arrows depict the variables, here the host parameters. The longer they are, the more they drive the samples’ projections; the closer they are, the more they vary together. Specific parameter groups are highlighted in color (SCFAs, chemokines, and inflammatory parameters). For a better visualization, variables with negligible impact on sample projections (with small arrows) were removed from the figure after computation. CRP, C-reactive protein; IL, interleukin; IP, Interferon gamma-induced protein 10; MCP-1, monocyte chemoattractant protein 1; PC1, principal component 1; PCA, principal component analysis; TNF, tumor necrosis factor.

The multivariate analysis shows the structuration of a pattern related to each follow-up day (D1, D7, D19, and D44) (Figure 5). This pattern is mostly directed by the horizontal axis (principal component 1, 21.3%) as it explains twice as more variability as the principal component 2 (11%). The center of the classes that group the patients per follow-up day forms a kind of trajectory from D1 to D44, starting with the initial state (D1), with a relative inflammatory profile that is higher values of C-reactive protein, interleukin-6, tumor necrosis factor α, interleukin-18, and neopterin (fecal and plasma). Then, after receiving MaaT033, we can observe a switch in the structure of a patient’s microbiome (on the left side of the figure) at D7 and preconsolidation measurements (D19). At these 2 time points, after MaaT033 treatment, there is an increase in the level of SCFAs in fecal samples, known to decrease the inflammation. We also observed an increase in other parameters such as albumin, indoxyl sulfate, and cholesterol. Finally, after consolidation therapy, the general status of the patients moved to an intermediate state between the initial (baseline) and the post-MaaT033 treatment state, which can be explained by the systemic and local deleterious effect of the consolidation chemotherapy.

## Discussion

The results of this current phase 1b study demonstrate that the use of outpatient lyophilized fecal pooled microbiota therapy after IC and antibiotic use in patients with AML is feasible and safe in immunocompromised patients. Only 1 patient developed a DLT in cohort 4, an infectious diarrhea caused by EPEC, that led to study drug withdrawal. Although we cannot formerly exclude a relationship with MaaT033, extensive quality control ruled out direct transmission of EPEC by MaaT033. Furthermore, diarrhea caused by EPEC is a well-established complication in patients treated for hematologic malignancies,^22^^,^^23^ and it was evidenced that most EPEC in patients with cancer with diarrhea are acquired in the community.^24^ Besides EPEC, 5 infectious AEs were reported, all during consolidation chemotherapy, after MaaT033 treatment, and were not considered to be related to MaaT033. Of note, the overall survival rate was 67% at 1 year and 46% at 2 years, which is comparable with the overall survival reported in randomized phase 3 studies with a similar induction chemotherapy (cytarabine + anthracycline), ranging from 41.9% to 60%.^25^^,^^26^

As previously reported by us and others, IC and broad-spectrum antibiotics in patients with AML are associated with an important disruption of the intestinal microbiota.^18^^,^^27^ Here, we show for the first time that outpatient administration of lyophilized oral pooled microbiotherapy is efficient in restoring gut microbiota richness and diversity of patients with AML to a level similar to that of healthy donors. In particular, although we found that, in cohorts 1 and 2, an increase in richness and α diversity was already observed, the use of higher doses of MaaT033 in cohorts 3 and 4 (3 capsules per day for 1 or 2 weeks) translated into a sustained increased richness and α diversity up to D44, despite patients receiving consolidation chemotherapy and broad-spectrum antibiotics after completing MaaT033 treatment. Repeated injury to the microbiome leads to sustained alteration of the gut microbiota that may not be able to restore to normal,^28^ and it was shown in patients with AML who underwent intensive chemotherapy, microbiota shift up to at least 6 months.^20^ Therefore, our data suggest that MaaT033, by restoring the gut microbiota richness and alpha diversity before further injury (consolidation chemotherapy), may improve the resilience of the microbiome. In addition, only 42% of patients were treated with broad-spectrum antibiotics for febrile neutropenia or documented infection during consolidation chemotherapy, which compared favorably with an expected febrile neutropenia rate ranging from 50% to 70%,^29^ further suggesting a protective effect of MaaT033, probably owing to the restoration of the microbiome.

Our study being nonrandomized with no placebo control arm, we cannot exclude that GI microbiota richness and α diversity would have increased, at least partially, in the absence of MaaT033. Nevertheless, given the fact that in patients with AML undergoing IC there is a long-term microbiota shift up to 6 months,^20^ but also that loss of bacterial diversity before alloHCT is now well established, further confirming the prolonged effect of IC and antibiotic on microbiota composition,^6^ these suggest that in the absence of MaaT033 treatment richness and α diversity would not have recovered to the level observed in our study.

Furthermore, we found a good engraftment of MaaT033, with an increase of butyrate-producing bacteria. In particular, patients who received 3 capsules of MaaT033 per day for 1 or 2 weeks showed persistent engraftment and increased levels of butyrate-producing bacteria. Importantly, despite butyrate level decrease at D44, levels remain higher than the level after IC and close to the level of MaaT033 product, suggesting that, despite consolidation chemotherapy and broad-spectrum antibiotics, MaaT033 may improve the resilience of the microbiome and preserve at least partially the microbiome composition in addition to the richness and α diversity.

Of note, engraftment was evaluated at the species level and not at the strain level; therefore, we cannot definitively prove that engrafted bacteria originated from MaaT033. Given that patients were included at neutrophil recovery after IC, no samples collected before beginning of IC were available, and bacteria potentially resilient after IC and use of broad-spectrum antibiotics may have been mistakenly considered as MaaT033 derived, leading to an overestimation of MaaT033 engraftment. Nevertheless, in our study, MaaT033 activity was based not only on engraftment but also on colonization of the GI tract, which was achieved irrespective of the origin of the bacteria. Overall, given that cohorts 3 and 4 (3 capsules per day for 1 or 2 weeks) translated into a sustained engraftment, with an increased richness and α diversity but also a butyrate level up to D44, despite patients receiving consolidation chemotherapy and broad-spectrum antibiotics, in future studies, patients will receive 3 capsules of MaaT033 once a day. Therefore, the maximum tolerable dose of MaaT033 was not determined because the interim results suggested adequate efficacy as measured by engraftment at lower doses (3 capsules per day). No expansion cohort was planned in this study given that a randomized phase 2b study was already schooled.

Importantly, pooling of fecal material from healthy donors probably contributed to the colonization of the GI tract, given that 11 of 13 donors provided specific species engraftment in patients in a stochastic manner with no single donor for which at least 1 bacterial species specifically engrafted in all patients. Therefore, there is no superdonor profile, and on the contrary, pooling of several donors allows a higher engraftment rate through the engraftment of multiple species from different donors, in particular butyrate-producing bacteria.

We also found that the switch in the structure of a patient’s microbiome after MaaT033 administration is associated with an increase in SCFAs in fecal samples and a decrease in systemic inflammation. We were able to demonstrate that, after MaaT033 administration, this increase in SCFAs (butyrate and acetate) was related to a microbiome shift, as evidenced by the significant correlation between KEGG functions known to be involved in SCFA metabolism and SCFA production (butyrate and acetate). Finally, the decreased systemic inflammation observed may, at least partially, be explained by the increased SCFA levels, which are well-established inflammation regulators.^30^

Our findings are in accordance with our previous study using autologous fecal microbiota therapy as an enema in the same setting,^18^ with α-diversity indices returning to their initial mean levels and a similarity index that showed restoration of microbial communities. Rashidi et al^31^ recently reported on a randomized, double-blind, phase 2 trial of fecal microbiota transplantation (FMT) vs placebo after IC for AML (n = 26) or alloHCT (n = 74). In that trial, FMT consisted of 5 frozen freeze-dried capsules taken all at once. Similar to our study, FMT was proven to be safe and ameliorated patients’ intestinal dysbiosis. Although the primary end point of their study was not reached, with no reduction of the all-cause infection rate within 4 months after FMT, the inclusion of infection unrelated to the gut bacterial microbiota in that end point might have diluted a potential protective effect of FMT.

Overall, lyophilized, pooled full-ecosystem fecal microbiota MaaT033 seems to be safe and effective for gut microbiota restoration and translates into increased SCFA levels. Of note, 1 limitation of this study was the limited sample size subsequent to the phase 1 study design, preventing us from performing a statistical analysis and adjusting for multiple testing of the differences that we observed in the microbiome and also in metabolites or immunity parameters. Furthermore, it will be important in future studies to investigate more closely the systemic effect of MaaT033 on metabolites and immune cells.

Finally, given the growing evidence that links gut microbiota dysbiosis to outcomes of patients with cancer, in particular after alloHCT,^6^ chimeric antigen receptor T-cell therapy,^8^ and immunotherapy for solid tumors,^32^ off-the-shelf microbiotherapy treatments are needed and warrant investigation. Based on this phase 1 study, MaaT033 is currently being tested in a randomized, placebo-controlled, phase 2b trial, which aims to improve survival through the prevention of transplant-related complications in eligible alloHCT patients (ClinicalTrials.gov identifier: NCT05762211).

Conflict-of-interest disclosure: F.M. reports honoraria from Bristol Myers Squibb (BMS), Therakos/Mallinckrodt, Sanofi, Priothera, Novartis, AstraZeneca, and Merck Sharp & Dohme, all outside the submitted work. S.T. reports honoraria from BMS, Gilead, Astellas, and Therakos/Mallinckrodt. T.C. reports clinical research support from Novartis, Alexion, Celgene/BMS, Amgen, Syros, Kartos, Arog, Takeda, Servier, Janssen, and Keros; consulting or advisory role with BMS/Celgene, AbbVie, Jazz Pharmaceuticals, Novartis, Agios, Servier, Astellas, Incyte, and BluePrint; and support for attending meetings and/or travel from Pfizer, Celgene/BMS, Novartis, AbbVie, Servier, and Gilead. M.L. reports honoraria from AstraZeneca, Alexion, BMS/Celgene, Pfizer, Gilead, Novartis, Kartos, Telios, GSK, Stemline, Incyte, Merck Sharp & Dohme, and Janssen. M. Meunier reports honoraria from BMS, Novartis, Alexion, Pfizer, and GSK, all outside the submitted work. E. Plantamura, B.L., and C.G. are employees of MaaT Pharma and have patent WO2020/016445 A1. J.J., E. Prestat, and A.S. are employees of MaaT Pharma. J.D. is a cofounder and a member of the advisory board of MaaT Pharma; reports consulting fees from MaaT Pharma; reports support for attending meetings and/or travel from MaaT Pharma; and reports shares from MaaT Pharma and patents WO2016/170285 A1, WO2016/170290 A1, and WO2017/103550 A1. C.R. declares a consulting or advisory role with AbbVie, Amgen, Astellas, BMS, Boehringer, Jazz Pharmaceuticals, and Servier; reports receiving research funding from AbbVie, Amgen, Astellas, BMS, Iqvia, Jazz Pharmaceuticals, and MaaT Pharma; and reports support for attending meetings and/or travel from AbbVie, Novartis, and Servier. M. Mohty reports grants, lecture honoraria, and research support from Adaptive Biotechnologies, Amgen, Astellas, BMS/Celgene, GlaxoSmithKline, Janssen, Jazz Pharmaceuticals, Novartis, Pfizer, Takeda, and Sanofi, all outside the scope of this work. The remaining authors declare no competing financial interests.

## Supplementary Material

Supplemental Methods, References, Tables, and Figures

Supplemental Methods and Appendices

Supplemental Methods, Tables, Figures, References, and Appendices
