# Supplementary material for: Gut microbiota restoration with oral pooled fecal microbiotherapy after intensive chemotherapy: the phase 1b CIMON trial
Source: Blood Adv. 2025 Apr 10;9(15):3739–49. doi: 10.1182/bloodadvances.2024015571 (PMC12305571; doi:10.1182/bloodadvances.2024015571)
Supplement: Supplemental Methods, References, Tables, and Figures [file BLOODA_ADV-2024-015571-mmc1.pdf]

Restoration of gut microbiota diversity with oral pooled fecal microbiotherapy after intensive chemotherapy: the phase Ib CIMON trial.

Florent Malard[1], Sylvain Thepot[2], Thomas Cluzeau[3], Martin Carré[4], Delphine Lebon[5], Pierre Bories[6], Ollivier Legrand[1], Marianne Schwarz[2], Michael Loschi[3], Mathieu Meunier[4], Magalie Joris[5], Cyrielle Gasc[7], Juliette Jouve[7], Benoit Levast[7], Emilie Plantamura[7], Emmanuel Prestat[7], Antoine Sabourin[7], Béatrice Gaugler[1], Joel Dore[8], Christian Récher[6*] , Mohamad Mohty[1*]

[1] Sorbonne University, Department of Clinical Hematology and Cellular Therapy, Saint-Antoine Hospital, Assistance Publique - Hôpitaux de Paris, INSERM UMRs 938, Centre de Recherche Saint-Antoine (CRSA), Paris, France
[2]Clinical Hematology, Angers University Hospital, Angers, France
[3]Hematology department, Côte D'Azur University, Nice Hospital, Nice, France
[4]Hematology department, Grenoble Alpes University hospital, La Tronche, France
[5]Hematology department, Amiens University Hospital, Picardie, Amiens, France
[6]Hematology department, Toulouse University Hospital, Institut Universitaire du Cancer de Toulouse Oncopole, Toulouse III Paul Sabatier University, Toulouse, France
[7]MaaT Pharma, Lyon, France
[8] Paris-Saclay University, INRAE, MetaGenoPolis, Jouy-en-Josas, France
* These authors share senior authorship.

Supplementary materials

Supplementary methods

CIMON trial overview

The trial was designed, and data were analyzed by the investigators in collaboration with the sponsor (MaaT Pharma) and the Trial Steering Committee. All authors vouch for the accuracy and completeness of the data and for the fidelity of the trial to the protocol (the protocol is provided as a Supplementary Information file). It was conducted in accordance with the guidelines for Good Clinical Practice of the International Council for Harmonization, with applicable local regulations, and according to the principles of the Declaration of Helsinki. First-in-human use of the pooled allogenic oral microbiotherapy MaaT033 in a phase I trial was approved by the French health regulatory authorities on February 26th, 2020, and by the relevant independent ethics committee "Ile de France I" on February 28th, 2020 (approvals are provided in the Supplementary Information file). The study was registered at ClinicalTrials.gov (Identifier: NCT04150393) with an initial release on November 4th, 2019, before the first patient inclusion.

The first patient was enrolled on October 19th, 2020; the last one on November 3rd, 2021, and the last patient's final visit took place on December 13th, 2021. Patients were screened and recruited across six French hospitals.

CIMON study population

Eligible patients were adults with newly diagnosed AML (as defined by WHO 2016 criteria with ≥20% leukemic blasts in the bone marrow) or high-risk myelodysplastic syndrome (HR-MDS), receiving IC, and who were considered healthy enough to likely receive their consolidation or second chemotherapy cycle and to likely receive alloHCT, depending on the AML or MDS characteristics. Patients receiving azacytidine combined with 28 days venetoclax induction were also eligible. Patients were not included if they had a history of digestive disorders (gastro-intestinal [GI] surgery or bleeding 3 months prior to inclusion,

confirmed or suspected intestinal ischemia, toxic megacolon or GI perforation), and/or suffered from other severe disease including inflammatory bowel disease. Patients achieving neutrophil recovery (absolute neutrophil count, ANC ≥ $0.5.10^9$ cells/L) after IC, fulfilling all criteria and that signed the informed consent were included

MaaT033 production

MaaT033 is manufactured in a French cGMP manufacturing facility by pooling fecal material from vetted, healthy donors. The safety testing strategy for healthy donors comprises medical evaluation, regular testing of blood and feces, following current regulatory recommendations for safety testing [1,2] (**Supplementary Table 2** for complete list of screening tests). The overall manufacturing process for MaaT033, which is similar to that for a MaaT013 enema, previously described [3], with the addition of the lyophilization and encapsulation steps, ensures similar properties and compositions between the two products (**Supplementary Figure 1**). The fecal material from each donor is collected in a specific device, diluted with an adapted volume of cryoprotective diluent by mixing, and in the same process filtered with a 265µm sieve. The resulting solutions from 4 to 8 donors are mixed, and then transferred and stored in a container (Lyoguard®) specifically conceived to withstand storage at -40°C and support the freeze-dry process. After freeze-drying, the product is cracked and milled to a powder, filled in specific capsules, allowing ileo-cecal release. Each capsule of MaaT033 contains a minimum of 10^9 viable cells (absolute number).

The capsule was developed using an *in vitro* model of the human colon reproducing medically relevant perturbation of the colonic ecosystem by antibiotherapy, and we previously showed that the capsule was as efficient as an enema in restoring the gut microbiota structure and activity [4].

The overall manufacturing process for MaaT033, which is similar to that for a MaaT013 enema, previously described [3], with the addition of the lyophilization and encapsulation steps, ensures similar properties and compositions between the two products (**Supplementary Figure 1A**).

For both products, pooling fecal material from multiple healthy donors allows the standardization of the microbial profile of manufactured products with a similar microbial community structure in terms of present taxa and relative abundances (**Supplementary Figures 1B and 1C**), and a significantly increased diversity, as evaluated by the Shannon index, when compared to individual donors (**Supplementary Figure 1D**).

Escalation rules for phase 1 design:

The study has an adapted "6 dose titration" escalation design to allow for assessment of tolerability (research of the Maximal Tolerated Dose) and of activity (research of Minimal Active Dose):

- 1st level of dose for 1st cohort: - 3 patients will be treated with one capsule/day on Day 1 and Day 7. The Safety Committee (SC) will evaluate occurrence of Limiting Toxicity after these 3 patients will have completed Visit 3. If no patient experiences a DLT, the following cohort will use dose level 2 *i.e.* 1 capsule/day for 7 days. If one or more patient experiment a DLT, the SC might consider study stop recommendation or cohort continuation. The purpose of this cohort is to assess feasibility of the whole procedure and check any unexpected tolerability issue that would prohibit continuation of the experimentation. As the dose schedule is not considered as sufficient enough to allow for any effective activity, it will not be considered for the choice of final dose.
- 2nd level of dose (cohort2): 6 patients will be treated with 1 capsule/day for 7 days. The SC will evaluate occurrence of Limiting Toxicity after the 6 patients will have completed Visit 3.

If no or 1 patient experiences a DLT, the following cohort will use dose level 3 *i.e.* 3 capsules/day for 7 days. If 2 or more patients out of 6 experience a DLT, the study will be stopped.

- 3rd level of dose (cohort3): 6 patients will be treated with 3 capsules/day for 7 days. The SC will evaluate occurrence of Limiting Toxicity after the 6 patients will have completed Visit 3. If no or 1 patient experiences a DLT, the following cohort will use dose level 4 *i.e.* 3 capsules/day for 14 days and dose level 5 *i.e.* 9 capsules/day for 7 days. If 2 or more patients out of 6 experience a DLT, the study will be stopped.

- 4th level of dose: 6 patients will be treated with 3 capsules/day for 14 days. The SC will evaluate occurrence of Limiting Toxicity after the 6 patients will have completed Visit 3. If no or 1 patient experiences a DLT, the following cohort will use dose level 5 *i.e.* 9 capsules/day for 7 days. If 2 or more patients out of 6 experience a DLT, the study will be stopped.

Of note, if 2 or more DLTs are reported during the first 7 days of treatment, then the SC will re-assess cohort 3 evaluation.

- 5th level of dose: 6 patients will be treated with 9 capsules/day for 7 days. The SC will evaluate occurrence of Limiting Toxicity after the 6 patients will have completed Visit 3. If 2 or more patients experience a DLT, the study will be stopped and MTD will be considered as dose level 4. If one patient or less experiences a DLT, the MTD will be considered dose level 5.

In any of the cohorts 2 to 5, if any safety signal suggesting a potential DLT is reported in 2 or more patients before the end of the inclusion of all patients of the cohort, inclusion of subsequent patients will be suspended and an *ad hoc* meeting of the DSMB will be held with all available information to assess these potential DLTs and decide on continuing or stopping the study.

Any dose level higher than dose level 1 may be considered for choice of the final dose.

A 24-hour gap should be respected between inclusions of the first 3 patients of each cohort to consider potential early or delayed toxicities.

Immune and fecal parameter analyses

Blood and fecal parameters were analyzed in a central laboratory using the following methods: fecal secretory IgA (ELISA - ImmunoChrom kit RIC6100), fecal neopterin (ELISA - Kit Neopterin Elisa Ref.RE59321 / IBL International GmbH), fecal zonulin (IDK Zonulin ELISA ref. K 5600 / Immundiagnostik AG), fecal calprotectin (Immunoturbidimetry; kit fCAL Turbo Calprotectin ref. BKCAL-REST / BÜHLMANN), fecal short-chain fatty acids (SCFAs) (Gas-chromatography – mass spectrometry), fecal bile acids (Liquid chromatography – mass spectrometry / mass spectrometry), plasma cytokines :TGFβ1,2,3; sCD14, IL1b, IL2, sIL2-ra, IL6, IL8, IL10, IL17A, IL18, IFNg, TNFα, MCP1, CCL25, CCL28, sCD30, CXCL10, RegIIIa (Luminex 16 plex [Biotechne], Luminex TGFβ1,2,3) and sCD14 (Merck Millipore)) and plasma neopterin (ELISA - Kit Neopterin Elisa Ref.RE59321 / IBL International GmbH).

Other blood parameters (complete blood count, high-sensitivity C-reactive protein (CRP) [hs-CRP], ferritin, uric acid, urea, albumin, pre-albumin, total protein, triglycerides, lactate dehydrogenase [LDH], cholesterol, total bilirubin, bilirubin direct, alanine leucine transferase [ALT], alanine serine transferase [AST], gamma glutamyl transferase [GGT], alkaline phosphatases, serum glucose) were analyzed locally at sites.

DNA isolation and shotgun metagenomic sequencing

Genomic DNA was extracted from the fecal samples collected during the first four visits using the Macherey-Nagel NucleoSpin Soil kit. A shotgun sequencing library was constructed for each DNA sample, and libraries were then sequenced in a 2 x 150bp NovaSeq2500 run

(Illumina) to generate 30 million pairs of reads per sample. Positive and negative controls were added by the sequencing service provider throughout the process to validate the successful completion of each step.

Shotgun bioinformatics analyses

Shotgun bioinformatics analyses were performed on the gutPrint® platform with the in-house MgRunner v1.4.0 pipeline. In brief, after quality filtering using Trimmomatic [5], host sequence decontamination was performed with Bowtie2 [6]. To ensure comparability, all samples were rarefied to the same sequencing depth. Taxonomic profiling was performed with Kraken2 [7] and the RefSeq genomic database (2020 release, http://www.ncbi.nlm.nih.gov/refseq/). The measurements of α- and β-diversity indices were performed with R Statistical Software (http://www.R-project.org) using vegan and phyloseq packages [8]. Functional profiling was then performed using the NGLess framework [9] with the IGC gene catalog[8] annotated with KO (KEGG Orthology) identifiers [10]. Finally, antibiotic resistance analyses were performed through gene mapping with Bowtie2 on MEGARes database (https://megares.meglab.org/).

Host parameters and fecal microbiota multivariate analyses

Analysis of host parameters

The objectives were to inspect the host parameter covariations and how they are impacted by MaaT033 treatment by the means of a dimension reduction method.

A series of 55 host parameters was measured in the 20 patients at baseline (D1), D7, D19 and D44. A centered and scaled principal component analysis (PCA) was performed on a subset of parameters. Each of the variable distributions was visually inspected with histograms to ensure that they were bell-shaped before applying the PCA procedure. A log(x+1) transformation was applied to the parameters with a dominant density for low values. IFNγ, IL-17A, TGFβ3, eosinophils, basophils, bilirubin direct were excluded from the analysis at this stage as most of their values were zeros. For 21 of the remaining variables, the

observations were not complete for all patients, so an Expectation-Maximization (EM) (with 1000 iterations) imputation algorithm ("imputePCA" from the missMDA R package [11]) was executed before the application of the multivariate analysis using the ade4 R package.

The 55 measured host parameters were: in the stool: fecal SCFAs (acetate, isobutyrate, butyrate, valerate), fecal neopterin, fecal IgA and fecal zonulin, and in the blood: indoxyl sulfate, TGFβ1, TGFβ2, TGFβ3, soluble CD14, MCP-1, CCL-28, IL-2RA, IFNγ, IL-2, IL-8, IL-17A, REG3α, CCL-25, CD30, IP-10, IL-1β, IL-6, IL-18, TNFα, neopterin, hs-CRP, ferritin, uric acid, urea, albumin, pre-albumin, total protein, triglycerides, LDH, cholesterol, total bilirubin, bilirubin direct, ALT, AST, GGT, alkaline phosphatases, serum glucose, red blood cells, white blood cells, neutrophils, lymphocytes, monocytes, eosinophils, basophils, platelets, hemoglobin, hematocrit.

Sample size justification

The CIMON study objective was to evaluate the first-in-man use of MaaT033 in AML / HR-MDS patients after IC by assessing its safety profile and determining a recommended phase II dose. As such, no formal sample size calculation has been made, but a step-up dosing design from 1 capsule per week to 9 capsules per day for 7 or 14 days was chosen, with 3 patients in the first cohort and 6 patients in the other cohorts.

Supplementary results

MaaT033 promotes an increase in intestinal short-chain fatty acids

Using shotgun metagenomic data, we also investigated whether in addition to a reconstruction of the structure of the patients' microbiota, the metabolic potential of their communities was also restored. We first looked at the gene richness which corresponds to the number of different genes found in the microbiota and thus reflects the functional potential of microbial communities (**Supplementary Figure 4C**). At D1, the gene richness is low in these patients, which reflects their dysbiotic state. Gene richness is then increased from D7 after the first treatment administration, to reach a level similar to that of the products at D19 and D44 for cohorts 2, 3 and 4. Furthermore, we compared the abundances of metagenomic reads that match KEGG functions known to be involved in SCFA (here acetate, propionate and butyrate) metabolism (**Supplementary Figure 4D**). Among those, 2 trends and 2 significant correlations were found by comparing with acetate, and 4 trends and 3 significant correlations were detected with a butyrate metabolite. These identified KOs are important in production of both substrates production as observed with the maps of the corresponding acetate and butyrate pathways (**Supplementary Figures 5A and 5B)**. Finally, we found that fecal acetate, propionate, and butyrate were increased during and after MaaT033 treatment, indicating that increased gene richness (and particularly those involved in SCFA metabolism observed after MaaT033) translates to increased levels of SCFAs in the stool (**Supplementary Figure 6).**

References

1. santé Andsdmedpd. La transplantation de microbiote fécal et son encadrement dans les essais cliniques. . 2016.
2. Cammarota G, Ianiro G, Tilg H, et al. European consensus conference on faecal microbiota transplantation in clinical practice. *Gut*. 2017;66(4):569-580.
3. Malard F, Loschi M, Huynh A, et al. Pooled allogeneic faecal microbiota MaaT013 for steroid-resistant gastrointestinal acute graft-versus-host disease: a single-arm, multicentre phase 2 trial. *EClinicalMedicine*. 2023;62:102111.

4. Verdier C, Denis S, Gasc C, et al. An Oral FMT Capsule as Efficient as an Enema for Microbiota Reconstruction Following Disruption by Antibiotics, as Assessed in an In Vitro Human Gut Model. *Microorganisms*. 2021;9(2).
5. Bolger AM, Lohse M, Usadel B. Trimmomatic: a flexible trimmer for Illumina sequence data. *Bioinformatics*. 2014;30(15):2114-2120.
6. Langmead B, Salzberg SL. Fast gapped-read alignment with Bowtie 2. *Nat Methods*. 2012;9(4):357-359.
7. Wood DE, Salzberg SL. Kraken: ultrafast metagenomic sequence classification using exact alignments. *Genome Biol*. 2014;15(3):R46.
8. Li J, Jia H, Cai X, et al. An integrated catalog of reference genes in the human gut microbiome. *Nat Biotechnol*. 2014;32(8):834-841.
9. Coelho LP, Alves R, Monteiro P, Huerta-Cepas J, Freitas AT, Bork P. NG-meta-profiler: fast processing of metagenomes using NGLess, a domain-specific language. *Microbiome*. 2019;7(1):84.
10. Kanehisa M, Furumichi M, Tanabe M, Sato Y, Morishima K. KEGG: new perspectives on genomes, pathways, diseases and drugs. *Nucleic Acids Res*. 2017;45(D1):D353-d361.
11. Josse J, Husson F. missMDA: A Package for Handling Missing Values in Multivariate Data Analysis. *Journal of Statistical Software*. 2016;70(1):1 - 31.

Supplementary Table 1: Complete list of inclusion and non-inclusion criteria

Criteria for inclusion	To be eligible for the trial, patients must meet all the following inclusion criteria:<br>1. Male or Female<br>2. Age ≥ 18 years<br>3. Patients diagnosed with AML defined according to WHO 2016 criteria with ≥20% leukemic blasts in the bone marrow or with high-risk myelodysplastic syndrome, receiving intensive chemotherapy<br>4. Patients healthy enough to likely receive their consolidation or second cycle of chemotherapy after induction chemotherapy<br>5. Patients healthy enough to likely receive HSCT<br>6. Informed written consent<br>7. Patient recovered from neutropenia
Criteria for non-inclusion	Patients meeting any of the following criteria will not be included in the trial:<br>1. Acute promyelocytic leukemia (AML-M3)<br>2. AML secondary to myeloproliferative disorder or chronic myelomonocytic leukemia (CMML)<br>3. AML BCR-ABL1+<br>4. Active central nervous system leukemia<br>5. Patients with a life expectancy of <70 days according to Investigator's opinion, or subject to therapeutic limitations<br>6. Confirmed or suspected intestinal ischemia<br>7. Confirmed or suspected toxic megacolon or gastrointestinal perforation<br>8. Active uncontrolled infection according to the attending physician<br>9. Any gastrointestinal bleeding in the past 3 months<br>10. Any history of gastrointestinal surgery in the past 3 months<br>11. Any history of inflammatory bowel disease<br>12. Any counter-indication to swallow capsules<br>13. Enrollment in another trial that may interfere with this study<br>14. Known allergy or intolerance to trehalose, maltodextrin or PEG<br>15. Women of childbearing potential without efficient contraceptive protection<br>16. Pregnant or breastfeeding<br>17. Patients with EBV-negative serology<br>18. Subject who, in the judgment of the Investigator, is likely to be non-compliant or uncooperative during the study, or unable to cooperate because of a language problem or poor mental development<br>19. Exclusion period of a previous study<br>20. Administrative or legal supervision<br>21. Confirmed positive result to SARS-CoV-2 test at screening

AML: Acute myeloid leukemia; HSCT: hematopoietic stem cell transplantation

Supplementary Table 2: List of screening tests performed on donors' blood and feces

**Parameter**		**Testing**
**In blood**		
Toxoplasma gondii	Anti-Toxoplasma gondii IgM antibody	ECLIA
Treponema pallidum	Treponema pallidum (treponemic & non treponemic test)	Immunochromatography/Ab detection
Cytomegalovirus (CMV)	Antibody IgG anti-CMV	CLIA
	Antibody IgM anti-CMV	CLIA
	Viral load quantification	PCR
Epstein-Barr virus (EBV)	EBV/EBNA-IgG	CLIA
	ABV/EA-IgG or VCA IgG	CLIA
	EBV/EA-IgM or VCA IgM	CLIA
Human immunodeficiency virus	HIV (Antibody against, HIV-1, Antibody against HIV-2, Antigen P24)	ECLIA
	HIV-1 Viral load quantification	PCR
Hepatitis B	Antibody anti-HBc	ECLIA
	Antibody anti-HBs	ECLIA
	Virus Surface antigen	ECLIA
	Viral load quantification	PCR
Hepatitis C	Antibody anti-VHC	ECLIA
	Viral load quantification	PCR
Hepatitis A	IgM and total antibodies	ECLIA
	Viral load quantification	PCR
Hepatitis E	Anti HEV IgM Ab	EIA
	Anti HEV IgG Ab	EIA
	Viral load quantification	PCR
Human T lymphotropic virus I and II (HTLV)	Antibody anti HTLV 1 / 2 qualitative	CLIA
	Viral load detection	PCR
**In feces**		
Extended-spectrum-β-lactamase-producing bacteria		Culture
Vancomycin / Glycopeptide-resistant enterococci		Culture
Listeria spp.		Culture
Carbapenemase-producing bacteria		Culture
Methicillin-resistant *Staphylococcus aureus*		PCR
Blastocystis hominis		Microscopic exam after concentrations
Dientamoeba fragilis		
Isospora spp.		
Strongyloides stercoralis		Microscopic exam after concentrations and Baermann technique
Helminths		PCR multiplex
Microsporidea		PCR
Cryptosporidium, Cyclospora cayetanensis, Entamoeba histolytica, Giardia lamblia, Campylobacter, Clostridium difficile (toxin A/B), *Salmonella, Yersinia enterocolitica, Vibrio cholerae, Vibrio,* Shiga toxin-producing *E.coli* (STEC) *stx1/stx2,* enteroaggregative *E. coli* (EAEC), enteropathogenic *E. coli* (EPEC), enterotoxigenic *E. coli* (ETEC) *It/st, Shigella/*enteroinvasive *E.coli* (EIEC)*, Plesiomonas shigelloides,* Adenovirus F40/41, Astrovirus, Norovirus GI/GII, Rotavirus A, Sapovirus, Hepatitis E, Hepatitis A, Enterovirus, SARS-CoV-2		PCR multiplex

ECLIA (electrochemiluminescence immunoassay), CLIA (chemiluminescence immunoassay), EIA (enzyme immunoassay, PCR (polymerase chain reaction)

Supplementary Table 3: Baseline demographic and diagnostic characteristics for each patient

Cohort	Patient number	Sex	Age at inclusion (years)	BMI at inclusion (kg/m²)	Cytogenetic risk (at diagnosis)	2022 ELN Risk stratification (at diagnosis)
**Cohort 1**	1	Male	44	33.1	Normal	Intermediate
	2	Male	63	25.7	Abnormal	Adverse
	3	Male	64	23.9	Normal	Intermediate
**Cohort 2**	4	Male	57	25.8	Abnormal	Intermediate
	5	Male	77	16.8	Abnormal	Adverse
	6	Male	22	30.0	Normal	Favorable
	7	Male	33	22.1	Abnormal	Adverse
	8	Male	52	22.0	Abnormal	Intermediate
	9	Male	66	22.1	Abnormal	Favourable
**Cohort 3**	10	Male	69	24.0	Normal	Adverse
	11	Male	37	30.1	Abnormal	Favourable
	12	Female	53	16.7	Normal	Favourable
	13	Female	48	21.3	Abnormal	Intermediate
	14	Male	61	21.1	Normal	Adverse
	15	Male	65	24.7	Abnormal	Favourable
**Cohort 4**	16	Female	66	21.7	Normal	Favourable
	17	Female	70	28.0	Abnormal	Intermediate
	18	Female	40	29.0	Normal	Adverse
	19	Male	62	20.5	Abnormal	Favourable
	20	Male	71	23.4	Normal	Adverse
	21	Male	67	25.8	Normal	Adverse

BMI: Body mass index, AML: acute myeloid leukemia

Risk stratification according to 2022 European LeukemiaNet (ELN) guidelines.

Supplementary Table 4: Antibiotic and chemotherapy received by each patient between the beginning of induction chemotherapy and first administration of MaaT033

Cohort	Patient number	Induction chemotherapy	Antibiotic name	Antibiotic classification	Antibiotic indication	Prophylaxis or therapeutic	Antibiotic dosage	Route of administration	Frequency	Antibiotic duration (days)
Cohort 1	1	Cytarabine + Idarubicin + Midostaurin + Venetoclax	Meropenem	Carbapenems	Infection on catheter (*Staphylococcus epidermidis*)	Therapeutic	1g	IV	TID	9
			Piperacillin-Tazobactam	Penicillins	Anti-infective prophylaxis	Prophylaxis	4g	IV	TID	25
			Amphotericin B	Macrolides	Antifungal prophylaxis	Prophylaxis	1 teaspoon	Per os	TID	41
			Daptomycin	Lipopeptides	Infection on catheter (*Staphylococcus epidermidis*)	Therapeutic	500mg	IV	QD	5
	2	Cytarabine + Idarubicin + Lomustine	Daptomycin	Lipopeptides	Bacteremia	Therapeutic	700mg	IV	QD	4
			Colistin	Polymyxins	Digestive decontamination	Prophylaxis	2 capsules	Per os	TID	24
			Piperacillin-Tazobactam	Penicillins	Anti-infective prophylaxis	Prophylaxis	4g	IV	PRN	15
			Amikacin	Aminoglycosides	Anti-infective prophylaxis	Prophylaxis	1200mg	IV	QD	1
			Vancomycin	Glycopeptides	Anti-infective prophylaxis	Prophylaxis	1g	IV	PRN	2
	3	Cytarabine + Idarubicin + Lomustine + Hydroxycarbamide + Methotrexate	Pristinamycin	Streptogramins	Furuncle	Therapeutic	1g	Per os	QD	27
			Cephalosporin	Cephalosporins	Infection of *Escherichia coli*	Therapeutic	Unknown	IV	Unknown	2
			Piperacillin-Tazobactam	Penicillins	Infection of *Escherichia coli*	Therapeutic	Unknown	IV	Unknown	2
			Vancomycin	Glycopeptides	Furuncle	Therapeutic	Unknown	IV	Unknown	2
			Cefotaxime	Cephalosporins	Furuncle	Therapeutic	2mg	IV	QD	11
Cohort 2	4	Cytarabine + Daunorubicin	Piperacillin-Tazobactam	Penicillins	Fever	Therapeutic	4g	IV	QID	1
			Cefepime	Cephalosporins	Sepsis	Therapeutic	2g	IV	TID	12
			Daptomycin	Lipopeptides	Sepsis	Therapeutic	720mg	IV	QD	4
			Amphotericin B	Macrolides	Oral care	Therapeutic	1000 mg	Mouthwash	TID	26
	5	Azacitidine + Venetoclax + Hydroxycarbamide	Meropenem	Carbapenems	Febrile neutropenia	Therapeutic	1 g	IV	TID	18
			Piperacillin-Tazobactam	Penicillins	Febrile neutropenia	Therapeutic	4g	IV	TID	16
	6	Cytarabine + Idarubicin	Ceftriaxone	Cephalosporins	Febrile aplasia	Therapeutic	2g	IV	QD	8
			Ceftazidime	Cephalosporins	Febrile aplasia with infection of	Therapeutic	5g	IV	QD	5

					Pseudomonas aeruginosa					
			Amikacin	Aminoglycosides	Febrile aplasia	Therapeutic	1.5g	IV	QD	2
	7	Cytarabine + Daunorubicin	Amphotericin B	Macrolides	Oral care	Prophylaxis	10 ml	Mouthwash	TID	1
			Daptomycin	Lipopeptides	Intermittent fever	Prophylaxis	700mg	IV	QD	32
			Imipenem	Carbapenems	Fever	Therapeutic	1g	IV	TID	11
			Piperacillin-Tazobactam	Penicillins	Intermittent fever	Therapeutic	4g	IV	QID	3
			Piperacillin-Tazobactam	Penicillins	Intermittent fever	Therapeutic	4g	IV	QID	30
			Amikacin	Aminoglycosides	Fever	Therapeutic	2100 mg	IV	QD	32
	8	Cytarabine + Idarubicin	Meropenem	Carbapenems	Febrile neutropenia	Therapeutic	1 g	IV	TID	7
			Piperacillin-Tazobactam	Penicillins	Febrile neutropenia	Therapeutic	4 g	IV	TID	4
			Daptomycin	Lipopeptides	Febrile neutropenia	Therapeutic	650 mg	IV	QD	4
	9	Cytarabine + Idarubicin	Piperacillin-Tazobactam	Penicillins	Anti-infective prophylaxis	Prophylaxis	4g	IV	QD	4
			Gentamicin	Aminoglycosides	Digestive decontamination	Prophylaxis	2 capsules	Per os	BID	14
			Meropenem	Carbapenems	Anti-infective prophylaxis	Prophylaxis	1g	IV	TID	5
			Nystatin	Polyene	Antifungal	Therapeutic	2 bottles	Per os	BID	20
			Piperacillin-Tazobactam	Penicillins	Anti-infective prophylaxis	Prophylaxis	4g	IV	BID	19
			Gentamicin	Aminoglycosides	Digestive decontamination	Prophylaxis	2 capsules	Per os	BID	7
Cohort 3	10	Cytarabine + Cytarabine/Daunorubicin + Hydroxycarbamide	-	-	-	-	-	-	-	-
	11	Cytarabine + Daunorubicin + Midostaurin	Amphotericin B	Macrolides	Oral care	Prophylaxis	10ml	Mouthwash	TID	28
	12	Cytarabine + Idarubicin	Amikacin	Aminoglycosides	Febrile neutropenia	Therapeutic	750mg	IV	QD	3
			Metronidazole	Nitroimidazoles	Febrile neutropenia	Therapeutic	1.5g	IV	QD	15
			Ceftriaxone	Cephalosporins	Febrile neutropenia	Therapeutic	2g	IV	QD	16
	13	Cytarabine + Idarubicin	Ceftazidime	Cephalosporins	Febrile neutropenia	Therapeutic	5g	IV	QD	24
			Metronidazole	Nitroimidazoles	Febrile neutropenia	Therapeutic	1g	IV	QD	24
	14	Cytarabine + Idarubicin	Vancomycin	Glycopeptides	Pneumonia	Therapeutic	2000mg	IV	QD	5
			Meropenem	Carbapenems	Pneumonia	Therapeutic	1g	IV	TID	18
			Vancomycin	Glycopeptides	*Clostridium difficile*	Therapeutic	125mg	Per os	QID	9
			Piperacillin-Tazobactam	Penicillins	Anti-infective prophylaxis	Prophylaxis	4g	IV	TID	1
	15	Cytarabine + Idarubicin + Hydroxycarbamide	Piperacillin-Tazobactam	Penicillins	Febrile neutropenia	Therapeutic	4g	IV	TID	19

			Amphotericin B	Macrolides	Mucositis	Therapeutic	10%	Per os	QID	13
			Meropenem	Carbapenems	Fever	Therapeutic	1g	IV	TID	4
Cohort 4	16	Cytarabine + Idarubicin	Spiramycin	Macrolides	Anti-infective prophylaxis	Prophylaxis	1.5 MIU	IV	BID	4
			Piperacillin-Tazobactam	Penicillins	Febrile neutropenia	Therapeutic	4mg	IV	TID	21
			Cefepime	Cephalosporins	Febrile neutropenia	Therapeutic	2g	IV	BID	1
			Amoxicillin	Penicillins	Septicemia *Escherichia coli*	Therapeutic	500mg	Per os	QM	4
			Daptomycin	Lipopeptides	Septicemia *Enterococcus faecium*	Therapeutic	600mg	IV	QD	6
	17	Cytarabine/Daunorubicin	Colistin	Polymyxins	Anti-infective prophylaxis	Prophylaxis	2 capsules	Per os	TID	19
			Vancomycin	Glycopeptides	Anti-infective prophylaxis	Prophylaxis	1g	IV	QD	1
			Piperacillin-Tazobactam	Penicillins	Anti-infective prophylaxis	Prophylaxis	4g	IV	QD	2
			Cefazolin	Cephalosporins	Bacteremia (*Staphylococcus epidermidis*)	Therapeutic	2g	IV	TID	5
			Clindamycin	Lincosamides	Bacteremia (*Staphylococcus epidermidis*)	Therapeutic	600mg	IV	TID	5
			Piperacillin-Tazobactam	Penicillins	Anti-infective prophylaxis	Prophylaxis	4g	IV	QID	5
			Amikacin	Aminoglycosides	Anti-infective prophylaxis	Prophylaxis	1200mg	IV	QD	1
			Meropenem	Carbapenems	Anti-infective prophylaxis	Prophylaxis	1g	IV	TID	1
			Vancomycin	Glycopeptides	Anti-infective prophylaxis	Prophylaxis	1g	IV	QD	1
	18	Cytarabine + Idarubicin + Midostaurin	Piperacillin-Tazobactam	Penicillins	Sepsis	Therapeutic	4g	IV	TID	15
			Cefepime	Cephalosporins	Sepsis	Therapeutic	2g	IV	BID	6
			Meropenem	Carbapenems	Fever	Therapeutic	1g	IV	TID	4
			Clindamycin	Lincosamides	Sepsis	Therapeutic	600mg	IV	TID	6
			Cefotaxime	Cephalosporins	Septic shock	Therapeutic	658mg	IV	QD	1
	19	Cytarabine + Idarubicin	Colistin	Polymyxins	Anti-infective prophylaxis	Prophylaxis	2g	Per os	TID	27
			Piperacillin-Tazobactam	Penicillins	Anti-infective prophylaxis	Prophylaxis	4g	IV	TID	16
			Amikacin	Aminoglycosides	Fever	Therapeutic	900mg	IV	QD	1
			Meropenem	Carbapenems	Fever	Therapeutic	1g	IV	TID	10

			Vancomycin	Glycopeptides	Fever	Therapeutic	2.5g	IV	QD	4
	20	Cytarabine + Idarubicin	Piperacillin-Tazobactam	Penicillins	Anti-infective prophylaxis	Prophylaxis	4g	IV	TID	5
			Amikacin	Aminoglycosides	Anti-infective prophylaxis	Prophylaxis	1250mg	IV	QD	1
			Meropenem	Carbapenems	Fever	Therapeutic	1g	IV	TID	10
			Vancomycin	Glycopeptides	Fever	Therapeutic	1250mg	IV	BID	2
			Colistin	Polymyxins	Anti-infective prophylaxis	Prophylaxis	2 capsules	Per os	BID	25
	21	Cytarabine + Idarubicin + Lomustine	Amphotericin B	Macrolides	Mucositis prophylaxis	Prophylaxis	5mL	Per os	TID	20
			Piperacillin-Tazobactam	Penicillins	Fever	Therapeutic	4g	IV	QID	28

IV: Intravenous, MIU: Million International Units, QD: Once daily, BID: Twice a day, TID: Three times a day, QID: Four times a day, QM: Every morning, PRN: As needed.

None of the antibiotics mentioned in this table were related to an adverse event

Supplementary Table 5: Antibiotic and chemotherapy received by each patient during consolidation cycle (between Day 19 and Day 44)

Cohort	Patient number	Consolidation cycle or other cycle	Antibiotic name	Antibiotic classification	Antibiotic indication	Prophylaxis/Therapeutic	Related to an AE ?	Antibiotic dosage	Route of administration	Frequency	Antibiotic duration (days)
Cohort 1	1	Cytarabine + Midostaurin	Daptomycin	Lipopeptides	Parotitis	Therapeutic	Yes	500mg	IV	QD	12
			Meropenem	Carbapenems	Parotitis	Therapeutic	Yes	1g	IV	TID	8
			Metronidazole	Nitroimidazoles	Parotitis	Therapeutic	Yes	500mg	IV	TID	7
			Cefepime	Cephalosporins	Parotitis	Therapeutic	Yes	2g	IV	TID	6
	2	Cytarabine + Idarubicin	None	NA	NA	NA	NA	NA	NA	NA	NA
	3	Cytarabine + Idarubicin + Methotrexate	None	NA	NA	NA	NA	NA	NA	NA	NA
Cohort 2	4	Cytarabine	None	NA	NA	NA	NA	NA	NA	NA	NA
	5	Azacitidine + Venetoclax	Piperacillin; Tazobactam	Penicillins	Febrile neutropenia	Therapeutic	Yes	4g	IV	TID	NA
			Amoxicillin; Clavulanate	Penicillins	Cellulitis	Therapeutic	Yes	1g	Per os	TID	12
			Ciprofloxacin	Fluoroquinolones	Cellulitis	Therapeutic	Yes	500mg	Per os	BID	12
			Amoxicillin; Clavulanate	Penicillins	Febrile neutropenia	Therapeutic	No	1g	IV	TID	9
	6	Cytarabine	Levofloxacin	Fluoroquinolones	Anti-infective prophylaxis	Prophylaxis	No	500mg	Per os	QD	9
	7	Cytarabine	Piperacillin; Tazobactam	Penicillins	Fever	Therapeutic	Yes	4g	IV	QID	17
			Daptomycin	Lipopeptides	Fever	Therapeutic	Yes	700 mg	IV	QD	0
	8	Cytarabine	Cefepime	Cephalosporins	Febrile neutropenia	Therapeutic	Yes	2g	IV	TID	NA
	9	Cytarabine	None	NA	NA	NA	NA	NA	NA	NA	NA
Cohort 3	10	Daunorubicin	Piperacillin; Tazobactam	Penicillins	Neutropenic colitis	Therapeutic	Yes	6g	IV	BID	4
			Vancomycin	Glycopeptides	Neutropenic colitis	Therapeutic	Yes	2,5g	IV	QD	1
			Gentamicin	Aminoglycosides	Neutropenic colitis	Therapeutic	Yes	400mg	IV	QD	2
			Cefotaxime	Cephalosporins	Neutropenic colitis	Therapeutic	Yes	1g	IV	TID	4
			Ceftriaxone	Cephalosporins	Neutropenic colitis	Therapeutic	Yes	1g	IV	QD	10
	11	Cytarabine + Midostaurin	Piperacillin; Tazobactam	Penicillins	Fever	Therapeutic	Yes	4g	IV	QID	4
	12	Cytarabine	Pristinamycin	Streptogramins	Panaritium	Therapeutic	Yes	1g	Per os	BID	12

	13	Cytarabine	None	NA	NA	NA	NA	NA	NA	NA	NA
	14	Cytarabine + Idarubicin	Cefuroxime axetil	Cephalosporins	Anti-infective prophylaxis	Prophylaxis	No	500mg	Per os	BID	6
			Ofloxacin	Fluoroquinolones	Anti-infective prophylaxis	Prophylaxis	No	200mg	Per os	BID	6
	15	Cytarabine	Cefepime	Cephalosporins	Febrile neutropenia	Therapeutic	Yes	2g	IV	TID	1
Cohort 4	16	Cytarabine	Cefepime	Cephalosporins	Septicemia *E. coli*	Therapeutic	Yes	2g	IV	QD	0
			Amoxicillin	Penicillins	Septicemia *E. coli*	Therapeutic	Yes	500mg	Per os	2 tablets 3 times a day	7
	17	Cytarabine	None	NA	NA	NA	NA	NA	NA	NA	NA
	18	None	None	NA	NA	NA	NA	NA	NA	NA	NA
	19	Cytarabine	Colistin	Polymyxins	Anti-infective prophylaxis	Prophylaxis	No	2 capsules	Per os	TID	7
	20	Unknown → Premature termination									
	21	Cytarabine	None	NA	NA	NA	NA	NA	NA	NA	NA

IV: Intravenous, QD: Once daily, QID: Four times a day, TID: Three times a day, BID: Twice a day, QM: Every morning, NA: Not applicable

Supplementary Table 6: Adverse Events (AEs) occurring after first administration of MaaT033

System Organ Class<br>Preferred Term	Cohort 1<br>N=3		Cohort 2<br>N=6		Cohort 3<br>N=6		Cohort 4<br>N=6		Total<br>N=21	
	Patients	n[1]	Patients	n[1]	Patients	n[1]	Patients	n[1]	Patients	n[1]
**Any Class, Any Term**	2 (66.7%)	12	5 (83.3%)	42	6 (100.0%)	54	6 (100.0%)	28	19 (90.5%)	136
**Gastrointestinal disorders**	2 (66.7%)	3	5 (83.3%)	18	5 (83.3%)	22	6 (100.0%)	17	18 (85.7%)	60
Abdominal distension	1 (33.3%)	1	3 (50.0%)	3	3 (50.0%)	3	2 (33.3%)	2	9 (42.9%)	9
Constipation	1 (33.3%)	1	3 (50.0%)	6	2 (33.3%)	3	3 (50.0%)	3	9 (42.9%)	13
Diarrhea	0 (0.0%)	0	1 (16.7%)	2	3 (50.0%)	3	3 (50.0%)	3	7 (33.3%)	8
Nausea	0 (0.0%)	0	3 (50.0%)	3	1 (16.7%)	1	3 (50.0%)	4	7 (33.3%)	8
Abdominal pain	0 (0.0%)	0	1 (16.7%)	1	3 (50.0%)	3	2 (33.3%)	2	6 (28.6%)	6
Gastroesophageal reflux disease	0 (0.0%)	0	1 (16.7%)	1	3 (50.0%)	3	1 (16.7%)	1	5 (23.8%)	5
Hemorrhoids	0 (0.0%)	0	1 (16.7%)	1	1 (16.7%)	1	1 (16.7%)	1	3 (14.3%)	3
Dyspepsia	0 (0.0%)	0	0 (0.0%)	0	1 (16.7%)	1	1 (16.7%)	1	2 (9.5%)	2
Vomiting	1 (33.3%)	1	0 (0.0%)	0	1 (16.7%)	1	0 (0.0%)	0	2 (9.5%)	2
Abdominal rigidity	0 (0.0%)	0	0 (0.0%)	0	1 (16.7%)	1	0 (0.0%)	0	1 (4.8%)	1
Aphthous ulcer	0 (0.0%)	0	1 (16.7%)	1	0 (0.0%)	0	0 (0.0%)	0	1 (4.8%)	1
Eructation	0 (0.0%)	0	0 (0.0%)	0	1 (16.7%)	1	0 (0.0%)	0	1 (4.8%)	1
Neutropenic colitis	0 (0.0%)	0	0 (0.0%)	0	1 (16.7%)	1	0 (0.0%)	0	1 (4.8%)	1
**Metabolism and nutrition disorders**	1 (33.3%)	2	4 (66.7%)	4	2 (33.3%)	5	2 (33.3%)	2	9 (42.9%)	13
Decreased appetite	0 (0.0%)	0	1 (16.7%)	1	0 (0.0%)	0	1 (16.7%)	1	2 (9.5%)	2
Hypercholesterolemia	1 (33.3%)	1	0 (0.0%)	0	1 (16.7%)	1	0 (0.0%)	0	2 (9.5%)	2
Hypertriglyceridemia	1 (33.3%)	1	0 (0.0%)	0	1 (16.7%)	1	0 (0.0%)	0	2 (9.5%)	2
Hypokalemia	0 (0.0%)	0	1 (16.7%)	1	1 (16.7%)	1	0 (0.0%)	0	2 (9.5%)	2
Cell death	0 (0.0%)	0	0 (0.0%)	0	0 (0.0%)	0	1 (16.7%)	1	1 (4.8%)	1
Hyperkaliemia	0 (0.0%)	0	1 (16.7%)	1	0 (0.0%)	0	0 (0.0%)	0	1 (4.8%)	1
Hypocalcemia	0 (0.0%)	0	0 (0.0%)	0	1 (16.7%)	2	0 (0.0%)	0	1 (4.8%)	2
Vitamin K deficiency	0 (0.0%)	0	1 (16.7%)	1	0 (0.0%)	0	0 (0.0%)	0	1 (4.8%)	1
**Blood and lymphatic system disorders**	1 (33.3%)	1	3 (50.0%)	6	3 (50.0%)	9	1 (16.7%)	2	8 (38.1%)	18
Thrombocytopenia	1 (33.3%)	1	3 (50.0%)	3	3 (50.0%)	5	1 (16.7%)	1	8 (38.1%)	10
Anemia	0 (0.0%)	0	1 (16.7%)	1	1 (16.7%)	2	1 (16.7%)	1	3 (14.3%)	4
Febrile neutropenia	0 (0.0%)	0	2 (33.3%)	2	1 (16.7%)	1	0 (0.0%)	0	3 (14.3%)	3
Neutropenia	0 (0.0%)	0	0 (0.0%)	0	1 (16.7%)	1	0 (0.0%)	0	1 (4.8%)	1
**General disorders and administration site conditions**	2 (66.7%)	2	2 (33.3%)	4	2 (33.3%)	2	1 (16.7%)	1	7 (33.3%)	9
Pyrexia	1 (33.3%)	1	1 (16.7%)	2	2 (33.3%)	2	0 (0.0%)	0	4 (19.0%)	5
Mucosal inflammation	1 (33.3%)	1	1 (16.7%)	1	0 (0.0%)	0	0 (0.0%)	0	2 (9.5%)	2
Chest pain	0 (0.0%)	0	0 (0.0%)	0	0 (0.0%)	0	1 (16.7%)	1	1 (4.8%)	1
Oedema	0 (0.0%)	0	1 (16.7%)	1	0 (0.0%)	0	0 (0.0%)	0	1 (4.8%)	1
**Infections and infestations**	0 (0.0%)	0	2 (33.3%)	2	1 (16.7%)	1	2 (33.3%)	3	5 (23.8%)	6
Cellulitis	0 (0.0%)	0	1 (16.7%)	1	0 (0.0%)	0	0 (0.0%)	0	1 (4.8%)	1
Diarrhea-infectious	0 (0.0%)	0	0 (0.0%)	0	0 (0.0%)	0	1 (16.7%)	1	1 (4.8%)	1
Escherichia sepsis	0 (0.0%)	0	0 (0.0%)	0	0 (0.0%)	0	1 (16.7%)	1	1 (4.8%)	1
Paronychia	0 (0.0%)	0	0 (0.0%)	0	1 (16.7%)	1	0 (0.0%)	0	1 (4.8%)	1
Pseudomonas infection	0 (0.0%)	0	0 (0.0%)	0	0 (0.0%)	0	1 (16.7%)	1	1 (4.8%)	1
Skin infection	0 (0.0%)	0	1 (16.7%)	1	0 (0.0%)	0	0 (0.0%)	0	1 (4.8%)	1
**Skin and subcutaneous tissue disorders**	1 (33.3%)	1	2 (33.3%)	3	2 (33.3%)	3	0 (0.0%)	0	5 (23.8%)	7
Rash	0 (0.0%)	0	1 (16.7%)	2	1 (16.7%)	1	0 (0.0%)	0	2 (9.5%)	3
Erythema	0 (0.0%)	0	0 (0.0%)	0	1 (16.7%)	1	0 (0.0%)	0	1 (4.8%)	1
Petechiae	0 (0.0%)	0	0 (0.0%)	0	1 (16.7%)	1	0 (0.0%)	0	1 (4.8%)	1
Psoriasis	1 (33.3%)	1	0 (0.0%)	0	0 (0.0%)	0	0 (0.0%)	0	1 (4.8%)	1
Toxic skin eruption	0 (0.0%)	0	1 (16.7%)	1	0 (0.0%)	0	0 (0.0%)	0	1 (4.8%)	1
**Vascular disorders**	0 (0.0%)	0	0 (0.0%)	0	2 (33.3%)	2	2 (33.3%)	2	4 (19.0%)	4
Deep vein thrombosis	0 (0.0%)	0	0 (0.0%)	0	0 (0.0%)	0	2 (33.3%)	2	2 (9.5%)	2
Haemorrhage	0 (0.0%)	0	0 (0.0%)	0	1 (16.7%)	1	0 (0.0%)	0	1 (4.8%)	1
Orthostatic hypotension	0 (0.0%)	0	0 (0.0%)	0	1 (16.7%)	1	0 (0.0%)	0	1 (4.8%)	1
**Eye disorders**	1 (33.3%)	1	2 (33.3%)	2	0 (0.0%)	0	0 (0.0%)	0	3 (14.3%)	3
Dry eye	1 (33.3%)	1	1 (16.7%)	1	0 (0.0%)	0	0 (0.0%)	0	2 (9.5%)	2
Keratitis	0 (0.0%)	0	1 (16.7%)	1	0 (0.0%)	0	0 (0.0%)	0	1 (4.8%)	1
**Nervous system disorders**	1 (33.3%)	1	0 (0.0%)	0	1 (16.7%)	3	0 (0.0%)	0	2 (9.5%)	4
Dizziness	0 (0.0%)	0	0 (0.0%)	0	1 (16.7%)	2	0 (0.0%)	0	1 (4.8%)	2
Headache	1 (33.3%)	1	0 (0.0%)	0	0 (0.0%)	0	0 (0.0%)	0	1 (4.8%)	1

System Organ Class<br>Preferred Term	Cohort 1<br>N=3		Cohort 2<br>N=6		Cohort 3<br>N=6		Cohort 4<br>N=6		Total<br>N=21	
	Patients	n¹	Patients	n¹	Patients	n¹	Patients	n¹	Patients	n¹
Resting tremor	0 (0.0%)	0	0 (0.0%)	0	1 (16.7%)	1	0 (0.0%)	0	1 (4.8%)	1
**Psychiatric disorders**	1 (33.3%)	1	1 (16.7%)	1	0 (0.0%)	0	0 (0.0%)	0	2 (9.5%)	2
Anxiety	1 (33.3%)	1	0 (0.0%)	0	0 (0.0%)	0	0 (0.0%)	0	1 (4.8%)	1
Depression	0 (0.0%)	0	1 (16.7%)	1	0 (0.0%)	0	0 (0.0%)	0	1 (4.8%)	1
**Cardiac disorders**	0 (0.0%)	0	0 (0.0%)	0	1 (16.7%)	1	0 (0.0%)	0	1 (4.8%)	1
Tachycardia	0 (0.0%)	0	0 (0.0%)	0	1 (16.7%)	1	0 (0.0%)	0	1 (4.8%)	1
**Injury, poisoning and procedural complications**	0 (0.0%)	0	0 (0.0%)	0	1 (16.7%)	1	0 (0.0%)	0	1 (4.8%)	1
Fall	0 (0.0%)	0	0 (0.0%)	0	1 (16.7%)	1	0 (0.0%)	0	1 (4.8%)	1
**Investigations**	0 (0.0%)	0	0 (0.0%)	0	1 (16.7%)	2	0 (0.0%)	0	1 (4.8%)	2
Blood phosphorus decreased	0 (0.0%)	0	0 (0.0%)	0	1 (16.7%)	2	0 (0.0%)	0	1 (4.8%)	2
**Musculoskeletal and connective tissue disorders**	0 (0.0%)	0	0 (0.0%)	0	0 (0.0%)	0	1 (16.7%)	1	1 (4.8%)	1
Myalgia	0 (0.0%)	0	0 (0.0%)	0	0 (0.0%)	0	1 (16.7%)	1	1 (4.8%)	1

SOC: System organ class, N: Number of patients, N (%): Number and percentage of patients with at least one event, n¹: Number of events

Supplementary Table 7: Gastrointestinal adverse events reported during MaaT033 treatment phase (Day 1 to Day 19) and during consolidation cycle (from Day 19 to Day 44)

	System Organ Class<br>Preferred Term	Cohort 1<br>N=3		Cohort 2<br>N=6		Cohort 3<br>N=6		Cohort 4<br>N=6		Total<br>N=21	
		Patients	n[1]	Patients	n[1]	Patients	n[1]	Patients	n[1]	Patients	n[1]
**From Day 1 to Day 19**	**Gastrointestinal disorders**	2 (66.7%)	3	4 (66.7%)	8	5 (83.3%)	10	6 (100.0%)	11	17 (81.0%)	32
	Abdominal distension	1 (33.3%)	1	2 (33.3%)	2	2 (33.3%)	2	1 (16.7%)	1	6 (28.6%)	6
	Constipation	1 (33.3%)	1	2 (33.3%)	2	1 (16.7%)	1	2 (33.3%)	2	6 (28.6%)	6
	Abdominal pain	0 (0.0%)	0	1 (16.7%)	1	3 (50.0%)	3	1 (16.7%)	1	5 (23.8%)	5
	Diarrhoea	0 (0.0%)	0	1 (16.7%)	1	1 (16.7%)	1	2 (33.3%)	2	4 (19.0%)	4
	Gastroesophageal reflux disease	0 (0.0%)	0	1 (16.7%)	1	1 (16.7%)	1	1 (16.7%)	1	3 (14.3%)	3
	Dyspepsia	0 (0.0%)	0	0 (0.0%)	0	1 (16.7%)	1	1 (16.7%)	1	2 (9.5%)	2
	Haemorrhoids	0 (0.0%)	0	1 (16.7%)	1	0 (0.0%)	0	1 (16.7%)	1	2 (9.5%)	2
	Nausea	0 (0.0%)	0	0 (0.0%)	0	0 (0.0%)	0	2 (33.3%)	2	2 (9.5%)	2
	Eructation	0 (0.0%)	0	0 (0.0%)	0	1 (16.7%)	1	0 (0.0%)	0	1 (4.8%)	1
	Vomiting	1 (33.3%)	1	0 (0.0%)	0	0 (0.0%)	0	0 (0.0%)	0	1 (4.8%)	1
**From Day 19 to Day 44**	**Gastrointestinal disorders**	0 (0.0%)	0	5 (83.3%)	10	4 (66.7%)	12	3 (50.0%)	6	12 (57.1%)	28
	Constipation	0 (0.0%)	0	3 (50.0%)	4	2 (33.3%)	2	1 (16.7%)	1	6 (28.6%)	7
	Nausea	0 (0.0%)	0	3 (50.0%)	3	1 (16.7%)	1	2 (33.3%)	2	6 (28.6%)	6
	Diarrhoea	0 (0.0%)	0	1 (16.7%)	1	2 (33.3%)	2	1 (16.7%)	1	4 (19.0%)	4
	Abdominal distension	0 (0.0%)	0	1 (16.7%)	1	1 (16.7%)	1	1 (16.7%)	1	3 (14.3%)	3
	Gastroesophageal reflux disease	0 (0.0%)	0	0 (0.0%)	0	2 (33.3%)	2	0 (0.0%)	0	2 (9.5%)	2
	Abdominal pain	0 (0.0%)	0	0 (0.0%)	0	0 (0.0%)	0	1 (16.7%)	1	1 (4.8%)	1
	Abdominal rigidity	0 (0.0%)	0	0 (0.0%)	0	1 (16.7%)	1	0 (0.0%)	0	1 (4.8%)	1
	Aphthous ulcer	0 (0.0%)	0	1 (16.7%)	1	0 (0.0%)	0	0 (0.0%)	0	1 (4.8%)	1
	Haemorrhoids	0 (0.0%)	0	0 (0.0%)	0	1 (16.7%)	1	0 (0.0%)	0	1 (4.8%)	1
	Neutropenic colitis	0 (0.0%)	0	0 (0.0%)	0	1 (16.7%)	1	0 (0.0%)	0	1 (4.8%)	1
	Vomiting	0 (0.0%)	0	0 (0.0%)	0	1 (16.7%)	1	0 (0.0%)	0	1 (4.8%)	1

N: number of patients per cohort, n (%): Number and percentage of patients with at least one event, n[1] : Number of events

Supplementary Table 8: Listing of Serious Adverse Events (SAEs) reported during the CIMON study

SOC	PT	Cohort 1 (N=3)<br><br>n (%)	Cohort 2 (N=6)<br><br>n (%)	Cohort 3 (N=6)<br><br>n (%)	Cohort 4 (N=6)<br><br>n (%)	Total (N=21)<br>n (%)
Blood and lymphatic system disorders	Febrile neutropenia	0	1 (17%)	0	0	1 (5%)
Gastrointestinal disorders	Neutropenic colitis	0	0	1 (17%)	0	1 (5%)
Infections and infestations	Diarrhoea infectious	0	0	0	1 (17%)	1 (5%)
Metabolism and nutrition disorders	Hyperkalaemia	0	1 (17%)	0	0	1 (5%)

SOC: System organ class, PT: Preferred term, n (%): Number and percentage of patients with at least one SAE

Supplementary Table 9: Clinical outcomes of CIMON study patients

	Patient number	Disease status at last follow-up	AlloHCT Yes/ No	GvHD Yes / No	Survival status Alive Yes /No	Cause of death	Follow-up duration (days)
Cohort 1	1	Remission	No	No	No	*P. aeruginosa* pneumonia toto-R	87
	2	Remission	Yes	Grade II skin aGvHD Grade I GI aGvHD	Yes		1063
	3	Remission	No	No	No	Unknown	175
Cohort 2	4	Remission	Yes	Grade III liver aGvHD	No	Multiple organ failure	118
	5	Relapse (cytologic)	No	No	No	Relapse	118
	6	Remission	Yes	Grade I skin aGvHD	Yes		968
	7	Relapse (cytologic)	No	No	No	Relapse	96
	8	Remission	Yes	Grade I skin aGvHD	Yes		804
	9	Relapse (molecular)	Yes	No	Yes		964
Cohort 3	10	Remission	Yes	No	No	Pneumopathy	706
	11	Relapse (cytologic)	No	No	No	Cerebral hemorrhage	142
	12	Remission	No	No	Yes		829
	13	Relapse (cytologic)	No	No	No	Relapse	723
	14	Remission	Yes	No	No	Fungal infection	652
	15	Remission	No	No	Yes		773
Cohort 4	16	Remission	yes	Grade II skin aGvHD	Yes		819
	17	Relapse (cytologic)	No	No	No	Relapse	246
	18	Remission	Yes	Grade II skin and GI aGvHD	Yes		735
	19	Remission	Yes	mild cGvHD skin / liver / GI	No	Multiple organ failure	462
	20	Relapse (cytologic)	Yes	No	Yes		735
	21	Remission	Yes	No	Yes		256

Follow-up duration calculated from patient's inclusion to patient's last follow-up or death

AlloHCT: allogeneic cell transplantation, aGvHD: acute graft-versus-host disease, cGvHD: chronic graft-versus-host disease, GI: gastrointestinal

Supplementary Figures

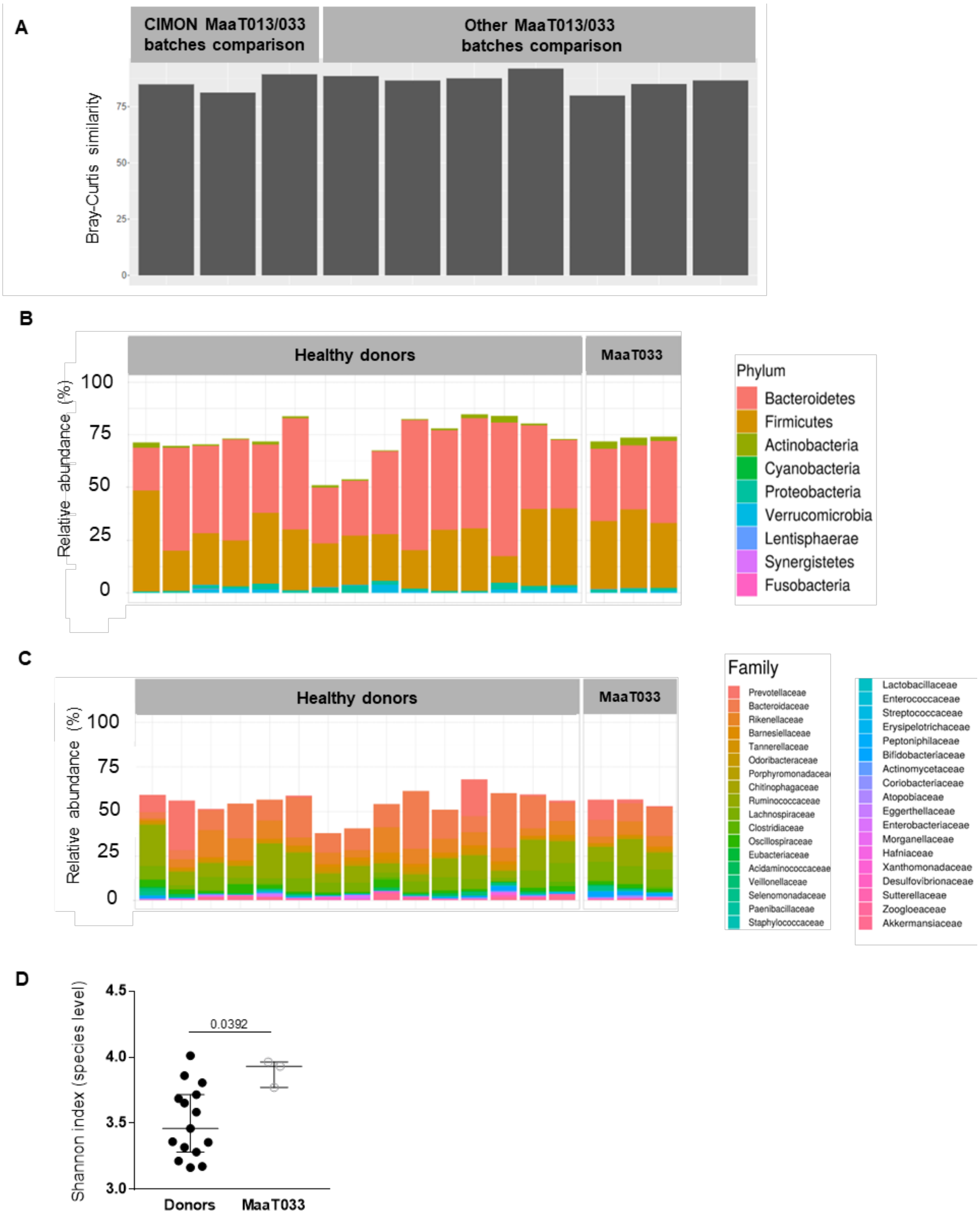

Supplementary Figure 1. Metagenomic characterization of MaaT033 batches using shotgun sequencing

(A) Bray-Curtis similarities at species level between MaaT013 (enema formulation) and MaaT033 (oral lyophilized formulation) products. The bars represent the similarity between 10 pairs of capsules and enema products made from the same donor pools (1 donor pool per pair of products). The first 3 bars correspond to the MaaT033 products used in the CIMON study.

(B) Stacked barplot of phyla relative abundances of the 3 MaaT033 batches used in the CIMON study and the 15 single healthy donors used for their manufacturing.

(C) Stacked barplot of family relative abundances of the 3 MaaT033 batches used in the CIMON study and the 15 single healthy donors used for their manufacturing.

(D) Shannon index at species level measured in the 15 single healthy donors and the 3 MaaT033 batches. Medians with interquartile ranges are provided. Statistical significance evaluated using unpaired Mann-Whitney test.

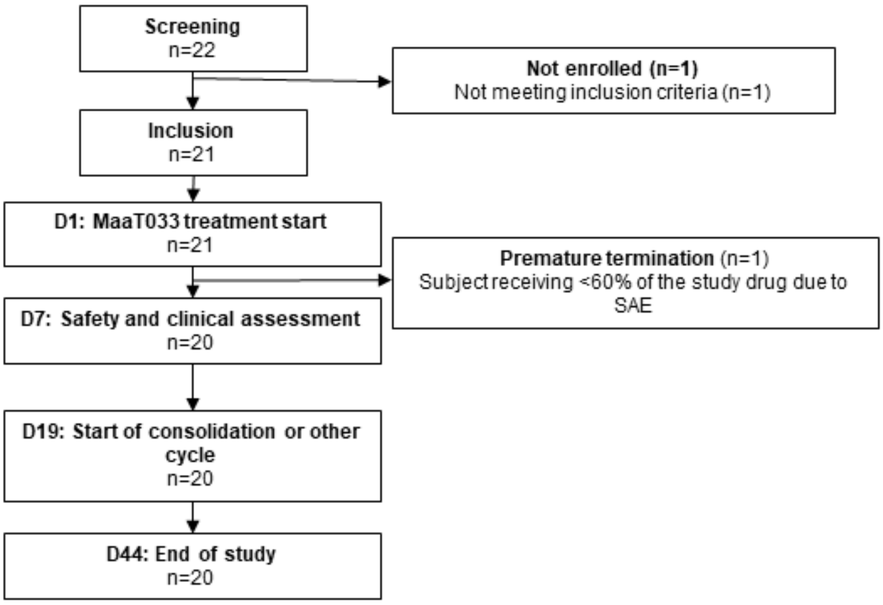

Supplementary Figure 2: **CONSORT diagram**

D: day; n: number of patients; SAE: serious adverse event

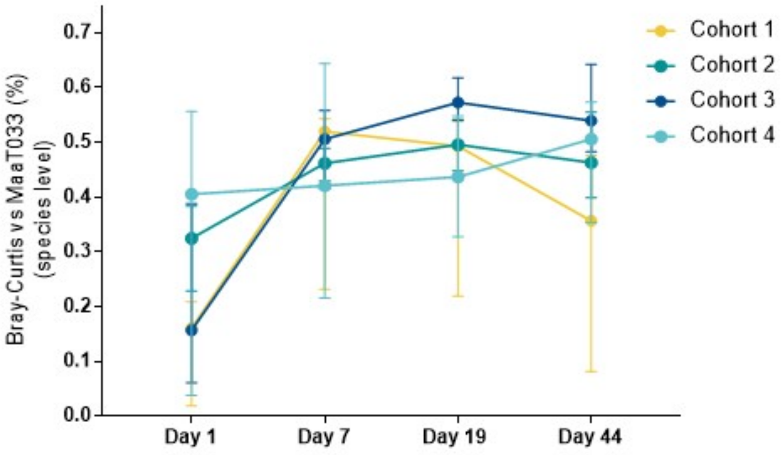

Supplementary Figure 3: Bray-Curtis similarity index at species level from Day 1 to Day 44 for all cohorts of patients (n=21).

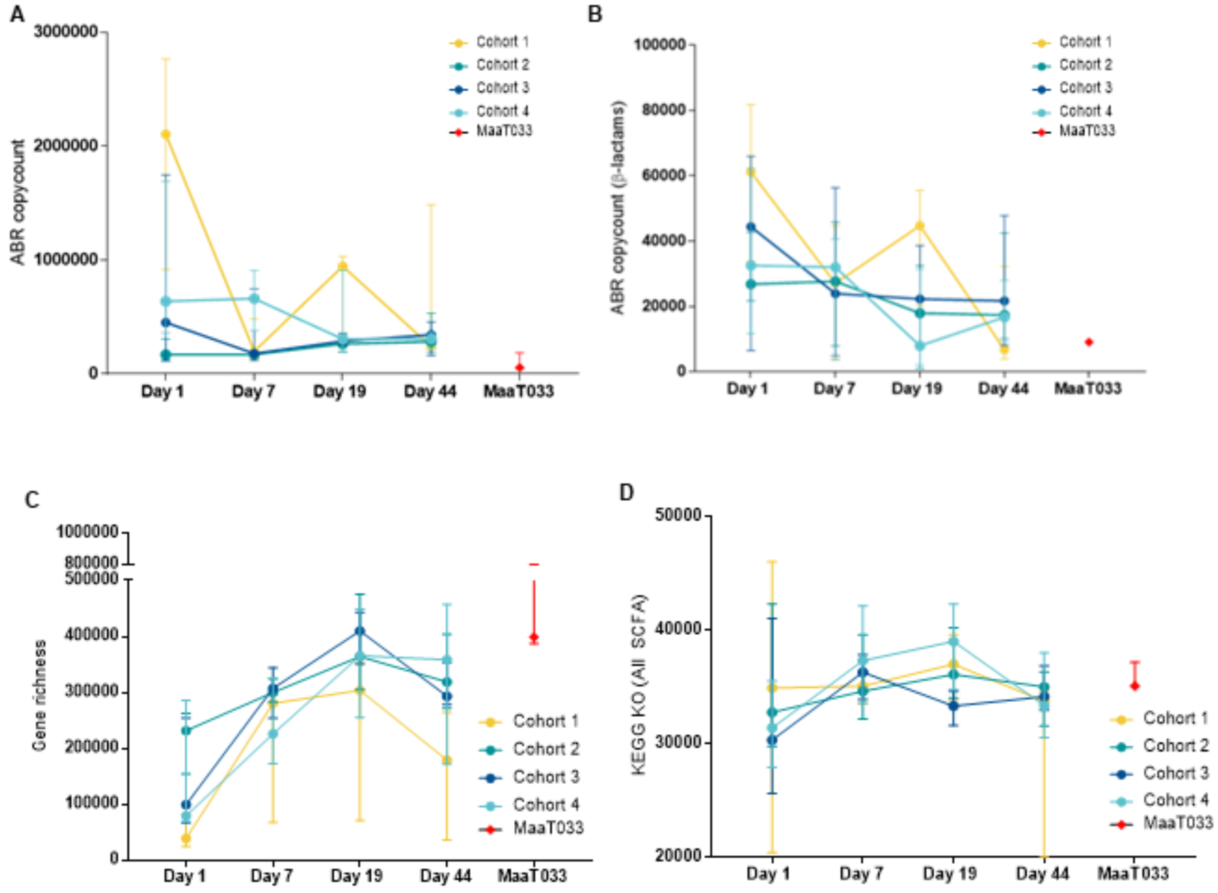

Supplementary Figure 4: Metagenomic sequencing with analysis of antibiotic resistance genes, gene richness, and number of genes associated with SCFAs from Day 1 to Day 44 for all cohorts of patients (n=21).

Number of reads mapped against total antibiotic (A) and β-lactams (B) resistance genes identified through metagenomic sequencing from Day 1 to Day 44 for all cohorts of patients.

ABR is for antibiotic resistance genes.Two MaaT033 batch samples and some patients' samples are not included in the results of antibiotic resistance gene (β-lactams) analyses since the number of sequences corresponding to antibiotic resistance genes was insufficient to perform this analysis, and have thus been eliminated.

(C) Gene richness
(D) Number of genes associated with KEGG Orthology (KO) of short-chain fatty acid (SCFA) metabolism.

Medians with interquartile ranges are provided.

A

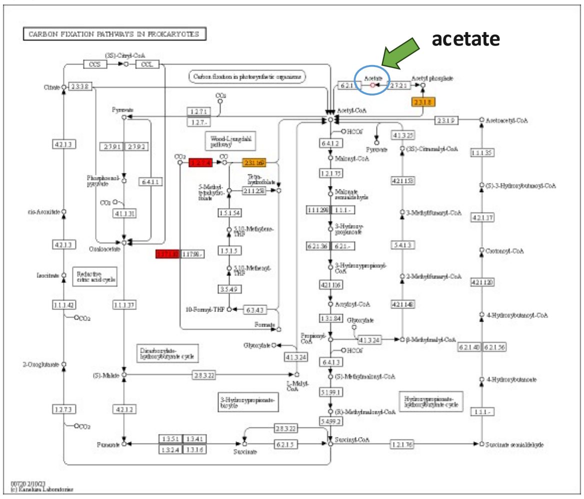

B

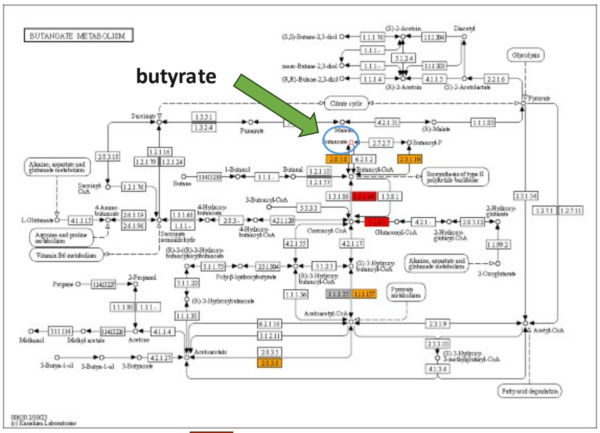

Positive correlation (*)

Positive correlation (trend)

Negative correlation (*)

C Acetate list of KOs and related EC numbers

KEGG KO	Corresponding gene codes	Description	EC number
K00197	cdhE, acsC	acetyl-CoA decarbonylase/synthase, CODH/ACS complex subunit gamma	EC:2.1.1.245, EC 2.3.1.169
K00198	cooS, acsA	anaerobic carbon-monoxide dehydrogenase catalytic subunit	EC:1.2.7.4
K15022	fdhB	formate dehydrogenase (NADP+) beta subunit	EC:1.17.1.10
K15024	K15024	putative phosphotransacetylase	EC:2.3.1.8

D Butyrate list of KOs and related EC numbers

KEGG KO	Corresponding gene codes	Description	EC number
K01034	atoD	acetate CoA/acetoacetate CoA - transferase alpha subunit	EC:2.8.3.8, EC: 2.8.3.9
K00634	ptb	phosphate butyryltransferase	EC:2.3.1.19
K01615	gcdA	glutaconyl-CoA decarboxylase subunit alpha	EC:7.2.4.5
K00209	fabV, ter	enoyl-acyl-carrier protein reductase / trans-2-enoyl-CoA reductase (NAD+)	EC:1.3.1.9, EC:1.3.1.44
K01035	atoA	acetate CoA/acetoacetate CoA - transferase beta subunit	EC:2.8.3.8, EC:2.8.3.9
K00074	paaH, hbd, fadB, mmgB	3-hydroxybutyryl-CoA dehydrogenase	EC:1.1.1.157
K07516	fadN	3-hydroxyacyl-CoA dehydrogenase	EC:1.1.1.35

Supplementary Figure 5: Acetate (A) and butyrate (B) pathway gene orthologs (KEGG orthology (KOs) mapped to the related acetate and butanoate (= butyrate) metabolism pathway and corresponding KEGG descriptions (C and D).

Positive correlation between KO abundances (from metagenomic analysis) and related fecal measurements of acetate and butyrate are mentioned in red when significant, or orange if only a trend was observed. Negative correlation is highlighted in grey.

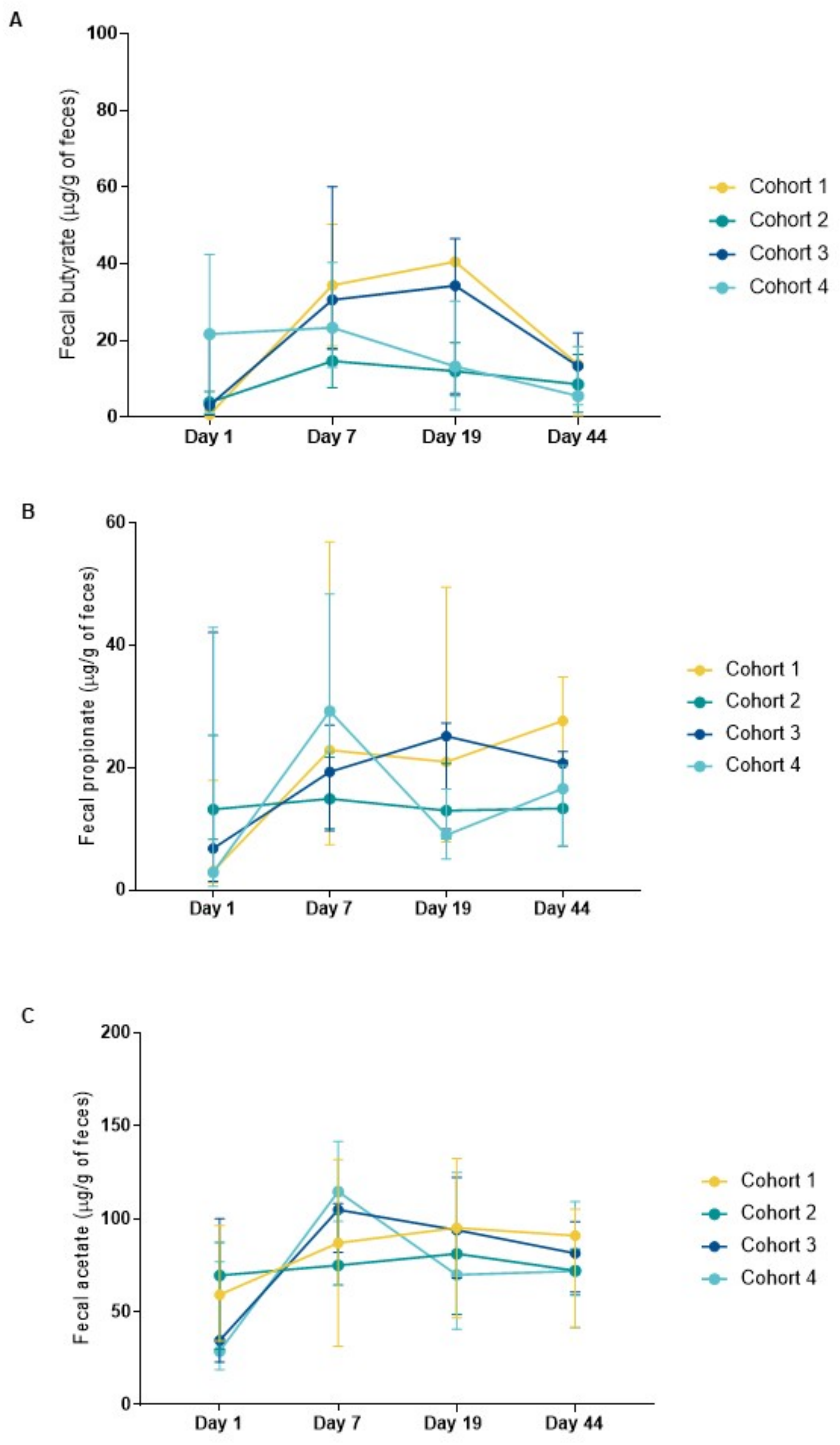

Supplementary Figure 6: Fecal short-chain fatty acids from Day 1 to Day 44 for all cohorts of patients (n=21).

(A) Butyrate
(B) Propionate
(C) Acetate

Medians with interquartile ranges are provided.
