# Supplementary material for: Gut microbiota restoration with oral pooled fecal microbiotherapy after intensive chemotherapy: the phase 1b CIMON trial
Source: Blood Adv. 2025 Apr 10;9(15):3739–49. doi: 10.1182/bloodadvances.2024015571 (PMC12305571; doi:10.1182/bloodadvances.2024015571)
Supplement: Supplemental Methods and Appendices [file BLOODA_ADV-2024-015571-mmc2.pdf]

# Data Safety Monitoring Board (DSMB)
# Charter

Safety phase I evaluation of MaaT033, a lyophilized full-ecosystem intestinal microbiota delayed release Capsule, In patients with acute MyelOid Leukemia or High-Risk Myelodysplastic Syndrome after iNtensive chemotherapy

**Confidential**

| | |
|---|---|
| **Name of Sponsor:** | MaaT Pharma |
| **EudraCT number:** | 2019-004253-89 |
| **Protocol Number:** | MPOH05 |

## Version History

| Version | Date | Author | Change description |
|---|---|---|---|
| 1.0 | 16 Mar 2020 | Floriane FAURE | Initial version |
| 2.0 | 04 Jan 2022 | Floriane FAURE | Alignment with protocol regarding the responsibilities of CRC and SC. |

All documentation of **MaaT Pharma** is PROPRIETARY information and may not be forwarded to third parties without prior written consent from **MaaT Pharma.** Reproductions, either in part or in whole, may not be published or copied in any manner, without the explicit written consent of **MaaT Pharma.**

## TABLE OF CONTENTS

1 PURPOSE OF DATA SAFETY MONITORING BOARD CHARTER .......... 3

2 STUDY OVERVIEW .......... 3

3 SAFETY DATA .......... 4

4 DSMB MEMBERSHIP .......... 6

5 COMPOSITION OF THE DSMB .......... 6

6 INDEPENDENCE OF THE DSMB .......... 7

7 RESPONSIBILITIES OF THE DSMB .......... 7

8 DSMB CHAIR RESPONSIBILITIES .......... 8

9 MEETINGS OF THE DSMB .......... 9

9.1 Data transfer and meeting format .......... 9
9.2 Mandatory meetings .......... 9
9.3 Minutes .......... 9

10 RECOMMENDATIONS .......... 10

11 STUDY REVIEW CRITERIA/STOPPING RULES AND GUIDELINES .......... 10

11.1 Individual Stopping Criteria .......... 10
11.2 Safety monitoring .......... 10
11.3 Study Stopping Criteria .......... 10

11 AMENDMENTS TO THE CHARTER .......... 10

12 COMPLETION OF DSMB ACTIVITIES .......... 11

13 CONFIDENTIALITY .......... 11

Appendix A: DSMB Member Signature Page .......... 12

Appendix B: Conflict of Interest Statement .......... 13

All documentation of **MaaT Pharma** is PROPRIETARY information and may not be forwarded to third parties without prior written consent from **MaaT Pharma.** Reproductions, either in part or in whole, may not be published or copied in any manner, without the explicit written consent of **MaaT Pharma.**

# 1 PURPOSE OF DATA SAFETY MONITORING BOARD CHARTER

MaaT Pharma (Hereinafter the "Sponsor") decided to set up a Data Safety Monitoring Board (herein referred to as the "DSMB") related to conduction of the Clinical trial (indifferently hereinafter "The trial or The Study") CIMON STUDY titled: "Safety phase I evaluation of MaaT033, a lyophilized full-ecosystem intestinal microbiota delayed release Capsule, In patients with acute MyelOid Leukemia after iNtensive chemotherapy" to assess the safety of the trial and provide recommendations to the sponsor.

The purpose of this charter is to define the roles and responsibilities of the DSMB as well as the operating rules as part of the trial, delineate qualifications of the membership, describe the purpose and timing of meetings, provide the procedures for ensuring confidentiality and proper communication, and outline the content of the reports. This charter will serve as the Standard Operating Procedure (SOP) for the DSMB. The DSMB will be independent of the sponsor, regulatory agencies and investigators.

This charter will be approved by its DSMB members.

# 2 STUDY OVERVIEW

- Trial name: CIMON STUDY
- Trial sponsor: MaaT Pharma
- Trial design:

This study is an open-label, single-arm phase 1 study to evaluate the safety of MaaT033 in a step-up dosing design from 2 capsules 7 days apart to 9 capsules per day for 7 days.

The study has an adapted "6 dose titration" escalation design to allow for assessment of tolerability (research of the Maximal Tolerated Dose) and of activity (research of Minimal Active Dose):

- ❖ **1st level of dose (cohort1)**: - 3 Patients will be treated with one capsule/day on day 1 and D7. The Safety Committee (SC) will evaluate occurrence of Limiting Toxicity after the 3 patients will have completed V3. If no patient experiments a DLT, the following cohort will use dose level 2 i.e. 1capsule/Day for 7 days. If one or more patient experiment a DLT, the SC might consider study stop recommendation or cohort continuation. The purpose of this cohort is to assess feasibility of the whole procedure and check any unexpected tolerability issue that would prohibit continuation of the experimentation. As the dose schedule is not considered as sufficient enough to allow for any effective activity, it will not be considered for the choice of final dose.

All documentation of **MaaT Pharma** is PROPRIETARY information and may not be forwarded to third parties without prior written consent from **MaaT Pharma.** Reproductions, either in part or in whole, may not be published or copied in any manner, without the explicit written consent of **MaaT Pharma.**

- **2nd Level of dose (cohort2)**: 6 patients will be treated with 1 capsule/day for 7 days. The Safety Committee (SC) will evaluate occurrence of Limiting Toxicity after the 6 patients will have completed V3. If no or 1 patient experiments a DLT, the following cohort will use dose level 3 i.e. 3 capsules/day for 7 days. If two or more patients out of 6 experiment a DLT, the study will be stopped.
- **3rd level of dose (cohort3):** 6 patients will be treated with 3 capsules/day for 7 days. The Safety Committee (SC) will evaluate occurrence of Limiting Toxicity after the 6 patients will have completed V3. If no or 1 patient experiments a DLT, the following cohort will use dose level 4 i.e. 3 capsules/D for 14 days and dose level 5 i.e. 9 capsules/day for 7 days. If two or more patients out of 6 experiment a DLT, the study will be stopped.
- **4th level of dose (cohort4):** 6 patients will be treated with 3 capsules/day for 14 days. The Safety Committee (SC) will evaluate occurrence of Limiting Toxicity after the 6 patients will have completed V3. If no or 1 patient experiments a DLT, the following cohort will use dose level 5 i.e. 9 capsules/day for 7 days. If two or more patients out of 6 experiment a DLT, the study will be stopped. Of note, if 2 or more DLT are reported during the 7 first days of treatment, then the SC will re-assess cohort3 evaluation.
- **5th level of dose (cohort5):** 6 patients will be treated with 9 capsules/day for 7 days. The Safety Committee (SC) will evaluate occurrence of Limiting Toxicity after the 6 patients will have completed V3. If two patients or more experiment a DLT, the study will be stopped and MTD will be considered as dose level 4. If one patient or less experiments a DLT, the MTD will be considered dose level 5.

- Phase: I
- Number of subjects to be included: 27 subjects will be included for the whole study.

In details, 3 subjects will be included in cohort1, and 6 subjects will be included in each other cohorts.

- Number of sites: 4

<u>European Coordinating Investigator:</u>

Pr Christian RECHER

The study has the following phases:

All documentation of **MaaT Pharma** is PROPRIETARY information and may not be forwarded to third parties without prior written consent from **MaaT Pharma.** Reproductions, either in part or in whole, may not be published or copied in any manner, without the explicit written consent of **MaaT Pharma.**

Patient pre-screening phase - Patients diagnosed with hematologic malignancy with will be asked if they are willing to participate in this study by the investigating physician and will sign the informed consent form before any study examination. Only patients with neutropenia recovery will be finally included in the study. Neutropenia recovery is defined as a level of Absolute Neutrophil Count (ANC) $\geq 0.5*10^9$ cells/L, over 2 following measures.

Treatment phase - The treatment will be started within 2 days after patient's inclusion. Clinical evaluation will be performed at baseline, at the interim visit (day7), before new cycle of chemotherapy start if any (day19 if not) and at the end of new cycle chemotherapy if any (day44 if not). A step-up process will be applied between 5 different cohorts of patients and will depend on patient's tolerance of the treatment.

Follow-up phase: Patient's overall status will be evaluated at D44 post inclusion (V4).

AE/SAE collection will be performed from time of informed consent to V4.

Under the term "DSMB", the protocol makes the distinction between the safety committee and the cohort review committee.

- The safety committee is in charge of:
  - the evaluation of the tolerability of the study for all patients
  - the evaluation of the relation of AEs to the drug treatment in a final review. The committee will then provide a conclusive decision on MaaT033 safety.

  Five members should attend to review the global safety at the end of the study, during the data review meeting and the statistical analysis.
- The Cohort Review Committee is a sub-category of the safety committee that reviews doses and makes decision regarding cohort escalation and protocol changes due to safety concerns. Once the MTD or MED is determined, or once the highest dose has been reached without declaring an MTD, this committee will select the recommended dose to be selected for the phase II study. The last meeting for this committee is at the end of cohort 5. The members are the same as the safety committee however the meeting can be held if at least 3 members are attending.

## 3 SAFETY DATA

The safety and tolerability of MaaT033 will be evaluated during the study with the collection of all AEs and SAEs (frequency, grade, relationship) from V1 to V4.

The events of interest during MaaT033 treatment are:

All documentation of **MaaT Pharma** is PROPRIETARY information and may not be forwarded to third parties without prior written consent from **MaaT Pharma.** Reproductions, either in part or in whole, may not be published or copied in any manner, without the explicit written consent of **MaaT Pharma.**

- Infections and infestations,

- GI disorders,

- Vital signs will also be monitored at each visit. Vital signs, blood pressure (SBP and DBP) and heart rate.

# 4 DSMB MEMBERSHIP

- DSMB members will sign confidentiality agreements covering DSMB activities.
- Remuneration will be provided by MaaT Pharma in accordance with standard procedures of the DSMB.
- The Committee will be composed of at least *3* members (inclusive of the DSMB Chair) for cohort review meetings. Five members out of five must be present for the safety committee meeting. The DSMB includes experts in or representatives of the fields of onco-hematology , gastroenterology, and statistics / clinical trials methodology.
- Quorum – for cohort review meeting, a quorum will occur when 3 members are present (inclusive of the DSMB Chair). Five members out of five is necessary for the global assessment of safety and tolerability at the end of the study.
- Each DSMB member will be expected to serve for the duration of the trial; in the unlikely event that a member is unable to continue participation, the reason will be documented and a replacement will be selected by MaaT Pharma.

# 5 COMPOSITION OF THE DSMB

Members of the DSMB are:

| NAME | ROLE | EXPERTISE | CONTACT |
|---|---|---|---|
| Jérémie Riou | Member/chair | Biostatistics | jeremie.riou@univ-angers.fr |
| Stéphane Nancey | Member/chair | Gastroenterology | stephane.nancey@chu-lyon.fr |
| Franck Carbonnel | Member/chair | Gastroenterology | fcarbonnel7@gmail.com |
| Frederic Baron | Member/chair | Onco-hematology | f.baron@ulg.ac.be |
| David Liens | Member/chair | Onco-hematology/methodology | dliens.bpls@sfr.fr |

All documentation of **MaaT Pharma** is PROPRIETARY information and may not be forwarded to third parties without prior written consent from **MaaT Pharma.** Reproductions, either in part or in whole, may not be published or copied in any manner, without the explicit written consent of **MaaT Pharma.**

## 6 INDEPENDENCE OF THE DSMB

It is essential that the judgment of members of the DSMB not be influenced by factors other than those necessary to maintain subject safety, and to preserve the integrity of the study. Independence is essential to ensure that DSMB members are objective and capable of an unbiased assessment of the study's safety and efficacy data. The following will ensure the independence of the DSMB:

- Members of the DSMB will not participate as investigators in any study under review and will not be supervised by study investigators.
- Members of the DSMB must not have a direct interest in knowing or influencing trial outcome or have a financial or intellectual interest in the outcome of any studies under review.
- DSMB members must disclose all pharmaceutical companies, biotechnology companies, and CROs in which they hold financial interest. Members must disclose all consultancies (direct or indirect) with pharmaceutical companies, biotechnology companies, and CROs.

  By agreeing to be a board member, the member is stating that there is no conflict of interest with regard to the trial under review by the DSMB.

## 7 RESPONSIBILITIES OF THE DSMB

The members of the DSMB serve in an individual capacity and provide their expertise, including recommendations regarding the continuation, modification, or termination of the study. The DSMB will review cumulative study data to evaluate safety, study conduct, scientific validity and data integrity of the study. The DSMB has the capacity to make recommendations that might impact continuation or termination of the study.

The responsibilities of the DSMB and its members (as safety committee or cohort review committee members) are:

- To review and approve unanimously the Charter
- To evaluate, on an ongoing basis, the accumulating safety assessments to ensure the ongoing safety of study subjects
- To evaluate, at the end of the study, the global tolerability of the product.
- To select the recommended dose for the phase II study

All documentation of **MaaT Pharma** is PROPRIETARY information and may not be forwarded to third parties without prior written consent from **MaaT Pharma.** Reproductions, either in part or in whole, may not be published or copied in any manner, without the explicit written consent of **MaaT Pharma.**

- To consider factors external to the study when relevant information becomes available, such as scientific or therapeutic developments that may have an impact on the safety of the participants or the ethics of the study
- To review all study documents provided by the sponsor
- To review the conduct of the study, including protocol violations
- To review data on participant recruitment, accrual, and retention, as well as assessments of data quality, completeness, and timeliness
- To protect the confidentiality of the study data and the DSMB discussions
- To review specific interim safety data according to the protocol design and make recommendations to continue, modify, or terminate the study depending upon these reviews
- To operate according to the procedures described in this charter and all procedures of the DSMB.
- To follow conflict of interest guidelines as detailed in this charter (see DSMB Membership).
- To maintain documentation and records of all activities as described below (see DSMB Meetings, DSMB Reports)
- To make recommendations to continue, modify or terminate the study
- To be the referee for the imputability of adverse reactions

# 8 DSMB CHAIR RESPONSIBILITIES

The following responsibilities are those of the DSMB Chair:

- Serves as a voting member
- In case of an equality of votes, the Chair, in addition to his original vote, shall have a second or casting vote
- Facilitates the meetings, assists in the development of the agenda, and ensures that the meeting minutes and recommendation(s) are appropriately documented
- Serves as the primary contact person for the DSMB
- Ensures that those involved in the day-to-day management of the study are excluded from DSMB voting procedures
- Discusses DSMB recommendations with appropriate members of the study team.

All documentation of **MaaT Pharma** is PROPRIETARY information and may not be forwarded to third parties without prior written consent from **MaaT Pharma.** Reproductions, either in part or in whole, may not be published or copied in any manner, without the explicit written consent of **MaaT Pharma.**

- Takes and maintains minutes from DSMB sessions, or delegates another member to do so.

# 9 MEETINGS OF THE DSMB

The frequency of DSMB meetings depends on several factors including the rate of enrollment, safety issues or unanticipated adverse events, and availability of data. The sponsor is responsible for convening meetings, selecting a venue when the meeting is not convened by teleconference, and coordinating the distribution of meeting materials to DSMB members and other meeting participants.

Serious Adverse Events (SAE) will be reported to DSMB members by email as soon as possible by the study CRO, Vigipharm, within 48 hours. After confirmation by Vigipharm if the case is a SUSAR (Suspected unexpected Serious Adverse Reaction), Vigipharm will transfer to DSMB members the CIOMS (Council for International Organisations of Medical Sciences).

## 9.1 Data transfer and meeting format

## 9.2 Mandatory meetings

The initial DSMB meeting should occur preferably before the start of the trial or as soon therafter as possible. At this meeting, the DSMB should discuss the protocol and the DSMB charter which includes triggers set for data review and analyses, guidelines for monitoring the study.

The DSMB (cohort review committee) will review the safety data every 3 (cohort1 completed) or 6 (for all other cohorts) patients until 27. The recommended dose for the phase II study will be communicated by the DSMB members to MaaT Pharma.

In addition, the DSMB will review the data in case of major violation of the protocol that may impact the safety or any safety alert signal.

A final committee (safety committee) will occur at the end of the study to evaluate the global safety and tolerability of Maat033.

Other ad'hoc meeting may be organized at DSMB request.

## 9.3 Minutes

A formal report of the meeting minutes containing recommendations for continuation or modification of the study will be prepared by the DSMB Chair person or designee. A draft report will be sent to the DSMB members prior to distribution to the sponsor. DSMB members will have to review and respond to the draft report. The recommendations will then be sent to the sponsor. It is the responsibility of the sponsor to

All documentation of **MaaT Pharma** is PROPRIETARY information and may not be forwarded to third parties without prior written consent from **MaaT Pharma.** Reproductions, either in part or in whole, may not be published or copied in any manner, without the explicit written consent of **MaaT Pharma.**

distribute recommendations to all investigators. The study team is responsible for forwarding DSMB recommendations to their members.

# 10 RECOMMENDATIONS

The DSMB can recommend that the current study continue without modification, continue with specified modifications, discontinue, or halt or modify the study until more information is available.

# 11 STUDY REVIEW CRITERIA/STOPPING RULES AND GUIDELINES

## 11.1 Individual Stopping Criteria

The DSMB will review data related to individual stopping criteria as detailed in the study protocol. The DSMB may recommend modifications to individual stopping rules if additional safety concerns arise during from their continuing reviews of the study data.

## 11.2 Safety monitoring

The DSMB will review the safety data every 3 (cohort1 completed) or 6 (for all other cohorts) patients until the inclusion of 27 patients with effective drug administration.

The DSMB members will review the global safety and tolerability of Maat033 and will define the recommended dose for the phase II study.

In addition, the DSMB will review the data in case of major violation of the protocol that may impact the safety or any safety alert signal.

## 11.3 Study Stopping Criteria

The DSMB may recommend stopping the study for the following reasons:

- The data show an increased risk of serious adverse effects.
- It becomes clear that successful completion of the study is not feasible (e.g. there is an excess of patient dropout, missing data, lack of recruitment etc).

# 11 AMENDMENTS TO THE CHARTER

This DSMB charter can be amended as needed during the course of the study. Information to be included as amendments will be any modifications or supplements to the reports prepared for the DSMB, as well as amendments to other information addressed in this charter.

All documentation of **MaaT Pharma** is PROPRIETARY information and may not be forwarded to third parties without prior written consent from **MaaT Pharma.** Reproductions, either in part or in whole, may not be published or copied in any manner, without the explicit written consent of **MaaT Pharma.**

All amendments will be documented with sequential version numbers and revision dates, and will be recorded in the minutes of the DSMB meetings. All versions of the charter will be archived in accordance with standard operation procedures of the Good Clinical Practices.

## 12 COMPLETION OF DSMB ACTIVITIES

DSMB activities will be completed when all patients have completed their follow-up and the safety committee has taken place.

## 13 CONFIDENTIALITY

All data provided to the DSMB and all deliberations of the DSMB will be privileged and confidential. The DSMB will agree to use this information to accomplish the responsibilities of the DSMB and will not use it for other purposes without written consent from the study sponsor, as specified in this document. Individual DSMB members must not have direct communication regarding the study outside the DSMB (including, but not limited to the investigators, regulatory agencies, or sponsor) except as authorized by the DSMB.

All documentation of **MaaT Pharma** is PROPRIETARY information and may not be forwarded to third parties without prior written consent from **MaaT Pharma.** Reproductions, either in part or in whole, may not be published or copied in any manner, without the explicit written consent of **MaaT Pharma.**

## APPENDIX A: DSMB MEMBER SIGNATURE PAGE

**Member Information**

**Role: DSMB Chair ☒ Member ☐**

**Voting Rights: Yes ☒ No ☐**

Name: Dr David LIENS

Affiliation: Biopharma & Life Sciences Consulting

Phone: +33 622 58 35 19 Fax:

E-mail address: dliens.bpls@sfr.fr

**Re: DSMB Charter Version 2.0 Date:** 28/02/2022

**I have reviewed the attached DSMB Charter (V2.0) and approve it as written. I understand my role as a member of this DSMB, and will adhere to the confidentiality and conflict of interest policies, as stated in this document.**

**Signature:** ______________________ **Date:** 28/02/2022


All documentation of **MaaT Pharma** is PROPRIETARY information and may not be forwarded to third parties without prior written consent from **MaaT Pharma.** Reproductions, either in part or in whole, may not be published or copied in any manner, without the explicit written consent of **MaaT Pharma.**

## APPENDIX B: CONFLICT OF INTEREST STATEMENT

Data and Safety Monitoring Board (DSMB) – CIMON STUDY

The following situations may pose a possible Conflict of Interest.

- Serving as a part-time, full-time, paid, or unpaid employee of any organizations: (a) that are involved in the study under review, (b) whose products or services will be used or tested in the study under review, or (c) whose products or services would be directly and predictably affected in a major way by the outcome of the study;
- Serving as an officer, member, owner, trustee, director, expert advisor, or consultant of such organizations; and
- Having financial interests or assets – of my own or those of my spouse, dependent children, or organizations with which I am connected – in any organizations meeting the above criteria.

**PLEASE CHECK THE APPLICABLE ITEM BELOW.**

☒ I do not have any of the above interests to report.

☐ I have the following (or attached) interests to report.

- I will notify the Director of Clinical Trials Operations of MaaT Pharma promptly if: a change occurs in any of the above during the tenure of my responsibilities, or I discover that an organization with which I have a relationship meets the criteria for a conflict of interest.
- I am aware of my responsibilities for: maintaining the confidentiality of any non-public information that I receive or become aware of through this activity, and for avoiding using such information for my personal benefit, the benefit of my associates, or the benefit of organizations with which I am connected or with which I have a financial involvement.
- Acceptance of this invitation to serve on the DSMB confirms that: I do not have any financial or other interest with any of the collaborating or competing pharmaceutical firms or other organizations involved in the study that constitute a potential conflict of interest.

| Dr David LIENS | [signature] | 28/02/2022 |
|---|---|---|
| Member's Name | Signature | Date |

All documentation of **MaaT Pharma** is PROPRIETARY information and may not be forwarded to third parties without prior written consent from **MaaT Pharma.** Reproductions, either in part or in whole, may not be published or copied in any manner, without the explicit written consent of **MaaT Pharma.**
