# Supplementary material for: Gut microbiota restoration with oral pooled fecal microbiotherapy after intensive chemotherapy: the phase 1b CIMON trial
Source: Blood Adv. 2025 Apr 10;9(15):3739–49. doi: 10.1182/bloodadvances.2024015571 (PMC12305571; doi:10.1182/bloodadvances.2024015571)
Supplement: Supplemental Methods, Tables, Figures, References, and Appendices [file BLOODA_ADV-2024-015571-mmc3.pdf]

CIMON STUDY

Safety phase I evaluation of MaaT033, a lyophilized full-ecosystem intestinal microbiota delayed-release Capsule, In patients with acute MyelOid Leukemia or High-Risk Myelodysplastic Syndrome after iNtensive chemotherapy

**Drug code/name**	MaaT033
**Study Phase**	I
**EudraCT**	2019-004253-89
**ClinicalTrials.gov Identifier**	NCT04150393
**IND number**	NA
**Study Code**	MPOH05
**Coordinating Investigator**	Pr Christian RECHER<br>Service D'Hématologie<br>Institut Universitaire du Cancéropôle de Toulouse - Oncopole<br>1, avenue Irène Joliot-Curie – 31059 Toulouse Cedex9<br>Phone : +33 (0)5 31 15 63 55<br>Recher.Christian@iuct-oncopole.fr
**Study Sponsor:**	MaaT Pharma<br>317 avenue Jean Jaurès – 69007 Lyon – France<br>contact@maat-pharma.com
**Sponsor Project Manager**	Floriane FAURE<br>Phone : +33 (0)6 60 18 92 89<br>ffaure@maat-pharma.com
**Sponsor Pre- and Clinical Scientist**	Benoît LEVAST<br>Phone : +33 (0)6 60 90 79 42<br>blevast@maat-pharma.com
**Sponsor Chief Medical Officer**	John WEINBERG<br>Phone: +44 7 827 575 217<br>jweinberg@maat-pharma.com

Disclosure Statement: This document contains information that is confidential and proprietary to MaaT Pharma. This information is being provided to you solely for the purpose of evaluating and/or conducting a clinical trial for MaaT Pharma. You may disclose the contents of this protocol only to study personnel under your supervision, your Independent Ethics Committee/Institutional Review Board (IEC/IRB), or duly authorized representatives of regulatory agencies. The foregoing shall not apply to disclosure required by any regulations; however, you will give prompt notice to MaaT Pharma of any such disclosure.

-

All documentation of **MaaT Pharma** is PROPRIETARY information and may not be forwarded to third parties without prior written consent from **MaaT Pharma.** Reproductions, either in part or in whole, may not be published or copied in any manner, without the explicit written consent of **MaaT Pharma**

Version history

Version	Date	Authors	Change description
1.0	November 29th, 2019	Dr R. Carter<br>Dr B. Levast	Initial version for application
2.0	December 16th, 2019	Dr R. Carter<br>Dr B. Levast	Typography correction
3.0	January 21st, 2020	F. Faure	Minor edits on process and collections
4.0	February, 5th, 2020	F.Faure	Minor edits
5.0	February, 18th, 2020	F.Faure	Edits following ANSM request – approved by CPP and ANSM
6.0	June, 08, 2020	F. Faure	Clarifications on inclusion and non-inclusion criteria and minor edits – Not approved
7.0	June, 30, 2020	F.Faure	CPP request: minor edits on HR-MDS population – Approved by CPP and not approved by ANSM
8.0	September, 07, 2020	F. Faure	Edits following ANSM request :<br>- Addition of COVID-19 diagnosis tests (nasopharyngeal and stools at V1, V2, V3 and V4)<br>- COVID-19 infections to be reported as a new fact immediately to ANSM.<br>Approved by CPP and ANSM
9.0	January, 06, 2021	F. Faure	Minor edit: removal of version number of applicable IMPD version and IMPD Annex 4 version. Non-substantial modifications.
10.0	May, 04, 2021	F. Faure	Extension of the methods for diagnosing SARS-CoV-2 virus.<br>Typo correction in Appendix 1

All documentation of **MaaT Pharma** is PROPRIETARY information and may not be forwarded to third parties without prior written consent from **MaaT Pharma.** Reproductions, either in part or in whole, may not be published or copied in any manner, without the explicit written consent of **MaaT Pharma**

1 TABLE OF CONTENTS

1 **TABLE OF CONTENTS** **3**

2 **LIST OF ABBREVIATIONS** **8**

3 **SYNOPSIS** **10**

4 **STUDY FLOW CHART** **14**

5 **ADMINISTRATIVE STRUCTURE AND CONTACT INFORMATION** **15**

5.1 CONTRACT RESEARCH ORGANIZATION (CRO) 15

5.2 CENTRAL LABORATORY 15

5.3 BIOMETRY / STATISTICS 15

5.4 IMP MANUFACTURER 15

5.5 PHARMACOVIGILANCE 15

5.6 STUDY COMMITTEE 15

6 **INTRODUCTION AND RATIONALE** **16**

6.1 INTRODUCTION 16

6.1.1 Scientific background: Microbiota and hematologic malignancies 16

6.1.2 Strategies to restore microbiota homeostasis after intensive chemotherapies 17

6.2 IMP BACKGROUND: “FMT” AND MAAT033 BIOTHERAPEUTIC DEVELOPMENT 17

6.3 MAAT PHARMA NON-CLINICAL EXPERIENCE 18

6.4 MAAT PHARMA CLINICAL EXPERIENCE 18

6.5 BENEFIT – RISK ANALYSIS 19

6.5.1 Clinical experience with Full Ecosystem Microbiota Biotherapeutics manufactured by MaaT Pharma 19

6.5.2 Clinical experience with FMT in CDI or IBD 19

6.5.3 Clinical experience with FMT in immunocompromised patients 19

6.5.4 Benefit / risk evaluation 20

7 **STUDY OBJECTIVES** **22**

7.1 PRIMARY OBJECTIVE 22

All documentation of **MaaT Pharma** is PROPRIETARY information and may not be forwarded to third parties without prior written consent from **MaaT Pharma.** Reproductions, either in part or in whole, may not be published or copied in any manner, without the explicit written consent of **MaaT Pharma**

7.2 SECONDARY OBJECTIVES 22

7.3 EXPLORATORY OBJECTIVES 22

8 INVESTIGATIONAL PLAN 23

8.1 TYPE OF TRIAL 23

8.2 STUDY DESIGN 23
8.2.1 Methodology and study phases 23
8.2.2 Decision rules for choice of dose regimen 23

8.3 ENDPOINTS AND EVALUATIONS 25
8.3.1 Primary endpoint: MaaT033 tolerability 25
8.3.2 Secondary endpoint: Dose regimen evaluation 25
8.3.3 Exploratory endpoints 25

9 STUDY POPULATION 26

9.1 INCLUSION CRITERIA 26

9.2 NON-INCLUSION CRITERIA 26

9.3 NUMBER OF SUBJECTS PLANNED - REPLACEMENT POLICY 26

10 THE INVESTIGATIONAL MEDICINAL PRODUCT (IMP): MAAT033 28

10.1 RESPONSIBILITIES 28

10.2 QUALITATIVE AND QUANTITATIVE COMPOSITION 28

10.3 PACKAGING AND LABELING 29

10.4 SUPPLY, TRANSPORTATION, RECEIPT AND STORAGE CONDITIONS 29

10.5 IMP PRESCRIPTION AND DELIVERY 30

10.6 TREATMENT PROCEDURE 30

10.7 RETRIEVAL AND DESTRUCTION OF UNUSED CAPSULES 30

10.8 CONCOMITANT TREATMENT AND THERAPY 31
10.8.1 Not recommended concomitant treatment 31
10.8.2 Permitted concomitant treatment 31

11 STUDY ASSESSMENTS PROCEDURES 32

11.1 VISIT SCHEDULE – CONDUCT OF THE STUDY 35

-

All documentation of **MaaT Pharma** is PROPRIETARY information and may not be forwarded to third parties without prior written consent from **MaaT Pharma.** Reproductions, either in part or in whole, may not be published or copied in any manner, without the explicit written consent of **MaaT Pharma**

11.1.1 Screening Phase 35
11.1.2 Assessments performed during the entire study 35
11.1.3 Inclusion, Visit1 - D1 - TREATMENT START 35
11.1.4 Phone call - D2 (+2days) 36
11.1.5 INTERIM PHASE, Visit 2 - D7 (D5-D9)..36
11.1.6 New cycle START, Visit 3 - D19 (D14-D24) 37
11.1.7 End of study, Visit 4 - D44 post-inclusion - Cycle STOP 37

11.2 ASSESSMENTS REQUESTED FOR THE STUDY 38

11.3 BIOLOGICAL ANALYSES REQUESTED FOR THE STUDY...39
11.3.1 Blood analysis 39
11.3.2 Feces analysis 40
11.3.3 Management of samples..40

11.4 TERMINATION 40
11.4.1 Patient study withdrawal 40
11.4.2 End of study treatment 41
11.4.3 Maintenance use..41

11.5 PREMATURE DISCONTINUATION OF THE STUDY OR PREMATURE CLOSE-OUT OF A SITE 41
11.5.1 Decided by the Sponsor in the following cases: 41
11.5.2 Decided by the Investigator 42

12 SAFETY 43
12.1.1 Definitions (ICH / GCP)..43
12.1.2 Collection, recording and reporting of adverse events 44
12.1.3 Obligation of the Investigator regarding safety reporting..44
12.1.4 Safety Committee (SC) 48

13 DATA MANAGEMENT 50

13.1 DEFINITION OF SOURCE DATA...50

13.2 SOURCE DOCUMENT REQUIREMENTS...50

13.3 USE AND COMPLETION OF CASE REPORT FORMS (CRFS) AND ADDITIONAL REQUEST 50
13.3.1 Data collection...50
13.3.2 Responsibilities 50

-

All documentation of **MaaT Pharma** is PROPRIETARY information and may not be forwarded to third parties without prior written consent from **MaaT Pharma.** Reproductions, either in part or in whole, may not be published or copied in any manner, without the explicit written consent of **MaaT Pharma**

13.4 DATA MANAGEMENT 51

14 **STATISTICAL CONSIDERATIONS 52**

14.1 GENERAL CONSIDERATIONS 52

14.1.1 Endpoints 52

14.1.2 Handling of missing data 52

14.1.3 Type 1 error 53

14.2 ANALYSIS POPULATIONS 53

14.3 STATISTICAL METHODS 53

14.3.1 Planned analyses 53

14.3.2 Descriptive statistics 53

14.3.3 Demographics and medical history 53

14.3.4 Treatment exposure 53

14.3.5 Analysis of the main criterion 53

14.3.6 Analysis of the secondary criteria 54

14.3.7 Analysis of exploratory endpoints 54

14.3.8 Interim analyses 54

14.3.9 Sample size justification 54

15 **ETHICAL AND REGULATORY STANDARDS 55**

15.1 ETHICAL PRINCIPLES 55

15.2 LAWS AND REGULATIONS 55

15.3 INFORMED CONSENT 55

15.4 INSTITUTIONAL REVIEW BOARD/INDEPENDENT ETHICS COMMITTEE (IRB/IEC) 55

16 **STUDY CONDUCT CONSIDERATIONS 57**

16.1 RESPONSIBILITIES OF THE INVESTIGATOR(S) 57

16.2 RESPONSIBILITIES OF THE SPONSOR 57

16.3 SOURCE DOCUMENT REQUIREMENTS 57

17 **ADMINISTRATIVE RULES 59**

17.1 CURRICULUM VITAE 59

17.2 RECORD RETENTION IN STUDY SITES (S) 59

-

All documentation of **MaaT Pharma** is PROPRIETARY information and may not be forwarded to third parties without prior written consent from **MaaT Pharma.** Reproductions, either in part or in whole, may not be published or copied in any manner, without the explicit written consent of **MaaT Pharma**

18 **CONFIDENTIALITY....60**

19 **PROPERTY RIGHTS....61**

20 **DATA PROTECTION....62**

21 **SPONSOR AUDITS AND INSPECTIONS BY REGULATORY AGENCIES....63**

22 **CLINICAL TRIAL RESULTS....64**

23 **PUBLICATIONS AND COMMUNICATIONS....65**

24 **CLINICAL TRIAL PROTOCOL AMENDMENTS....66**

25 **BIBLIOGRAPHIC REFERENCES....67**

26 **APPENDICES....69**

26.1 APPENDIX 1: GUT MICROBIOTA-PERTURBING ANTIBIOTICS....69

26.2 APPENDIX 2: BIRTH CONTROL METHODS....70

-

All documentation of **MaaT Pharma** is PROPRIETARY information and may not be forwarded to third parties without prior written consent from **MaaT Pharma.** Reproductions, either in part or in whole, may not be published or copied in any manner, without the explicit written consent of **MaaT Pharma**

2 LIST OF ABBREVIATIONS

ADR	Adverse Drug Reaction
AE / SAE	Adverse Event / Serious Adverse Event
ANC	Absolute Neutrophil Count
ATG	Antithymocyte Globulin
aGVHD	Acute Graft Versus Host Disease
ALT	Alanine Transaminase
AST	Aspartate Transaminase
ATC	Anatomical Therapeutic Chemical Classification System
BMI	Body Mass Index
CD	Crohn's Disease
CDI	*Clostridium difficile* Infection
CMV	Cytomegalovirus
CPB	Carbapenemase Producing Bacteria
CR	Complete Response
CRO	Contract Research Organization
HR-MDS	High Risk Myelodysplastic syndrome
Hs-CRP	High Sensitivity - C-Reactive Protein
CS	Cortico-Steroids
CTCAE	Common Terminology Criteria for Adverse Events
DBP	Diastolic Blood Pressure
DLT	Dose Limiting Toxicity
DNA	Deoxyribonucleic Acid
DRF	Discrepancy Resolution Form
DSMB	Data Safety Monitoring Board
EBV	Epstein Barr Virus
EC/IRB	Ethics Committee / Institutional Review Board
ECP	Extracorporeal Photopheresis
eCRF	Electronic Case Report Form
EDTA	Ethylene Diamine Tetra-acetic Acid
ESBL	Extended Spectrum β Lactamase
FAS	Full Analysis Set
FMT	Fecal Microbiota Transfer
FPI	First Patient In
GCP	Good Clinical Practice
GGT	Gamma Glutamyl Transpeptidase
GI	Gastrointestinal
HR MDS	High-Risk myelodysplastic syndrome
HSCT	Hematopoietic Stem Cell Transplantation
IBD	Inflammatory Bowel Disease
IBS	Inflammatory Bowel Syndrome
IC	Immunocompromised
ICH	International Council for Harmonization of Technical Requirements for Pharmaceuticals for Human Use
ICU	Intensive Care Unit

-

All documentation of **MaaT Pharma** is PROPRIETARY information and may not be forwarded to third parties without prior written consent from **MaaT Pharma.** Reproductions, either in part or in whole, may not be published or copied in any manner, without the explicit written consent of **MaaT Pharma**

IMP	Investigational Medicinal Product
ITT	Intention-To-Treat
LDH	Lactate Dehydrogenase
LPLV	Last Patient Last Visit
MAA	Market Authorization Application
MED	Minimum Effective Dose
MDRB	Multi-Drug Resistant Bacteria
MRSA	Methicillin-Resistant *Staphylococcus aureus*
MTD	Maximum Tolerated Dose
NIH	National Institute of Health
PEG	Poly-Ethylene Glycol
PP	Per Protocol
PR	Partial Response
RBC	Red Blood Count
RCT	Randomized Controlled Trial
SBP	Systolic Blood Pressure
SOC	Standard of Care
SUSAR	Suspected Unexpected Serious Adverse Reaction
TNFa	Tumor Necrosis Factor alpha
UC	Ulcerative Colitis
VGPR	Very Good Partial Response
VRE / GRE	Vancomycin / Glycopeptid-Resistant Enterococci
WBC	White Blood Count

-

All documentation of **MaaT Pharma** is PROPRIETARY information and may not be forwarded to third parties without prior written consent from **MaaT Pharma.** Reproductions, either in part or in whole, may not be published or copied in any manner, without the explicit written consent of **MaaT Pharma**

3 SYNOPSIS

**Title**	**Safety phase I evaluation of MaaT033, a lyophilized full-ecosystem gut microbiota delayed-release Capsule, In patients with Acute MyelOid Leukemia or High-Risk Myelodysplastic Syndrome after iNtensive chemotherapy**<br><br>**CIMON STUDY**
**SPONSOR**	MaaT Pharma
**EudraCT No.**	2019-004253-89
**INVESTIGATIONAL PRODUCT**	**8P013 / MaaT033, Delayed-Release Capsules of Allogeneic, Pooled, Full-Ecosystem Intestinal Microbiota**
**STUDY RATIONALE**	Richness and diversity of gut microbiota are increasingly found to be associated with cancer outcomes. Moreover, an adequately responsive immune system seems to rely on the existence of a functional gut ecosystem that includes the microbiota and its natural environment.<br>Cancer by itself, but also cancer treatments – in particular chemotherapy – induce gut dysbiosis, impair the constant reparation mechanisms of the gut epithelium, disrupt immune homeostasis, and stunt immune responsiveness.<br>The objective of MaaT033 is (1) to prevent the decay of the gut ecosystem (dysbiosis), (2) to restore and ideally also optimize the gut ecosystem to its fullest functionality including its pivotal role in repairing the gut epithelium and thereby also restoring a healthy gut barrier, and (3) to preserve and possibly improve immune homeostasis for better clinical outcomes in cancer patients.<br>Restoring the full gut ecosystem and its associated microbiota could become an important therapeutic option to improve clinical outcomes and control adverse events of conventional approaches, including immunotherapy in cancer patients.<br>As a first step, MaaT033 capsules containing lyophilized, pooled, full-ecosystem microbiota in its natural environment are to be tested for their safety and tolerability in patients with Acute Myeloid Leukemia (AML) or High-Risk Myelodysplastic syndrome (HR-MSD), who are exposed to intensive rounds of chemotherapy and antibiotics.
**TYPE OF STUDY**	Interventional phase I trial
**STUDY OBJECTIVES**	**Primary**:<br>Evaluation of maximal tolerable dose of MaaT033 in patients with AML or HR-MSD<br><br>**Secondary**:<br>Evaluation of overall safety<br>Dose regimen evaluation: identification of a recommended phase II dose (RP2D) based on gut microbiota engraftment<br><br>**Exploratory**:

All documentation of **MaaT Pharma** is PROPRIETARY information and may not be forwarded to third parties without prior written consent from **MaaT Pharma.** Reproductions, either in part or in whole, may not be published or copied in any manner, without the explicit written consent of **MaaT Pharma**

	- Assessment of a microbiota signature<br>- Impact of our full ecosystem intestinal microbiota biotherapeutic (MaaT033) on the immune system
**STUDY DESIGN**	Open-label, single-arm phase 1 study to evaluate the safety of MaaT033 in a step-up dosing design from 1 capsule per week to 9 capsules per day for 7 or 14 days.
**STUDY POPULATION**<br>**Criteria for Inclusion:**	For eligibility into the trial, patients must meet all the following inclusion criteria:<br>1. Male or Female<br>2. Age ≥ 18 years<br>3. Patients diagnosed with AML defined according to WHO 2016 criteria with ≥20% leukemic blasts in the bone marrow or with high- risk myelodysplastic syndrome, receiving intensive chemotherapy<br>4. Patients healthy enough to likely receive their consolidation or second cycle of chemotherapy after induction chemotherapy<br>5. Patients healthy enough to likely receive HSCT<br>6. Informed written consent<br>7. Patient recovered from neutropenia
**Criteria for non-inclusion:**	Patients meeting any of the following criteria will not be included into the trial:<br>1. Acute promyelocytic leukemia (AML-M3)<br>2. AML secondary to myeloproliferative disorder or chronic myelomonocytic leukemia (CMML)<br>3. Acute myeloid leukemia BCR-ABL1+<br>4. Active CNS leukemia<br>5. Patients with a life expectancy of <70 days according to investigator's opinion, or subject to therapeutic limitations<br>6. Confirmed or suspected intestinal ischemia<br>7. Confirmed or suspected toxic megacolon or gastrointestinal perforation<br>8. Active uncontrolled infection according to the attending physician<br>9. Any gastro-intestinal bleeding in the past 3 months<br>10. Any history of gastro-intestinal surgery in the past 3 months<br>11. Any history of inflammatory bowel disease<br>12. Any counter-indication to swallow capsules<br>13. Enrollment in another trial that may interfere with this study<br>14. Known allergy or intolerance to trehalose, maltodextrin or PEG<br>15. Women of childbearing potential without efficient contraceptive protection<br>16. Pregnant or breastfeeding<br>17. Patients with EBV-negative serology<br>18. Subject who, in the judgment of the Investigator, is likely to be non-compliant or uncooperative during the study, or unable to cooperate because of a language problem, poor mental development;<br>19. Exclusion period of a previous study<br>20. Administrative or legal supervision<br>21. Confirmed positive result to SARS-CoV-2 test at screening
**ENDPOINTS**	**Primary endpoint: MaaT033 tolerability**<br><br>Occurrence of MaaT033 limiting toxicity-related treatment emergent (serious) adverse events, grade≥3, from treatment start (visit 1) to the next cycle (visit 3). AE and SAE relationship will be validated by a final safety committee. Cf safety description § section12. All adverse events will be categorized according to the CTCAE v5.0 criteria.

-

All documentation of **MaaT Pharma** is PROPRIETARY information and may not be forwarded to third parties without prior written consent from **MaaT Pharma.** Reproductions, either in part or in whole, may not be published or copied in any manner, without the explicit written consent of **MaaT Pharma**

	**Secondary endpoints**<br>MaaT033 best dose regimen will be evaluated based on different evaluation:<br><br>- Overall safety will be evaluated from V1 to V4. All AEs will be evaluated by the safety committee.<br>- The product activity:<br><br>Definition of activity1 is here described by the evaluation of the microbiota modification: the proportion and number of different OTUs that colonize the gastro-intestinal tract of the patients. Assessment will be calculated using an alpha-diversity index (*i.e*. Richness, Shannon, Simpson or other ecology indexes).<br><br>Definition of activity2 is here described by the IMP bacterial engraftment: the percentage of bacteria that colonize the gastro-intestinal tract of the patients and come from MaaT033, as compared to baseline (visit1). Assessment will be calculated using Operational Taxonomic Units (OTUs) or a beta-diversity index (*i.e.* UniFrac, Jaccard, Bray-Curtis or other ecology indexes).<br><br>Activities1&2 will be assessed at V2, V3 and V4, as compared to the baseline V1.<br><br>- Patient compliance:<br><br>Based on the level of compliance of the different cohorts evaluated with a daily follow up using a diary, the best dose regimen in terms of activity may be re-considered. A compliance under 60% for a patient would not be considered acceptable to validate the corresponding dose regimen.<br><br>**Exploratory endpoints: Evaluation of MaaT033's impact on patients' gut colonization**<br>Examination at, and evolution from V1 to V4, of the composition of the intestinal microbiota<br>Analysis includes but is not limited to the evaluation of the microbiota modification, based on diversity, and the richness index – using 16S and/or shotgun next generation sequencing (NGS) technology<br>Examination of gut inflammation markers (stool, blood)<br>Based on molecular analyses, description of gut resistome carriage from V1 to V4
**NUMBER OF SUBJECTS**	The enrollment into the study will continue until a maximum of 27 subjects are treated with MaaT033 and evaluable, based on the step-up and safety process:<br><br>**27 patients – 5 cohorts**
**ESTIMATED NUMBER OF SITES**	**3-4 in France**
**STUDY TREATMENT**	Route of administration: oral

-

All documentation of **MaaT Pharma** is PROPRIETARY information and may not be forwarded to third parties without prior written consent from **MaaT Pharma.** Reproductions, either in part or in whole, may not be published or copied in any manner, without the explicit written consent of **MaaT Pharma**

	After recovering from neutropenia (neutropenia is defined as Poly-Nuclear Neutrophils count ≤ $0.5*10^9$ cells/L)<br>Dose regimen:<br>- Cohort 1 (3 patients): 1 capsule (*per os*) at day1 and at day7, e.g., on a Tuesday and the Monday of the following week. Step up of dose regimen will then be as follows:<br>- Cohort 2 (6 patients): 1 capsule/day (corresponding to a maximum of 7 capsules) – 7 days (period of treatment)<br>- Cohort 3 (6 patients): 3 capsules/day (corresponding to a maximum of 21 capsules) – 7 days<br>- Cohort 4 (6patients): 3 capsules/day (corresponding to a maximum of 42 capsules) – 14 days<br>- 9 capsules/day (corresponding to a maximum of 63 capsules) – 7 days
**ASSESSMENT/ SCHEDULE**	Estimated inclusion period: 5 to 7 months<br>Calculated total duration of the study from start to last patient last visit: 7 to 9 months
**DURATION OF STUDY PERIOD (per Subject)**	**Participation period for a patient**:<br>Total potential duration: up to 6-7 weeks

All documentation of **MaaT Pharma** is PROPRIETARY information and may not be forwarded to third parties without prior written consent from **MaaT Pharma.** Reproductions, either in part or in whole, may not be published or copied in any manner, without the explicit written consent of **MaaT Pharma**

4 STUDY FLOW CHART

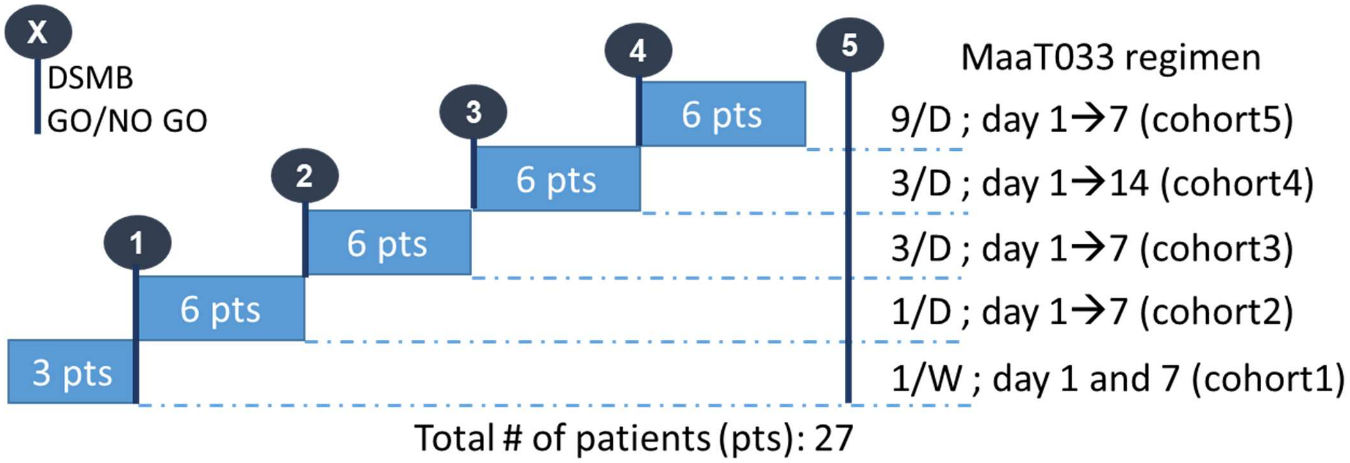

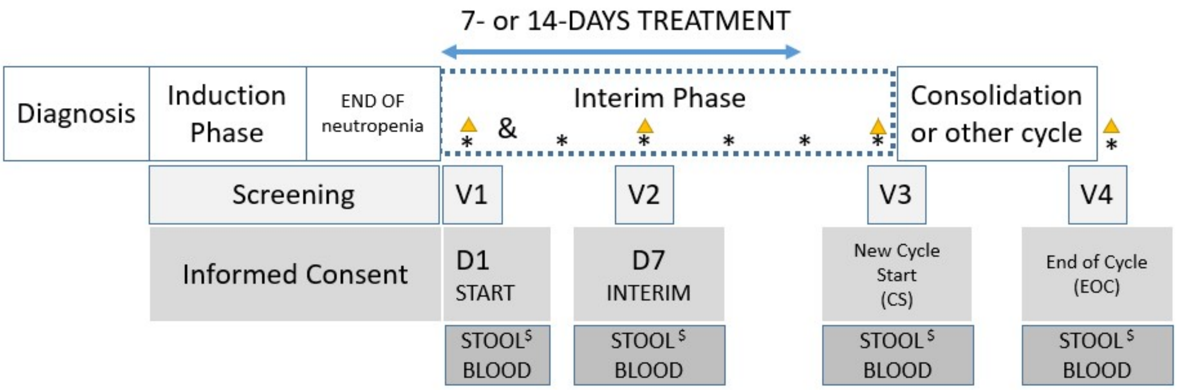

* Faeces collection at home (or at Hospital) at days 1, 4, 7, 10, 14, CS and EOC

$ Stool: fecal swabs collection at V1, V2, V3 and V4

& Phone call

▲ SARS-CoV-2 (nasopharyngeal tests or other validated methods of detection) and fecal tests

V1: Start of the treatment (day1) - End of induction neutropenia

V2: Safety interim visit (day7 +/-2) - Clinical assessment visit

V3: Cycle start (day 19 +/- 5days)

V4: End of the study (day44 +/-10days) = End of cycle

All documentation of **MaaT Pharma** is PROPRIETARY information and may not be forwarded to third parties without prior written consent from **MaaT Pharma.** Reproductions, either in part or in whole, may not be published or copied in any manner, without the explicit written consent of **MaaT Pharma**

5 ADMINISTRATIVE STRUCTURE AND CONTACT INFORMATION

5.1 CONTRACT RESEARCH ORGANIZATION (CRO)

Eurofins-Optimed
1 rue des Essarts
38610 Gières, France

5.2 CENTRAL LABORATORY

Eurofins-Optimed
1 rue des Essarts
38610 Gières, France

Biofortis Mérieux Nutrisciences (for SARS-CoV-2 tests)
3 route de la Chatterie
44800 St Herblain, France

5.3 BIOMETRY / STATISTICS

Eurofins-Optimed
1 rue des Essarts
38610 Gières, France

5.4 IMP MANUFACTURER

Lyofal
Z.A. Gandonne, Rue du Rémoulaire
13300 Salon de Provence

5.5 PHARMACOVIGILANCE

VIGIPHARM
265 rue Maurice Béjart
34 080 Montpellier, France

5.6 STUDY COMMITTEE

Safety & Cohorts Review Committee (Data and Safety Monitoring Board)

All documentation of **MaaT Pharma** is PROPRIETARY information and may not be forwarded to third parties without prior written consent from **MaaT Pharma.** Reproductions, either in part or in whole, may not be published or copied in any manner, without the explicit written consent of **MaaT Pharma**

6 INTRODUCTION AND RATIONALE

6.1 INTRODUCTION

6.1.1 Scientific background: Microbiota and hematologic malignancies

While there is a growing body of evidence highlighting the interactions between the microbiota and allo-HSCT outcomes, little information is available about the role of the microbiota in patients with hematological malignancies treated with intensive chemotherapy outside the allo-HSCT setting (e.g., acute myeloid leukemia -AML- or high-risk myelodysplastic syndrome - HR MDS - patients).

AML patients are relatively rare, and the disease has a high fatality rate. After diagnosis, treatment of AML in otherwise fit patients includes an induction phase with intensive chemotherapy, followed by one or several cycles of intensive consolidation chemotherapy that may or may not lead to the patient receiving a hematologic stem cell transplantation (HSCT). HR-MDS patients are also rare and therapeutic options at diagnostic are mainly limited to 7-day azacitidine chemotherapy regimen or decitabine, also used in AML. Patients present a high risk of progression to AML and some of them may start a therapeutic sequence with intensive chemotherapy. HSCT is also a therapeutic option that may be considered.

The different treatment phases of AML or HR-MDS require extended hospital stays in protected environments due to the high risk of infectious complications in these immune-compromised patients. Indeed, opportunistic infections are relatively frequent in these patients; and can be life-threatening despite the use of prophylactic antibiotic treatments (gut decontamination, probabilistic antibiotherapy in case of fever of unknown origin, *etc*) [1–3]. Currently, no microbiological screening upon patient admission is mandatory but in practice, several analyses can be done according to the physician (bacteriological screening in case of fever; parasitological and viral (HIV, HCV, HBV, HTLV, CMV) screening depending on the context (patient's origin, international travels)). Screening for MDRB (Multi-Drug Resistant Bacteria) is becoming more common in hospitals due to the arrival of patients from various geographic origins and would help guide effective antibiotherapy during chemotherapy-induced aplasia.

Treatments of hematological malignancies are recently known to impact gut microbial composition. Diversity indices are mathematical measures of species diversity in a community and provide information about bacterial microbiota composition such as total numbers (richness), proportions and evenness of species distribution. These indices (Simpson, Simpson reciprocal or inverse Simpson, and Shannon indexes) are simple tools used by bacteriologists to estimate microbial diversity that is known to be affected and reduced in case of dysbiosis and related to several diseases. Interestingly, a recent study examined the microbial intestinal diversity of 80 patients receiving allo-HSCT with a conditioning regimen of chemotherapy and broad-spectrum antibiotics [4]. Fecal samples were collected within 7 days following stem cell engraftment and subjects were classified using inverse Simpson index into high (>4), intermediate (2-4) and low (<2) diversity groups. Overall survival was followed within a 3-years follow-up period and was significantly different between the 3 groups. Mortality outcomes were significantly worse in patients with lower intestinal diversity with a particularly strong effect on mortality after multivariate adjustment for other clinical predictors. Overall survival at 3 years was 36%, 60%, and 67% for low, intermediate, and high diversity groups, respectively, indicating that intestinal microbiota diversity at engraftment is an independent predictor of mortality in allo-HSCT recipients.

Furthermore, composition and function of gut microbial communities were shown to be deeply impacted by chemotherapy without any antibiotherapy in a cohort study on 28 patients with non-Hodgkin lymphomas. Stool samples collected before and after chemotherapy were analyzed by 16S sequencing. Alpha and beta-diversity comparisons of the gut microbiomes revealed taxonomic and functional shifts following chemotherapy. The latter results strongly suggest that not only antibiotherapy but also chemotherapy itself can modify the gut microbiota composition during hematological malignancy treatments, with subsequent consequences on co-morbidities.

All documentation of **MaaT Pharma** is PROPRIETARY information and may not be forwarded to third parties without prior written consent from **MaaT Pharma.** Reproductions, either in part or in whole, may not be published or copied in any manner, without the explicit written consent of **MaaT Pharma**

6.1.2 Strategies to restore microbiota homeostasis after intensive chemotherapies

Development of strategies to manipulate the gut microbiota to suppress or decrease treatment-related complications in OH patients would be a welcome addition to the standard of care armamentarium. Regenerating and even optimizing the full microbiota diversity by means of full-ecosystem restoration therapy may potentially offer a novel approach to attenuate toxicity of chemotherapy on the gut and immune homesostasis - and by extension to contain associated risks for bloodstream infections. Fecal transplantation appears to be highly effective, with multiple studies reporting response rates approaching 90% in *Clostridium difficile* infected patients with *C. difficile* toxic-infection recurrences [5–11]. Thus, pioneering physicians performed fecal transplants from healthy donors, often from close relatives, to patients with recurrent *C. difficile* infections. Recently, controlled studies demonstrated the effectiveness of fecal transplantation compared with conventional antibiotic administration [12,13]. Resolution of diarrhea associated with *C. difficile* infection without relapse after 10 weeks occurred in 93.8% of patients cured by intestinal infusions of donor feces compared to patients with vancomycin (30.8%). Fecal microbiota in patients with *C. difficile* infections had a reduced bacterial diversity evaluated by the Simpson’s reciprocal index, as compared with otherwise healthy persons. Infusion of donor feces resulted in improvement in the microbial diversity that persisted over time, with quantitative changes in relevant groups of Bacteroidetes, Clostridium and Proteobacteria species.

Thus, it would be very encouraging if strategies to manipulate the gut microbiota could be developed to produce favorable conditions that could minimize treatment-related toxicities in patients with hematological malignancies undergoing intensive chemotherapy [14]. Microbiota diversity could be a surrogate endpoint for monitoring dysbiosis correction after fecal microbiota transplantation.

6.2 IMP BACKGROUND: “FMT” AND MAAT033 BIOTHERAPEUTIC DEVELOPMENT

Details on IMP description and dosing are available in the Investigator Brochure and in the IMP Dossier.

Since the demonstration of positive clinical outcomes with fecal microbiota transfer (FMT) in CDI, the FMT approach is being used today in a wide range of indication and based on a wide range of formulations:

1. Liquid suspension: This formulation consists of a filtered liquid suspension of intestinal microbiota ecosystem material in a diluent, which historically uses simple saline solution (for fresh preparation and immediate administration) or a saline solution with glycerol as a cryopreservative (frozen preparation for subsequent administration).
2. These formulations could either be administered by means of an enema (reaching the distal colon), via colonoscopy (reaching up to the proximal, ascending colon) or if encapsulated and taken orally, eventually reaching the colon via the upper gastrointestinal route.
3. Freeze-dried product: After producing the liquid suspension, it goes through a freeze-drier to obtain a water-free powder. This powder is then encapsulated and administered orally.

Numerous academic, hospital/university studies are ongoing (35 recruiting or completed registered trials on clinicaltrial.gov) or completed [15–18], and summarized in meta-analyses [19]: A total of six studies, five case series and one randomized-controlled trial, were included in this review. Overall, 341 patients completed treatment with encapsulated FMT. Only three major adverse events were reported and no deaths occurred directly related to FMT. In all, 285 patients responded to the first treatment, with no recurrence during the specified follow-up period set to meet the primary endpoint.

Other product candidates are also in the development pipelines of other industrial pharmaceutic companies, providing some element in term of safety and technology.

- Openbiome/Finch Therapeutics: Developed double encapsulated (DE) and CP-101 FMT capsules as oral delivery products. These capsules are being used in clinical trials (NCT03621657-GRAFT-CDI; NCT03110133-CDI; NCT03829878-SPROUT-autism spectrum disorder)

-

All documentation of **MaaT Pharma** is PROPRIETARY information and may not be forwarded to third parties without prior written consent from **MaaT Pharma.** Reproductions, either in part or in whole, may not be published or copied in any manner, without the explicit written consent of **MaaT Pharma**

- 4DPharma: Developed MRx1234 [Blautia Hydrogenotrophica] in IBS indication (NCT03721107); MRx-4DP0004, also a live biotherapeutic in asthma indication. One other candidate is being tested in combination for oncology indications: MRx0518 product consist of a lyophilised formulation of a proprietary strain of bacterium (Enterococcus species) aiming to improve clinical outcome of solid cancer patients waiting surgical removal of the tumor. MRx0518 is also tested in combination with Pembrolizumab (Keytruda, anti PD1) to improve anti-tumor activity in solid cancer patients.
- Seres: Developed RBX7455, a non-frozen, room-temperature-stable oral microbiota-based formulation. The product is being tested in Cdiff prevention (NCT02981316), in operable breast cancer to evaluate intratumoral immunomodulatory effects (NCT04139993) and in pediatric IBS (NCT03378167) in combination with an enema formulation. Also developed RBX7455 is a preparation of live intestinal microorganisms purified from stool donations obtained from healthy, screened donors, mixed with preservative, lyophilized, and put into capsules. The capsules are taken orally. After ingestion, these freeze-dried microorganisms can reconstitute and proliferate in the gut.

6.3 MAAT PHARMA NON-CLINICAL EXPERIENCE

Regarding non-clinical studies and results, please refer to the Investigator Brochure document.

In allogeneic intestinal full-ecosystem microbiota restoration therapies, doses of feces are usually around 50g per transplantation with delivery methods such as naso-gastric, naso-enteric, trans-colonoscopic, enema route or a combination of these. The European FMT Working Group recommends at least 30g of feces for a final volume of 150mL [6].

Our preclinical studies on pigs did not reveal any dose effect. Nevertheless, dose equivalence were done in bioreactor to compare 10g enema, 30g enema and lyophilized product. Our results support a good equivalence between these 3 dosing regimen.

Also, MaaT033 galenic form present a pH dependent release property, thus targeting the ileo-caecal region of the gastro-intestinal system. This property enable the release of the active substance in a lower cell density area as compare to the colon (enema delivery).

We therefore propose in our protocol a targeted dose regimen of 3 capsules per day during 7 or 14 days.

6.4 MAAT PHARMA CLINICAL EXPERIENCE

Historical treatment developed by MaaT Pharma are MaaT011 (autologous, enema, ODYSSEE trial in AML), MaaT012 (single heterologous donor, enema, HAPY trial in intensive care admitted patients) and MaaT013 (pooled heterologous donors, enema, HERACLES trial in GvHD).

The feasibility and process of enema administration are not directly comparable to those of the capsule, even though the starting solution is the same pooled, allogeneic biotherapeutic.

Table of AEs reported in MaaT Pharma clinical trials, Autorisation temporaire d'utilisation (ATU) and compassionate use (CU) program.

Study/Activity	# patients treated	Product	# of SAE	SUSAR description	# of related SAE
ODYSSEE (AML)	25	MaaT011	30 (1 SUSAR)	- Sepsis (E. coli)	1 (Sepsis, possibly related)
HAPY (ICU)	6	MaaT012	5 (2 SUSARs)	- Colonic perforation<br>- Intestinal ischemia	0

-

All documentation of **MaaT Pharma** is PROPRIETARY information and may not be forwarded to third parties without prior written consent from **MaaT Pharma.** Reproductions, either in part or in whole, may not be published or copied in any manner, without the explicit written consent of **MaaT Pharma**

HERACLES (GvHD)	20<br>(Aug 2019)	MaaT013	28 (3 SUSARs)	- Sepsis<br>- Sepsis<br>- Sepsis	0
CU & ATU<br>(GvHD & decolonization)	30<br>(Sept 2019)	MaaT012 /MaaT013	1 (Sepsis)	NA	0
TOTAL	81		66		1

Overall, the safety of MaaT products is good. MaaT013 is the enema version of the exact same pooled heterologous active biotherapeutic as MaaT033.

6.5 BENEFIT – RISK ANALYSIS

6.5.1 Clinical experience with Full Ecosystem Microbiota Biotherapeutics manufactured by MaaT Pharma

Regarding clinical studies and results, please refer to the Investigator Brochure document.

We chose capsules to enable the overall “easier” and more acceptable oral delivery of our biotherapeutics. Other delivery routes have potential important side effects. The enema and colonoscopy carry a risk of colonic perforation; the upper route intubation of vomiting, bloating, upper gastrointestinal hemorrhage, and abdominal pain.

Regarding the period of the treatment, we decided to mimic the Odyssee trial with treatment of patients post chemotherapy. Indeed, the period of treatment is defined after aplasia/neutropenia recovery like in Odyssee protocol. Safety post treatment of the Odyssee trial was good, thus we also do expect a good tolerability and safety of the MaaT033 product.

6.5.2 Clinical experience with FMT in CDI or IBD

Rossen *et al*. performed a systematic review to analyze the clinical efficacy and safety of FMT used as clinical therapy [20]. They were able to identify 45 studies; 34 on *Clostridium difficile*-infection (CDI), 7 on inflammatory bowel disease, 1 on metabolic syndrome, 1 on constipation, 1 on pouchitis and 1 on irritable bowel syndrome (IBS). In CDI, 90% resolution of diarrhea in 33 case series (n = 867) was reported, and 94% resolution of diarrhea after repeated FMT in a randomized controlled trial (RCT) (n = 16). In ulcerative colitis (UC) remission rates of 0% to 68% were found (n = 106). In Crohn’s disease (CD) (n = 6), no benefit was observed. In IBS, 70% improvement of symptoms was found (n = 13). Reversal of symptoms was observed in constipation (100%) (n = 3). In pouchitis, none of the patients (n = 8) achieved remission. One randomized controlled trial (RCT) showed significant improvement of insulin sensitivity in metabolic syndrome (n = 10). Serious adverse events (SAE) were rare. SAE were reported in 34 out of 45 studies. Two RCTs reported mild adverse events attributed to FMT, including diarrhea, cramping, belching, nausea, abdominal pain, bloating, transient fever, and dizziness.

Overall, based on the above studies, the safety profile of FMT proved to be excellent.

6.5.3 Clinical experience with FMT in immunocompromised patients

Literature on FMT in immunocompromised patients is still scant, however, preliminary data are encouraging. One case-series study reported on FMT use in 80 immunocompromised patients [14]. Although serious

All documentation of **MaaT Pharma** is PROPRIETARY information and may not be forwarded to third parties without prior written consent from **MaaT Pharma.** Reproductions, either in part or in whole, may not be published or copied in any manner, without the explicit written consent of **MaaT Pharma**

adverse events (2 deaths and 10 hospitalizations) occurred in 12 patients (15%), none seemed to be directly related to FMT.

A retrospective study done on immunocompromised (IC) and non-immunocompromised (non-IC) patients who received FMT for recurrent CDI concludes that response to FMT is equivalent in IC and non-IC populations. When comparing the percentage of SAEs between IC and non-IC patients, no significant difference was found [21]. Until today, 15 published case reports or case series have described the use of FMT in IC patients for a total of 132 treated patients.

In the severely immunocompromised patients following allo-HSCT, concerns about infectious organisms transmitted through this procedure need to be addressed. Heterologous FMT has been evaluated in 14 IC patients after allo-HSCT [22,23] without any severe, FMT-related adverse effect reported.

Webb et al. reported the use of heterologous FMT in 7 patients with recurrent CDI after allo-HSCT. FMT was administered via the naso-jejunal route in 6 of 7 patients. No serious adverse events were noted in these immunocompromised patients. Diarrhea improved in all patients, 6 of 7 (86%) patients had no recurrence of symptoms. Therefore, FMT appears to be safe for recurrent CDI in immunocompromised allo-HSCT patients.

In the context of GVHD, the authors of the 2 studies did not observe any immediate procedure-related infections despite the temporary withdrawal of prophylactic antibiotics to protect the transplanted microbiome. Temporal withdrawal of broad-spectrum antibiotics might be a prerequisite of successful FMT since some antibiotics are associated with perturbation of gut microbial composition and increased GvHD-related mortality [24]. Several infectious events occurred during the follow-up of the patients [25] but the authors could not firmly exclude that infections with intestinal pathogens were induced by FMT. Due to the long-term immunosuppressive therapy, such infections are common in the natural course of patients with HSCT and aGvHD and no excess of infections after FMTs was observed.

6.5.4 **Benefit / risk evaluation**

Overall, the safety profile of FMT is very good with few side effects and preliminary studies showed promising results of efficacy in various indications: CDI, IBD, GvHD, and decolonization of MDRB. In view of the medical needs of patients with hematological malignancies and the limited therapeutic options, FMT might be a beneficial treatment modality in this challenging clinical situation and thus substantially improve the prognosis of these patients.

Of note, recent report of 2 deaths in CDI patients due to MDRB (extended-spectrum beta lactamase) translocation from the FMT product underline the importance of safety monitoring with such treatment and the importance of a complete quality process for drug production. MaaT Pharma do provide such microbiological screening on donations, including MDRB.

Besides the expected low toxicity, there is a high pathophysiological rationale by restoring their intestinal ecosystem in patients following intensive chemotherapy: 1) Several recent studies showed that low diversity at the time of HSCT predicts poor outcome of GI-GVHD 2) Experimental studies showed, that reconstitution of a commensal microbiota restores intestinal short chain fatty acid levels which are essential for epithelial regeneration but also support anti-tumoral immunity, which are most important to restore immune-homeostasis. None of the currently available strategies has selective beneficial effects on immunity.

Comparison between enema FMT and capsule FMT also provide some evidence of increased benefit/ratio while considering MaaT033. Oral route is of importance as the patient will benefit a convenient treatment, easy to use. On the opposite, lower route such as enema, presenting risk of colonic perforation, is much less convenient in term of treatment procedure. While lyophilization process might reduce activity of the product, we provided in the IMPD the demonstration of a good potency. MaaT033 capsule present a specific property to release the active substance in the ileo-caecal region. This delivery area is of interest as such new galenic allow a specific release in the ileo-caecal region of the active substance that will lead to an optimal activity and mechanism of action throughout the entire ileum and colon segments.

-

All documentation of **MaaT Pharma** is PROPRIETARY information and may not be forwarded to third parties without prior written consent from **MaaT Pharma.** Reproductions, either in part or in whole, may not be published or copied in any manner, without the explicit written consent of **MaaT Pharma**

Overall, the benefit/risk ratio of MaaT033 is positive, with a minimal intrusive treatment with capsules containing a full ecosystem of donors gut microbiota that will restore gut eubiosis in severely affected patients after intensive chemotherapy.

All documentation of **MaaT Pharma** is PROPRIETARY information and may not be forwarded to third parties without prior written consent from **MaaT Pharma.** Reproductions, either in part or in whole, may not be published or copied in any manner, without the explicit written consent of **MaaT Pharma**

7 STUDY OBJECTIVES

7.1 PRIMARY OBJECTIVE

Evaluation of maximal tolerable dose of MaaT033 in patients with AML or HR-MSD

7.2 SECONDARY OBJECTIVES

Evaluation of overall safety

Dose regimen evaluation: identification of a recommended phase II dose (RP2D) based on gut microbiota engraftment

7.3 EXPLORATORY OBJECTIVES

- Assessment of a microbiota signature
- Impact of our full ecosystem intestinal microbiota biotherapeutic (MaaT033) on the immune system

Corresponding endpoints are detailed in § section 8.3.

-

All documentation of **MaaT Pharma** is PROPRIETARY information and may not be forwarded to third parties without prior written consent from **MaaT Pharma.** Reproductions, either in part or in whole, may not be published or copied in any manner, without the explicit written consent of **MaaT Pharma**

8 INVESTIGATIONAL PLAN

8.1 TYPE OF TRIAL

Interventional phase I trial

8.2 STUDY DESIGN

8.2.1 Methodology and study phases

Open-label, single-arm phase 1 study to evaluate the safety of MaaT033 in a step-up dosing design from 2 capsules 7 days apart to 9 capsules per day for 7 days.
Choice of cohort is based on the definition of maximum and minimum dose regimen acceptable for good compliance. 1 capsule per week is a reasonable minimum dosing while 9 capsules per day is a maximum we could define for such a population, already having quite important medications.
Our target dose is also in between these 2 cohorts, with 3 capsules per day. We decided to assess the impact of treatment duration with the comparison between a 7 days courses versus 14 days.

The study has the following phases:

- **Patient pre-screening phase** - Patients diagnosed with hematologic malignancy with will be asked if they are willing to participate in this study by the investigating physician and will sign the informed consent form before any study examination. Only patients with neutropenia recovery will be finally included in the study.
  Neutropenia recovery is defined as a level of Absolute Neutrophil Count (ANC) ≥0.5*10^9 cells/L.

- **Treatment phase** - The treatment will be started within 2 days after patient's inclusion. Clinical evaluation will be performed at baseline, at the interim visit (day7), before new cycle of chemotherapy start if any (day19 if not) and after new cycle chemotherapy if any (day44 if not). A step-up process will be applied between 5 different cohorts of patients and will depend on patient's tolerance of the treatment.

- **Follow-up phase**:
  Patient's overall status will be evaluated at D44 post inclusion (V4).
  AE/SAE collection will be performed from time of informed consent to V4.

8.2.2 Decision rules for choice of dose regimen

This study has for objective to assess MTD and MAD in term of MaaT033 activity (bacteria engraftment). Activity assessment requires a minimum of 6 patients to allow for a sufficient robust analysis of engraftment.

Activity and tolerability assessment may depend both on duration of treatment and/or daily dose. Therefore, the study will assess both 7 days and 14 days dose regimen, for the 3 capsules/day dosing.

Therefore, the study will ensure that a minimum of 6 patients is included for all the dose regimen that do not reach the MTD.

Definitions:
Minimal Active Dose (MAD): Minimum dose regimen that will lead to a minimal activity (1 or 2, cf chapter 8.3.2) of the product.

All documentation of **MaaT Pharma** is PROPRIETARY information and may not be forwarded to third parties without prior written consent from **MaaT Pharma.** Reproductions, either in part or in whole, may not be published or copied in any manner, without the explicit written consent of **MaaT Pharma**

Dose Limiting Toxicity (DLT): Toxicity event will be defined as any related adverse event grade >3 based on CTCAE v5.0 criteria for:

- Blood and lymphatic system disorders (DLT when grade4 or 5)
- Immune system disorders (DLT when grade4 or 5)

And any related adverse event grade ≥3 for all other system organ.

Maximal Tolerated Dose (MTD): Maximal dose for which only 0 or 1 patient do experience limiting toxicity out of a cohort of 6 patients.

The study has an adapted "6 dose titration" escalation design to allow for assessment of tolerability (research of the Maximal Tolerated Dose) and of activity (research of Minimal Active Dose):

- **1st level of dose for 1st cohort:** - 3 Patients will be treated with one capsule/day on day 1 and D7. The Safety Committee (SC) will evaluate occurrence of Limiting Toxicity after the 3 patients will have completed V3. If no patient experiments a DLT, the following cohort will use dose level 2 *i.e.* 1 capsule/Day for 7 days. If one or more patient experiment a DLT, the SC might consider study stop recommendation or cohort continuation. The purpose of this cohort is to assess feasibility of the whole procedure and check any unexpected tolerability issue that would prohibit continuation of the experimentation. As the dose schedule is not considered as sufficient enough to allow for any effective activity, it will not be considered for the choice of final dose.

- **2nd Level of dose (cohort2):** 6 patients will be treated with 1 capsule/day for 7 days. The Safety Committee (SC) will evaluate occurrence of Limiting Toxicity after the 6 patients will have completed V3. If no or 1 patient experiments a DLT, the following cohort will use dose level 3 *i.e.* 3 capsules/day for 7 days. If two or more patients out of 6 experiment a DLT, the study will be stopped.

- **3rd level of dose (cohort3):** 6 patients will be treated with 3 capsules/day for 7 days. The Safety Committee (SC) will evaluate occurrence of Limiting Toxicity after the 6 patients will have completed V3. If no or 1 patient experiments a DLT, the following cohort will use dose level 4 *i.e.* 3 capsules/D for 14 days and dose level 5 *i.e.* 9 capsules/day for 7 days. If two or more patients out of 6 experiment a DLT, the study will be stopped.

- **4th level of dose:** 6 patients will be treated with 3 capsules/day for 14 days. The Safety Committee (SC) will evaluate occurrence of Limiting Toxicity after the 6 patients will have completed V3. If no or 1 patient experiments a DLT, the following cohort will use dose level 5 *i.e.* 9 capsules/day for 7 days. If two or more patients out of 6 experiment a DLT, the study will be stopped.
  Of note, if 2 or more DLT are reported during the 7 first days of treatment, then the SC will re-assess cohort3 evaluation.

- **5th level of dose:** 6 patients will be treated with 9 capsules/day for 7 days. The Safety Committee (SC) will evaluate occurrence of Limiting Toxicity after the 6 patients will have completed V3. If two patients or more experiment a DLT, the study will be stopped and MTD will be considered as dose level 4. If one patient or less experiments a DLT, the MTD will be considered dose level 5.

In any of the cohort 2 to 5, if any safety signal suggesting potential DLT is reported in 2 or more patient before the end of the inclusion of all patient of the cohort, inclusion of subsequent patient will be suspended and ad hoc meeting of the DSMB will be hold with all available information to assess those potential DLT and decide on the continuation or stopping the study.

A 24-hour gap should be respected between inclusions of the first 3 patients of each cohort to consider potential early or delayed toxicities.

-

All documentation of **MaaT Pharma** is PROPRIETARY information and may not be forwarded to third parties without prior written consent from **MaaT Pharma.** Reproductions, either in part or in whole, may not be published or copied in any manner, without the explicit written consent of **MaaT Pharma**

8.3 ENDPOINTS AND EVALUATIONS

The studied procedure will be considered as safe if:

No severe SAE related to treatment procedure has been documented.

All patients' case profiles will be reviewed by a committee composed by 3 physicians. The main task of this committee will be to evaluate collected data and to give an opinion on treatment safety. The committee will analyze all treatment emergent AE(s) and propose categorization as treatment-related or not. In case of disagreement, majority (2 out of 3) committee's opinion will be considered as final decision.

8.3.1 **Primary endpoint: MaaT033 tolerability**

Occurrence of MaaT033 limiting toxicity-related treatment emergent (serious) adverse events, grade≥3, from treatment start (visit 1) to the next cycle (visit 3). AE and SAE relationship will be validated by a final safety committee. Cf safety description § section12. All adverse events will be categorized according to the CTCAE v5.0 criteria.

Tolerability assessment will be done with the evaluation of LTD. Based on the MTD and MAD evaluated in the different cohorts, the best dose regimen in terms of activity might be re-evaluated. Therefore, the minimal dose that is safe and active will support the best dose regimen that may need to be adjusted correspondingly.

8.3.2 **Secondary endpoint: Dose regimen evaluation**

MaaT033 best dose regimen will be evaluated based on different evaluations:

- Overall safety will be evaluated from V1 to V4. All AEs will be evaluated by the safety committee.

- The product activity:

Definition of activity1 is here described by the evaluation of the microbiota modification: the proportion and number of different OTUs that colonize the gastro-intestinal tract of the patients. Assessment will be calculated using an alpha-diversity index (*i.e.* Richness, Shannon, Simpson or other ecology indexes).

Definition of activity2 is here described by the IMP bacterial engraftment: the percentage of bacteria that colonize the gastro-intestinal tract of the patients and come from MaaT033, as compared to baseline (visit1). Assessment will be calculated using Operational Taxonomic Units (OTUs) or a beta-diversity index (*i.e.* UniFrac, Jaccard, Bray-Curtis or other ecology indexes).

Activities1&2 will be assessed at V1, V2, V3 and V4.

- Patient compliance: Based on the level of compliance of the different cohorts evaluated with a daily follow up using a diary, the best dose regimen in terms of activity may be re-considered. For a patient, a compliance of less than 60% of capsules that were supposed to be ingested would not be considered acceptable to validate the corresponding dose regimen.

8.3.3 **Exploratory endpoints**

- Examination at, and evolution from V1 to V4, of the composition of the intestinal microbiota. Analysis includes but is not limited to the evaluation of the microbiota modification, based on diversity, and the richness index – using 16S and/or shotgun next generation sequencing (NGS) technology
- Examination of gut inflammation markers (stool, blood), immuno-monitoring
- Based on molecular analyses, description of gut resistome carriage from V1 to V4.

-

All documentation of **MaaT Pharma** is PROPRIETARY information and may not be forwarded to third parties without prior written consent from **MaaT Pharma.** Reproductions, either in part or in whole, may not be published or copied in any manner, without the explicit written consent of **MaaT Pharma**

9 STUDY POPULATION

9.1 INCLUSION CRITERIA

Each patient must sign and date the consent form prior to initiating any study-related procedures.

To be eligible for study participation, a subject must meet all the following criteria:

1 Male or Female
2 Age ≥ 18 years
3 Patients diagnosed with AML defined according to WHO 2016 criteria with ≥20% leukemic blasts in the bone marrow or high-risk myelodysplastic syndrome, receiving intensive chemotherapy
4 Patients healthy enough to likely receive their consolidation or second cycle of chemotherapy after induction chemotherapy
5 Patients healthy enough to likely receive HSCT
6 Informed written consent
7 Patient recovered from neutropenia

9.2 NON-INCLUSION CRITERIA

Subjects are not eligible for study participation if they meet any of the following non-inclusion criteria:

1 Acute promyelocytic leukemia (AML-M3)
2 AML secondary to myeloproliferative disorder or chronic myelomonocytic leukemia (CMML)
3 Acute myeloid leukemia BCR-ABL1+
4 Active CNS leukemia
5 Patients with a life expectancy of <70 days according to investigator's opinion, or subject to therapeutic limitations
6 Confirmed or suspected intestinal ischemia
7 Confirmed or suspected toxic megacolon or gastrointestinal perforation
8 Active uncontrolled infection according to the attending physician
9 Any gastro-intestinal bleeding in the past 3 months
10 Any history of gastro-intestinal surgery in the past 3 months
11 Any history of inflammatory bowel disease
12 Any counter-indication to swallow capsules
13 Enrollment in another trial that may interfere with this study
14 Known allergy or intolerance to trehalose, maltodextrin or PEG
15 Women of childbearing potential without efficient contraceptive protection (appendix 2)
16 Pregnant or breastfeeding
17 Patients with EBV-negative serology
18 Subject who, in the judgment of the Investigator, is likely to be non-compliant or uncooperative during the study, or unable to cooperate because of a language problem, poor mental development;
19 Exclusion period of a previous study
20 Administrative or legal supervision
21 Confirmed positive result to SARS-CoV-2 test at screening

9.3 NUMBER OF SUBJECTS PLANNED - REPLACEMENT POLICY

The enrollment into the study will continue until a maximum of 27 subjects are treated with MaaT033 and evaluable, based on the step-up and safety process.

-

All documentation of **MaaT Pharma** is PROPRIETARY information and may not be forwarded to third parties without prior written consent from **MaaT Pharma.** Reproductions, either in part or in whole, may not be published or copied in any manner, without the explicit written consent of **MaaT Pharma**

If the compliance is under 60% for a patient, the patient will not be included in the evaluation of the dose-corresponding regimen but will be replaced by another patient.

If a patient becomes positive to SARS-CoV-2 (either nasopharyngeal, oropharyngeal swabs or salivary or other tests or feces tests) during the treatment period, it will be at the investigator's judgment to decide if the patient continues to take the capsules and stay in the study.

In case of COVID-19 pandemic in certain area of the country, an ongoing patient with unmonitored data can be considered to be replaced by another patient in another site in order to validate the corresponding dose regimen by the DSMB.

-

All documentation of **MaaT Pharma** is PROPRIETARY information and may not be forwarded to third parties without prior written consent from **MaaT Pharma.** Reproductions, either in part or in whole, may not be published or copied in any manner, without the explicit written consent of **MaaT Pharma**

10 THE INVESTIGATIONAL MEDICINAL PRODUCT (IMP): MAAT033

A complete description of the IMP can be found in the IMPD.

10.1 RESPONSIBILITIES

The Sponsor MaaT Pharma has delegated the manufacturing responsibilities to Lyofal.

The Sponsor is responsible to provide Investigational Product supplies and to dispatch them to participating sites.

The Investigator, the Hospital Pharmacist, or other qualified person allowed to receive, store and dispense the Investigational Product will be responsible for ensuring that the Investigational Product used in the clinical trial is securely maintained as specified by the Sponsor and in accordance with the applicable regulatory requirements.

On receipt of the supplies of the IP, the investigator or other authorized personnel will check for accurate delivery and acknowledge the quantity of IP received. **See section** 10.4 .

All investigational products shall be dispensed in accordance with the Investigator's prescription only to subjects enrolled in the study and it is the Investigator's responsibility to ensure that an accurate record of accountability for the Investigational Product received, dispensed and returned is maintained.

An accurate accounting of investigational products will be available for verification by the Sponsor or its representative periodically during monitoring visits for both unused and used containers.

Any quality issue noticed with the receipt or use of an Investigational Product (deficient IP in condition, appearance, pertaining documentation, labeling, expiry date, etc.) should be promptly notified to the Sponsor, who will initiate a complaint procedure.

In the event of a batch recall, the sponsor or its representative will inform the investigator or pharmacist in writing. Upon receipt, and as instructed, the investigator or pharmacist should immediately contact any study subjects in possession of the corresponding IP. The Sponsor is responsible for the batch recall procedure.

Unused IMP must not be discarded or used for any purpose other than the present trial. IMP that has been dispensed to a subject must not be re-dispensed to a different subject. Under no circumstances will the Investigator supply Investigational Product to a third party, allow the Investigational Product to be used other than as directed by this Clinical Trial Protocol, or dispose of Investigational Product in any other manner.

10.2 QUALITATIVE AND QUANTITATIVE COMPOSITION

Test medication: Delayed release capsule of Allogeneic Fecal Microbiota, Pooled.
Active substance: Allogeneic Fecal Microbiota, Pooled. Storage Room temperature.
A capsule of MaaT033 contains:

1. Active substance: Fecal material for therapeutic use collected from selected healthy donors under medical responsibility, according to ANSM guidelines and European recommendations, and consisting of filtered undigested material, microbial populations and their metabolites. Description of donor selection and stool collection process is described in an *ad hoc* technical document (latest applicable version of IMPD and IMPD Annex 4), dedicated for collection center use only. With this procedure, microbiota diversity of the final product will be evaluated for the manufacturing characterization and before batch release.

-

All documentation of **MaaT Pharma** is PROPRIETARY information and may not be forwarded to third parties without prior written consent from **MaaT Pharma.** Reproductions, either in part or in whole, may not be published or copied in any manner, without the explicit written consent of **MaaT Pharma**

2. Cryopreservation diluent allowing the preservation of bacteria during freeze-dried process.

Each capsule of MaaT033 contains the bioequivalence of 0.42g of feces. Number of bacteria per capsule is guaranteed at a minimum of 10^9 cells (absolute number).

10.3 PACKAGING AND LABELING

Capsules will be presented into High-Density PolyEthylene (HDPE) bottles with tamper evident cap (secondary packaging), specifically conceived to properly maintain capsules in good environmental conditions.

Label statements are specific to the clinical trial, complying with legal requirements for medicinal product. One label will be present on the IMP container, where the qualified person will be able to add the patient identification number.

Each patient's kit will include one or several bottles corresponding to one patient treatment; each kit will be composed of capsules containers from the same manufacturing batch.

Kits for patients:

Cohort1: 1 bottle (30ml) of 7 capsules. Patient will return the bottle with 5 capsules in it.

Cohort2: 1 bottle (30ml) of 7 capsules. Patient will return the bottle (empty).

Cohort3: 1 bottle (30ml) of 21 capsules. Patient will return the bottle (empty).

Cohort4: 2 bottles (30ml) of 21 capsules. Patient will return the bottles (empty).

Cohort5: 3 bottles (30ml) of 21 capsules. Patient will return the bottles (empty).

Here is an example of labeling for a 7 capsules bottle packaging (15ml):

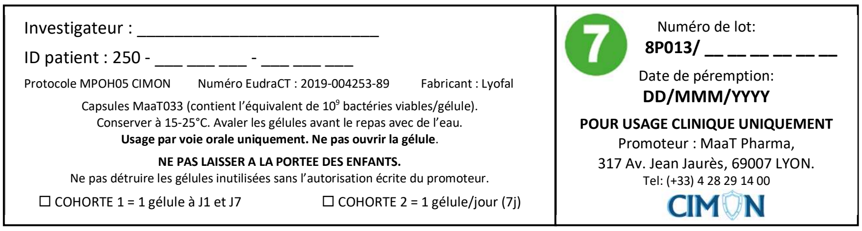

10.4 SUPPLY, TRANSPORTATION, RECEIPT AND STORAGE CONDITIONS

MaaT Pharma will send a stock of MaaT033 capsules to the Investigator's site by a specialized carrier in a qualified box keeping the IMP at controlled temperature. The temperature evolution during shipment will be continuously monitored by a recording sensor inside the pack, provided by the carrier.

If the recording sensor provides evidence that the product underwent excessive absolute temperature during shipment, the pharmacist / investigator / qualified person must contact MaaT Pharma for further instructions.

Details regarding the IMP reception should be documented by the recipient:

3. The integrity of the container (absence of visual break, tamper evidence)
4. The temperature indicator attached to the container must show no alert sign that the product underwent excessive absolute temperature

-

All documentation of **MaaT Pharma** is PROPRIETARY information and may not be forwarded to third parties without prior written consent from **MaaT Pharma.** Reproductions, either in part or in whole, may not be published or copied in any manner, without the explicit written consent of **MaaT Pharma**

5. The expiry date is compatible with the date and time of planned administration
6. The quantity of IMP received

Detailed instructions related to IMP administration, documents to use and contact information in case of any issues are provided in the IMP Manual.

The IMP MaaT033 study drug must be kept out of reach from children and stored safely and securely. It must be stored at +20°C with allowable excursions ranging from +5°C to +25°C, within the expiry date and time stated on the label. The delegated CRO in charge of site management will be responsible for the monitoring of the IMP storage condition on site. Subjects will be instructed on the safe management of study drug within their residential setting.

10.5 IMP PRESCRIPTION AND DELIVERY

The investigator will fill a prescription form and transmit it to the qualified person. Upon prescription, the qualified person will deliver *ad hoc* IMP lots and kits.

Prescription form will be provided in the IMP Manual.

10.6 TREATMENT PROCEDURE

MaaT033 capsules treatment must be considered as any other oral medication. Patient can swallow capsules while drinking water. A maximum of 9 capsules per day is planned, with 3 intakes of 3 capsules. The 3 capsules should be taken in a window of 10 minutes maximum to prevent compliance issues/errors.

Name of the compound: 8P013

Pharmaceutical formulation: capsules

Recommended timing for administration:

Cohort1&2 (1 capsule per day): 1 capsule before breakfast

Cohort 3&4 (3 capsules per day): 3 capsules before breakfast

Cohort 5 (9 capsules per day): 3 capsules before breakfast, 3 before lunch and 3 before dinner

In case of any miss during a given day, the patient may complete the treatment for that day at any time. In other cases (1 day or more of delay), the patient should report the missing capsules and only complete treatment for the day in course.

Patients will use a diary to keep track of their treatment.

Detailed instructions related to the IMP treatment and contact information in case of any issues are provided in the IMP Manual.

10.7 RETRIEVAL AND DESTRUCTION OF UNUSED CAPSULES

A detailed treatment log of the unused IMP by the Pharmacy will be established and countersigned by the Investigator and the Monitoring Team.

-

All documentation of **MaaT Pharma** is PROPRIETARY information and may not be forwarded to third parties without prior written consent from **MaaT Pharma.** Reproductions, either in part or in whole, may not be published or copied in any manner, without the explicit written consent of **MaaT Pharma**

The Investigator/Pharmacist will not destroy the unused IMP unless the Sponsor provides written authorization. In such case, a detailed treatment log of the destroyed Investigational Product will be kept by the Investigator (or the pharmacist) and countersigned by the Investigator and the Monitoring Team.

A potential defect in the quality of IMP may be subject to initiation by the Sponsor of a recall procedure. In this case, the Investigator/Pharmacist will be responsible for promptly addressing any request made by the Sponsor, in order to recall the IMP and eliminate potential hazards.

10.8 CONCOMITANT TREATMENT AND THERAPY

10.8.1 Not recommended concomitant treatment

During the study (see also non-inclusion criteria), the following concomitant medications are not recommended:

1. Antibiotics during the treatment are highly not recommended but will not lead to patient withdrawal (See Appendix 1).
2. Proton Pomp Inhibitor (PPI) are not recommended but will not lead to patient withdrawal.
3. Pre- or Probiotics are highly not recommended but will not lead to patient withdrawal.
4. Gastric dressings and any other treatment that may alter bowel function

10.8.2 Permitted concomitant treatment

Any medications (other than those excluded by the protocol) that are considered necessary for the subjects' well-being and that should not interfere with the trial medication may be given at the Investigator's discretion during the study.

Drugs already being used for cancer relapse prevention are allowed. Moreover, any already used drug for cancer relapse prevention can be exchanged for another one, as long as they belong to the same therapeutic class.

Any relevant treatments which are ongoing before the study and/or prescribed or changed during the study must be recorded on the appropriate pages of the eCRF.

At each visit, the investigator will record on the appropriate pages of the eCRF any change in ongoing treatments.

-

All documentation of **MaaT Pharma** is PROPRIETARY information and may not be forwarded to third parties without prior written consent from **MaaT Pharma.** Reproductions, either in part or in whole, may not be published or copied in any manner, without the explicit written consent of **MaaT Pharma**

11 STUDY ASSESSMENTS PROCEDURES

Patients will be followed and will attend visits according to the calendar study assessment (Table 1 below).

Table 1: Calendar study assessment

		VISITS AND DAYS OF COLLECTION								
		**Screening Phase**	**V1 (D1)**	**Phone call (D2)**	**D4 (+/-2)**	**V2 (D7 +/-2)**	**D10 (+/-2)**	**D14 (+/-2)**	**V3 (Start of consolidation or other cycle) D19 +/-5)**	**V4 (End of consolidation of other cycle study, D44 +/-10)**
Informed consent[1]		X								
Eligibility Criteria		X	X							
Demography		X								
Medical History		X	X							
Physical & clinical exam			X			X			X	X
Vital signs			X			X			X	X
Pregnancy test			X[2]						X	
EBV serology test		X[3]								
SARS-CoV-2 test [11]		X[9]				X			X	X
Patient diary (provision)			X							
MaaT033 intakes			X[4] (posology depending on the cohort)							
Patient diary (return)									X	
Evaluation of the treatment by the patient			X – Once a week or every day from D1 to D7 or 14 depending on the cohort (diary)							
AE / SAE			X (collected from V1 to the end of the study-V4)							
Evaluation of GI symptoms**			X – Collected every day from V1 until V4							
Concomitant treatments			X (record all medications from V1 to the end of the study-V4)							
Laboratory assessments	Hematology[5]		X			X			X	X
	Chemistry[6]		X			X			X	X
	Feces microbiology[7]		X			X			X	X
	Fecal swab[10]		X			X			X	X
	Plasma samples		X			X			X	X

-

All documentation of **MaaT Pharma** is PROPRIETARY information and may not be forwarded to third parties without prior written consent from **MaaT Pharma.** Reproductions, either in part or in whole, may not be published or copied in any manner, without the explicit written consent of **MaaT Pharma**

	PBMC samples		X			X			X	X
Feces collection[8]			X		X	X	X	X	X	X

Abbreviations:	V= Visit; GI= Gastro-Intestinal; AE = Adverse Events; SAE= Serious Adverse Events
[1] Informed consent (ICF)	ICF can be presented and signed to the subject any time from the admission for induction chemotherapy until D1 included. However, study treatment will remain unchanged, and should be started within 2 days after the documented end of neutropenia.
[2] Pregnancy test	If urinary, test is to be done at D1, if blood test, it can be done within 2 days prior to D1.
[3] EBV serology	To be done only if the seropositivity of the EBV is not already documented in patient's medical file.
[4] MaaT033 intakes	It is highly recommended to take the first dose at the hospital at D1.
[5] Blood Hematology	As part of standard of care may content but not limited to RBC, WBC, neutrophils, lymphocytes, monocytes, eosinophils, basophils and platelet count
[6] Blood Chemistry	As part of standard of care may content but not limited to CRP US, Serum glucose, HbA1c, uric acid, urea, ferritin, albumin, pre-albumin
[7] Feces microbiology	As part of standard of care, any coproculture documentation of MDRB carriage
[8] Feces samples	Feces to be collected at home or at the hospital on visit days using a provided device (Fecotainer).<br>D1 sample must be collected prior to the first treatment intake, consequently it can be collected within 2 days prior to V1.<br>Sample at V3 must be collected prior to the new cycle of chemotherapy, consequently it can be collected within 2 days prior to V3<br>At V1, V2, V3 and V4, a sample from the collected stools will be analyzed by a specific central laboratory to assess SARS-CoV-2 presence or absence.
[9] Sars-CoV-2 test	Test must be done within 3 days prior to D1
Fecal swab[10]	In the case where patient does not provide stools on visit days, one fecal swab will be used to do SARS-CoV-2 test
[11] Sars-CoV-2 test	Nasopharyngeal swab, oro-pharyngeal swab, salivary tests or any other validated method used at the clinical investigative site
**	diarrhea, constipation, cramping, nausea, abdominal pain, bloating, gas/ flatulence, transient fever, transient chills

Absolute neutrophil count will be assessed by the physician with local analyses performed within 3 days prior to V1.

-

All documentation of **MaaT Pharma** is PROPRIETARY information and may not be forwarded to third parties without prior written consent from **MaaT Pharma.** Reproductions, either in part or in whole, may not be published or copied in any manner, without the explicit written consent of **MaaT Pharma**

All documentation of **MaaT Pharma** is PROPRIETARY information and may not be forwarded to third parties without prior written consent from **MaaT Pharma.** Reproductions, either in part or in whole, may not be published or copied in any manner, without the explicit written consent of **MaaT Pharma**

11.1 VISIT SCHEDULE – CONDUCT OF THE STUDY

11.1.1 Screening Phase

Patients diagnosed with a hematologic malignancy will be asked if they are willing to participate in this study by the investigating physician and will sign the informed consent form as soon as possible after starting the induction chemotherapy and before any study examination. Only patients with neutropenia recovery (ANC≥0.5*10^9 cells/L) will be included in the study.

This screening period can be performed prior to V1 and can be scheduled at any time from the admission of induction chemotherapy. In this case:

- The physician will collect informed consent form before any study procedures
- The physician will collect demographic information and medical history
- The physician will collect a blood sample for EBV serology if the positivity of this parameter is not already present in patient's medical file.
- The physician will check and document birth control methods (appendix2).
- The physician will check inclusion and non-inclusion criteria;

This screening period can be merged with V1, at the end of aplasia, if the seropositivity of EBV is already known and if a urine pregnancy test is performed instead of a blood test.

Nasopharyngeal SARS-CoV-2 diagnostic test (or any other validated methods: salivary test, oro-pharyngeal test or other) must be performed within 3 days prior to treatment start.

11.1.2 Assessments performed during the entire study

- The safety and tolerability of MaaT033 will be evaluated during the study with the collection of all AEs and SAEs (frequency, grade, relationship) from V1 to V4.
- Vital signs will be monitored at each visit. Vital signs, blood pressure (SBP and DBP) and heart rate in both supine position (after at least 10 minutes rest) and standing position (after 2 minutes), using an automatic sphygmomanometer.
- The compliance of MaaT033 will be evaluated by the patient with a diary to be completed from V1 to V3.
- Results from copro-cultures/hemo-cultures performed during patient's care will be collected in the electronic Case Report Form (eCRF).

11.1.3 Inclusion, Visit1 - D1 - TREATMENT START

Screened patients with confirmed neutropenia recovery will be enrolled in the study after receiving oral and written information and giving informed consent.

The patient will be examined during a consultation by the physician to ensure that the patient fulfills inclusion and non-inclusion criteria.

Patient number will be composed of 9 digits: first 3 digits corresponding to country number, 3 digits corresponding to site number and last 3 digits corresponding to the first number available following that of previous patient included in the trial in the same site.

During this visit:

-

All documentation of **MaaT Pharma** is PROPRIETARY information and may not be forwarded to third parties without prior written consent from **MaaT Pharma.** Reproductions, either in part or in whole, may not be published or copied in any manner, without the explicit written consent of **MaaT Pharma**

- The physician will check inclusion and non-inclusion criteria to confirm patient is still eligible to the study since the screening period;
- Urine or serum pregnancy test will be performed;
- The physician will collect medical history, clinical evaluation (response to chemotherapy) and proceed to a physical exam including collection of vital signs;
- Blood samples (30mL) and fecal swab will be collected (See Table 1 for analysis details);
- The patient will be given a diary for compliance evaluation

If the seropositivity of EBV is already known and if a urine pregnancy test is performed instead of a blood test, screening period can be merged with visit V1, at the end of neutropenia.

The MaaT033 treatment start the day of the inclusion visit, after collection of blood and feces samples for central laboratory. Nasopharyngeal (or any other validated methods: salivary test, oro-pharyngeal test or other) SARS-CoV-2 results must be available before the first intake of MaaT033.

The treatment should be started within 2 days after the documented end of neutropenia and it is highly recommended to take the first dose at the hospital

The patient will receive the adequate quantity of capsules according to the cohort. Capsule containers will be presented in an adequate packaging. Detailed instructions related to IMP administration, documents to use and contact information in case of any issues are provided in the IMP Manual.

The treatment will not be started in case of presence of a toxic megacolon or gastrointestinal perforation, which constitute non-inclusion criteria.

All AE and SAE must be reported in the eCRF.

11.1.4 **Phone call - D2 (+2days)**

A brief short call will be organized by the medical team after the first treatment intake (D2-D4). The aim of this call is to check patient's compliance (and completion of patient diary), to check any safety events observed by the patient and to remind patient to do the stool collection at D4 (+/- 2 days).

This phone call and any events discussed with the patient must be documented in source documentation.

11.1.5 **INTERIM PHASE, Visit 2 - D7 (D5-D9)**

This visit will be performed 7 days (+/-2days) after the MaaT033 treatment start.

During this visit:

- The physician will proceed to a physical exam including collection of vital signs and clinical evaluation
- Blood samples (30mL) and fecal swabs will be collected (See Table 1 for analysis details)
- Evaluation of treatment tolerability, acceptability, compliance and safety
- Nasopharyngeal (or any other validated methods: salivary test, oro-pharyngeal test or other) and fecal SARS-CoV-2 tests will be performed

During this visit, the patient will report to the physician any adverse reaction or severe event occurred since the previous visit.

Regarding cohort4, the treatment will not be continued in case of presence of a toxic megacolon or gastrointestinal perforation, which constitute exclusion criteria.

All AE and SAE must be reported in the eCRF.

-

All documentation of **MaaT Pharma** is PROPRIETARY information and may not be forwarded to third parties without prior written consent from **MaaT Pharma.** Reproductions, either in part or in whole, may not be published or copied in any manner, without the explicit written consent of **MaaT Pharma**

11.1.6 New cycle START, Visit 3 - D19 (D14-D24)

This visit will be performed as Standard of Care or at day 19 (+/-5days).

During this visit:

- The physician will proceed to a physical exam including collection of vital signs and clinical evaluation
- Blood samples (30mL) and fecal swabs will be collected (See Table 1 for analysis details)
- Evaluation of treatment tolerability, acceptability, compliance and safety
- Urine or serum pregnancy test will be performed

Nasopharyngeal (or any other validated methods: salivary test, oro-pharyngeal test or other) and fecal SARS-CoV-2 tests will be performed.

During this visit, the patient will report to the physician any adverse reaction or severe event occurred since the previous visit.

All AE and SAE must be reported in the eCRF.

11.1.7 End of study, Visit 4 - D44 post-inclusion - Cycle STOP

This visit will be performed 44 days (+/-10days) after the MaaT033 treatment start.

During this visit:

- The physician will proceed to a physical exam including collection of vital signs and clinical evaluation
- Blood samples (30mL) and fecal swabs will be collected (See Table 1 for analysis details)
- Evaluation of treatment tolerability, acceptability, compliance and safety

Nasopharyngeal (or any other validated methods: salivary test, oro-pharyngeal test or other) and fecal SARS-CoV-2 tests will be performed.

During this visit, the patient will report to the physician any adverse reaction or severe event occurred since the previous visit. All AE and SAE must be reported in the eCRF.

After V4, patient will be followed like any AML or HR-MDS patient.

In total, 120mL of blood will be collected for each patient during the study.

-

All documentation of **MaaT Pharma** is PROPRIETARY information and may not be forwarded to third parties without prior written consent from **MaaT Pharma.** Reproductions, either in part or in whole, may not be published or copied in any manner, without the explicit written consent of **MaaT Pharma**

11.2 ASSESSMENTS REQUESTED FOR THE STUDY

See Table 1 for calendar of study assessment.

- **Demographics**: gender, date of birth, birth delivery, smoking habits
- **Underlying hematologic malignancy history and assessment**: primary diagnostic date, previous treatments
- **Medical history:** Major past diseases, in the opinion of the investigator. Particular attention will be given to gastrointestinal diseases (Crohn's disease, ulcerative colitis, inflammatory bowel disease, colorectal cancer…)
- **Concomitant diseases and medications**: Chronic diseases and long-term treatments must be recorded. Record if medication is continuing during the treatment in the study.
- **Physical exam**: height, weight, vital signs: temperature, blood pressure, heart rate
- **Evaluation of MaaT033 compliance by the patient** with a diary.
- **Evaluation of induction chemotherapy response at inclusion and throughout the study**
- **Collection of AEs and SAEs (from the date of informed consent)**

-

All documentation of **MaaT Pharma** is PROPRIETARY information and may not be forwarded to third parties without prior written consent from **MaaT Pharma.** Reproductions, either in part or in whole, may not be published or copied in any manner, without the explicit written consent of **MaaT Pharma**

11.3 BIOLOGICAL ANALYSES REQUESTED FOR THE STUDY

11.3.1 Blood analysis

Table 2: List of blood assessments

Hematology	Clinical Chemistry	Immunology/markers
Red blood cell (RBC) count	hs-CRP	Multiparameter assay on plasma
White blood cell (WBC) count	Ferritin	Reg3α
Neutrophils	Uric acid	ST2
Lymphocytes	Urea	Immunomonitoring on Peripheral Blood Mononuclear Cells (PBMC)
Monocytes	Albumin	metagenomic on PBMC
Eosinophils	Pre-albumin	
Basophils	Total protein	
Platelet count	Triglycerids	
Hemoglobin	Lactate dehydrogenase (LDH)	
Hematocrit	Cholesterol	
	Total and direct bilirubin	
	Alanine transaminase (ALT)	
	Aspartate transaminase (AST)	
	Gamma Glutamyl Transpeptidase (GGT)	
	Alkaline phosphatase	
	Serum Glucose	
	Hb1Ac	

Blood hematology and clinical chemistry analyses will be performed by local hospital laboratories.

Blood collection for PBMC extraction and subsequent storage until analysis at the end of the study, will be performed by local laboratories able to perform this procedure.

Results from blood samples and feces cultures performed during patient's care will be collected in the eCRF. All lab results must be documented in the eCRF before the end of study. Additionally, reference ranges must be provided to the Sponsor or its designee.

-

All documentation of **MaaT Pharma** is PROPRIETARY information and may not be forwarded to third parties without prior written consent from **MaaT Pharma.** Reproductions, either in part or in whole, may not be published or copied in any manner, without the explicit written consent of **MaaT Pharma**

11.3.2 Feces analysis

Table 3: List of feces assessments

Feces microbiota analysis	Feces biochemistry
Bacterial DNA sequencing (metagenomics)	Neopterin
	Zonulin
	IgA
	Biliary acids
	Short Chain Fatty Acids (SCFA)

Feces analysis will be performed in a selected central laboratory.

11.3.3 Management of samples

Table 4: Samples Management

	Blood immunology	Feces	Fecal swabs
**Sample size**	30 mL	at least 10g / 20mL	2 swabs
**Material**	PBMC: 2x9ml Heparin tube<br>Plasma: 2x6ml EDTA tube	Feces container<br>(Fecotainer®)	Rectal swabs<br>(dry type)
**Processing**	Local laboratory	Central laboratory	Local laboratory
**Storage location and conditions**	PBMC extraction within 2 to 6 hours<br>Storage -80°C and liquid nitrogen on site	Biochemistry:<br>storage -80°C until analysis	Metagenomics:<br>storage -80°C until analysis
**Analysis (end of study)**	In dedicated laboratory	In dedicated laboratory	In dedicated laboratory

11.4 TERMINATION

11.4.1 Patient study withdrawal

Patients may be discontinued from the study in following situations:

1. Informed consent withdrawal by the patient or by the patient's legal representative;
2. Investigator's decision according but not exclusive to the following criteria:
   - Retroactive failure to fulfil inclusion/exclusion criteria;
   - Loss of contact, relocation. For lost to follow-up patients, investigator must make all possible attempts to reach the patient (phone calls, letters) with documented evidence.
   - New medical conditions not allowing for continuation of the protocol-conform treatment;

All documentation of **MaaT Pharma** is PROPRIETARY information and may not be forwarded to third parties without prior written consent from **MaaT Pharma.** Reproductions, either in part or in whole, may not be published or copied in any manner, without the explicit written consent of **MaaT Pharma**

- Occurrence of adverse events (such as allergic reactions to study drug, pregnancy, laboratory abnormalities) leading to substantial changes in the individual risk-benefit considerations that suggest a discontinuation of study drug

3. Any adverse event leading to interruption or contraindication of the MaaT033 treatment. Patients who stop treatment due to AE related to the procedure will continue to be monitored for safety follow-up. The reason for procedure stop must be documented (*e.g.,* parenteral nutrition, impossibility to swallow).
4. Severe noncompliance to the protocol as judged by the investigator and/or MaaT Pharma.

In case of patient's withdrawal from the study, irrespective of the reason, an early study termination visit must be performed and recorded by the investigator in the appropriate forms of the eCRF and in the subject's medical records when considered as confirmed (*e.g.,* date of and reason for withdrawal).

Subjects who have been withdrawn from the study cannot be re-included in the study.

11.4.2 **End of study treatment**

Patients would stop treatments before the end (day 7 or 14) for the following reasons:

- Life-threatening SAE related to MaaT033

- Medical decision: for instance, disease progression due to treatment failure according to the investigator's judgement

Patients who stop treatments from the study for clinical / symptomatic deterioration or disease progression will continue to be monitored for safety follow-up, survival and disease until day 44. The reason for and the date of end of treatment must be documented.

If the treatment discontinuation is related to an AE, the subject will be followed up to recovery or stabilization of the AE, whichever comes last. All definitive treatment discontinuation should be recorded by the Investigator in the appropriate pages when considered as confirmed.

11.4.3 **Maintenance use**

A total of 7 or 14 days of treatment are planned. However, the duration of treatment will depend on patient's tolerance.

11.5 PREMATURE DISCONTINUATION OF THE STUDY OR PREMATURE CLOSE-OUT OF A SITE

11.5.1 **Decided by the Sponsor in the following cases:**

The trial can be prematurely stopped (definitely or temporarily) when:

The treatment is considered as too noxious to continue with further clinical investigations

If the information on the product leads to doubt as to the benefit/risk ratio;

Occurrence of new fact that can modified modify MaaT Pharma / Competent Authority approval on the trial, or for any unethical reason

MaaT Pharma decision to stop product development

-

All documentation of **MaaT Pharma** is PROPRIETARY information and may not be forwarded to third parties without prior written consent from **MaaT Pharma.** Reproductions, either in part or in whole, may not be published or copied in any manner, without the explicit written consent of **MaaT Pharma**

In case of any reason motivating such withdrawal, the Investigator should promptly inform the patients, ensure appropriate therapy and follow-up, and complete the CRF with all available data at the time of trial arrest.

Trial withdrawal with the reason will be declared to Competent Authority (and EC if applicable) in accordance with local requirements.

If the Investigator has received from the Sponsor means and information necessary to perform the Clinical Trial and has not included any subject after a reasonable period of time mutually agreed upon;

In the event of breach by the Investigator of a fundamental obligation under this agreement, including but not limited to breach of the Clinical Trial Protocol, breach of the applicable laws and regulations or breach of the ICH guidelines for Good Clinical Practice;

If the total number of subjects are included earlier than expected;

In any case the Sponsor will notify the Investigator of its decision by written notice.

11.5.2 **Decided by the Investigator**

The Investigator must notify (30 days' prior notice) the Sponsor of his/her decision and give the reason in writing.

In all cases (decided by the Sponsor or by the Investigator), the appropriate Ethics Committee(s) (IRB/IEC) and Health Authorities should be informed according to applicable regulatory requirements.

-

All documentation of **MaaT Pharma** is PROPRIETARY information and may not be forwarded to third parties without prior written consent from **MaaT Pharma.** Reproductions, either in part or in whole, may not be published or copied in any manner, without the explicit written consent of **MaaT Pharma**

12 SAFETY

All events will be managed and reported in compliance with all applicable regulations and included in the final Clinical Study Report.

When it is deemed necessary, the Investigator should consider reporting the cases and completing an SAE form and referring to an appropriate specialist to ensure proper characterization and documentation of the event as well as to ensure appropriate management.

12.1.1 Definitions (ICH / GCP)

An **Adverse Event** is any untoward medical occurrence in a subject or clinical investigation subject administered a pharmaceutical product and which does not necessarily have to have a causal relationship with this treatment.

A priori, efficacy endpoints as specified in the protocol will not be considered as AEs except if, because of the course or severity or any other features of such events, the Investigator, according to his/her best medical judgment, considers these events as exceptional in this medical condition.

If a subject is medicated or receives other non-study therapy for an abnormal Clinically Significant laboratory evaluation (unless this is standard of care or for a pre-existing condition), this will be recorded as an AE.

An **Adverse Drug Reaction** is any noxious and unintended response to a medicinal product related to any dose administered. The phrase response to a medicinal product means that a causal relationship between a medicinal product and an adverse event is at least a reasonable possibility, i.e. the relationship cannot be ruled out.

A **Serious Adverse Event** is any untoward medical occurrence that at any dose:

1 Results in death or;

2 Is life-threatening or;

Note: The term "life-threatening" in the definition of "serious" refers to an event in which the subject was at risk of death at the time of the event; it does not refer to an event which hypothetically might have caused death if it were more severe.

3 Requires subject hospitalization or prolongation of existing hospitalization or;

Note: Planned hospitalization before inclusion and procedures related to AML or HR-MSD routine management will not be considered as SAE

4 Results in persistent or significant disability/incapacity or;

5 Is a congenital anomaly/birth defect;

6 Is a medically important event:
Medical and scientific judgment should be exercised in deciding whether expedited reporting is appropriate in other situations, such as important medical events that may not be immediately life-threatening or result in death or hospitalization but may jeopardize the subject or may require intervention to prevent one of the other outcomes listed in the definition above.

-

All documentation of **MaaT Pharma** is PROPRIETARY information and may not be forwarded to third parties without prior written consent from **MaaT Pharma.** Reproductions, either in part or in whole, may not be published or copied in any manner, without the explicit written consent of **MaaT Pharma**

Unexpected Adverse Drug Reaction:

An adverse reaction, the nature, or severity of which is not consistent with the applicable product information (e.g. Investigator's Brochure for an unapproved investigational product or package insert/summary of product characteristics for an approved product).

The Investigator should ensure that adequate medical care is provided to the subject for any adverse event.

12.1.2 Collection, recording and reporting of adverse events

In this study, all events have to be reported from V1 to V4 and a particular attention should be given to events related to infectious disorders. In case of sepsis that may be related to the IMP according to the investigator, any sample of interest must be collected and analyzed for the identification of the pathogen – according to the SOP "Sepsis" that MaaT Pharma has for these instances.

Following notification from the investigator, the Sponsor takes responsibility for reporting the suspected unexpected serious adverse reactions (SUSAR) and any follow-up information to EMA's EudraVigilance and local EU Health Authorities as necessary for the patient involved according to the required timelines.

In particular, the sponsor will inform the investigators of all sites of serious and unexpected adverse events that are considered to be possibly or probably related to the administered IMP.

All SAEs designed as "not related" to the IMP, will be reported to the authorities in Development Safety Update Report (DSUR) annually, if appropriate.

All COVID-19 infections must be reported as an SAE. The Sponsor takes responsibility for reporting this type of infection immediately to ANSM as a new fact.

Moreover, any other new information that might materially influence the benefit-risk assessment of the IMP or that would be sufficient to consider changes in IMP administration or in the overall conduct of the clinical study, must be reported to the authority.

In accordance with the ICH GCP guidelines, the sponsor will inform the investigators of findings that could affect adversely the safety of the subjects, impact the conduct of the study, or alter the authority approval to continue the study.

12.1.3 Obligation of the Investigator regarding safety reporting

12.1.3.1 <u>Adverse Events</u>

All Adverse Events regardless of seriousness or relationship to Investigational Product, spanning from the first visit planned in the Clinical Trial Protocol (V1), up to the last visit planned in the protocol (V4), are to be recorded on the corresponding page(s) included in the eCRF.

Whenever possible, symptoms should be grouped as a single syndrome or diagnosis. The Investigator should specify the date of onset, intensity (mild, moderate, severe), action taken with respect to Investigational Product, corrective treatment/therapy given, additional investigations performed, outcome and his/her opinion as to whether there is a reasonable possibility that the Adverse Event was caused by the Investigational Product.

-

All documentation of **MaaT Pharma** is PROPRIETARY information and may not be forwarded to third parties without prior written consent from **MaaT Pharma.** Reproductions, either in part or in whole, may not be published or copied in any manner, without the explicit written consent of **MaaT Pharma**

12.1.3.2 Serious Adverse Events

In the case of a Serious Adverse Event the Investigator must immediately:

1 ENTER (within 24 hours) the information related to the Serious Adverse Event in the appropriate screens of the e-CRF; the system will automatically send the notification to the pharmacovigilance officer after approval of the Investigator within the e-CRF or after a standard delay. A back-up plan is in place (using paper flow) when the e-CRF system does not work,

2 In case of eCRF system failure, FILL in (within 24 hours) the information related to the Serious Adverse Event in the appropriate Serious Adverse Event Form and fax it immediately to the pharmacovigilance officer (Vigipharm subcontractor).

3 SEND (preferably by fax or e-mail) the photocopy of all examinations carried out and the dates on which these examinations were performed, to the pharmacovigilance officer.

Care should be taken to ensure that the subject's identity is protected and the subject's identifiers in the Clinical Trial are properly mentioned on any copy of source document provided to the Sponsor. For laboratory results, include the laboratory normal ranges.

All further data updates should be recorded in the eCRF as appropriate, and further documentation as well as additional information (for lab data, concomitant medication, subject status) should be sent (by fax or e-mail) to the Monitoring Team within 24 hours of knowledge. In addition, any effort should be made to further document each Serious Adverse Event that is fatal or life threatening within the week (7 days) following initial notification.

SAE EMAIL : maatpharma@vigipharm.fr

SAE FAX N° +33 (0) 467 107 253

The Investigator must comply with any applicable site-specific requirements related to the reporting of SAEs) involving his/her subjects to the Ethics Committee/Institutional Review Board (EC/IRB) that approved the trial.

Investigator should ensure that adequate medical care is provided to the subject for any adverse event.

All SAE must be followed until the subject has recovered, stabilized, recovered with sequelae or died. The Investigator must forward follow-up information on SAE to MaaT Pharma within 5 days this information is obtained. SAE Follow-up report should be faxed accordingly to SAE EMAIL: maatpharma@vigipharm.fr and SAE FAX N° +33 (0) 467 107 253

12.1.3.3 Adverse Events of Special Interest

The events of interest during MaaT033 treatment and follow up are AEs related to**:**

- Infections and infestations
- GI disorders

12.1.3.4 Laboratory tests abnormalities

Any abnormal laboratory value should be reported as an AE, only if the following applies:

- If the abnormal value is associated with clinical symptoms, or
- If the abnormal value is considered medically significant by the Investigator, or
- If the abnormal value results in change of IMP administration (*i.e.* change in schedule, dose)
- If the abnormal value requires change in concomitant treatments or diagnostic evaluation to assess the risk for the patient.

-

All documentation of **MaaT Pharma** is PROPRIETARY information and may not be forwarded to third parties without prior written consent from **MaaT Pharma.** Reproductions, either in part or in whole, may not be published or copied in any manner, without the explicit written consent of **MaaT Pharma**

Any borderline value can be ultimately assessed by the PI to be on one or the other side of the corresponding threshold – taking a conservative approach and carefully considering the medical history and previous lab results of the patient.

12.1.3.5 Recording procedure

Any event meeting the definition of an AE defined below must be collected and reported in the patient's CRF throughout study duration i.e., from the first trial related activity after the subject has signed the informed consent and until the end of the study.

All AEs must be followed until resolution or up to the end of the protocol. Serious adverse events that are life threatening, or leading to death, will be recorded throughout the study duration.

If there are several events and different symptoms combined into a main AE, then the only main AE (with a properly defined diagnosis according to code is always preferable) should be recorded in the CRF. Where there is no link between different clinical symptoms occurring at the same time, each sign should be recorded as a separate AE.

Occurrence and follow-up of AE should be assessed at each visit, and essential data listed hereafter should be recorded in the patient's CRF and patient's medical file as well:

- the severity or grade of the adverse event
- the causality: determination of whether an adverse event is reasonably related to the investigational treatment or procedure, and/or other agents are suspected of causing the adverse event
- the action(s) taken regarding investigational product (continued / interrupted / stopped)
- the corrective treatment if any
- the event outcome
- the seriousness of the event

12.1.3.6 Intensity / AE grading

Grade is used to denote the severity of the adverse event. The NCI-CTCAE v5.0, will be used as reference for AE grading in this trial. If the term does NOT appear in the CTCAE, the AE is graded using the following categories:

- **Mild (Grade 1):** minor; no specific medical intervention; asymptomatic laboratory findings only, radiographic only; marginal clinical relevance

- **Moderate (Grade 2):** minimal intervention; local intervention; noninvasive intervention (packing, cautery),

- **Severe (Grade 3):** significant symptoms requiring hospitalization or invasive intervention; transfusion; elective interventional radiological procedure; therapeutic endoscopy or operation). The term “severe” is often used to describe the intensity (severity) of a specific event. This is not the same as “serious,” which is based on patient/event outcome or action criteria (see section 11.2).

- **Life-threatening or disabling (Grade 4):** complicated by acute, life-threatening metabolic or cardiovascular complications such as circulatory failure, hemorrhage sepsis. Life-threatening physiologic consequences; need for intensive care or emergent invasive procedure; emergent interventional radiological procedure, therapeutic endoscopy or surgery.

- **Death or Fatal** (Grade 5)

All documentation of **MaaT Pharma** is PROPRIETARY information and may not be forwarded to third parties without prior written consent from **MaaT Pharma.** Reproductions, either in part or in whole, may not be published or copied in any manner, without the explicit written consent of **MaaT Pharma**

12.1.3.7 Causality assessment

Attribution of causality is the determination of whether an adverse event is related to a medical treatment or procedure; categories are:

- Certain: the AE is clearly related to the treatment or procedure.
- Probable: the AE is likely related to the treatment or procedure, there are good reasons and sufficient documentation to assume a causal relationship.
- Possible: the AE is may be related to the treatment or procedure; a causal relationship is conceivable and cannot be dismissed.
- Unlikely: AE or laboratory test abnormality, with a time to drug that makes a relationship improbable (but not impossible). Diseases or other drugs provide plausible explanations
- Not related: the AE is clearly NOT related to the treatment or procedure. The event is most likely related to etiology other than the treatment or procedure.

Causality will be grouped by categories:

- Reasonable possibility: where the AE is clearly (certain), likely (probable) or may be (possible) related to the treatment or procedure. Such events would be considered adverse reactions.
- No reasonable possibility: where the AE is clearly NOT (not related) or doubtfully (unlikely) related to the treatment or procedure.

12.1.3.8 Outcome

The event outcome is mandatory and should be recorded as soon as it gets resolved, where appropriate when the subject has completed the protocol at the latest. The outcome should be one of the following:

- **Recovering:** the AE is still ongoing with an improvement
- **Not yet recovered**: the AE is still ongoing when the subject has completed the protocol.
- **Recovered**: fully recovered or by medical or surgical treatment the condition has returned to baseline level.
- **Recovered with sequelae**: as a result of the AE, the subject suffered persistent and significant disability/incapacity (e.g. became blind, deaf, paralyzed). Any AE recovered with sequelae should be declared as an SAE.
- **Fatal**: any AE resulting in death should be declared as an SAE

12.1.3.9 Follow-up

The Investigator should take all appropriate measures to ensure the safety of the subjects, notably he/she should follow up the outcome of any Adverse Events (clinical signs, laboratory values or other, etc.) until the return to normal or consolidation of the subject's condition;

In case of any Serious Adverse Event, the subject must be followed up until clinical recovery is complete and laboratory results have returned to normal, or until progression has been stabilized. This may imply that

All documentation of **MaaT Pharma** is PROPRIETARY information and may not be forwarded to third parties without prior written consent from **MaaT Pharma.** Reproductions, either in part or in whole, may not be published or copied in any manner, without the explicit written consent of **MaaT Pharma**

follow-up will continue after the subject has left the Clinical Trial and that additional investigations may be requested by the pharmacovigilance officer.

In case of any Serious Adverse Event brought to the attention of the Investigator at any time after the clinical trial with a reasonable possibility, this should be reported to the pharmacovigilance officer.

12.1.3.10 Pregnancy

Pregnancy will be recorded as an AE in all cases. It will be qualified as an SAE only if it fulfills SAE criteria.

In the event of pregnancy, the Investigator inform immediately (i.e. within 24 hours), even not fulfilling a seriousness criterion using the corresponding pages in the CRF (to be sent) or the screens in the e-CRF, following the same process as described for the Serious Adverse Events.

Follow-up of the pregnancy will be mandatory until the outcome has been determined.

12.1.4 **Safety Committee (SC)**

The tolerability of the study **will be evaluated for every patient by the Committee, once for each cohort.**

The events of interest during MaaT033 treatment are**:**
- Infections and infestations
- GI disorders

The responsibilities of the committee and its members are:

- To review tolerability of MaaT033 different dose regimen at the end of different cohort follow up (see design of the study) and make recommendations to continue, modify or terminate the study
- To ensure the ongoing safety for study subjects
- To review all study documents provided by the sponsor
- To be the referee for the imputability of adverse reactions on behalf of sponsor

12.1.4.1 Tolerability consideration:

Investigators will be trained regarding the mode of action of the investigational medicinal product and to be particularly attentive to any signs of toxicity.

Dose Limiting Toxicity (DLT): Based on the literature and our preclinical data, no particular toxicity is expected. Toxicity events will be defined as any at least possibly related adverse event grade ≥3 based on CTCAE v5.0 criteria. Due to the specific therapeutic protocol that blood cancerpatients usually follow, the follow-up of patients in term of DLT is set from V1 (end of neutropenia) to V3 (start of a new chemotherapy cycle, or consolidation). Nevertheless, there are some anatomical or physiological system events that will be considered as expected in the context of patients with AML or HR-MSD, and thus not related to the treatment. Such events will only be considered DLT when grade>3:

- Blood and lymphatic system disorders (DLT when grade4 or 5)
- Immune system disorders (DLT when grade4 or 5)

Depending on the number of DLTs, the dose is either escalated (0 DLTs) or de‐escalated (≥2 DLTs). Due to our specific drug activity assessment goals, cohorts 2 to 5 will systematically be expanded with 3 extra patients (0 or 1 DLTs) each.

All documentation of **MaaT Pharma** is PROPRIETARY information and may not be forwarded to third parties without prior written consent from **MaaT Pharma.** Reproductions, either in part or in whole, may not be published or copied in any manner, without the explicit written consent of **MaaT Pharma**

12.1.4.2 Cohort Review Meetings:

Ad-hoc meetings can be called at any moment if needed for Safety consideration (DSMB).

The dose-escalation process will be monitored by a **cohort review committee (DSMB, see corresponding chapter 8.2.2. for the dose escalation rules)**.

A cohort review meeting will be organized once all patients at a specific dose level have completed the V1 to V3 DLT period to decide regarding dose escalation. A cohort review meeting will also be organized as soon as DLTs are observed in 2 or more patients to decide about stopping the dose escalation and confirming the Maximum Tolerated Dose (MTD) has been reached:

Cohort1: 3 first patients in this cohort will allow the assessment of a potential unexpected safety signal and will confirm feasibility of the procedure. The CRC will review patients after treatment completion of the 3 patients. Safety, tolerability and DLT events between V1 and V2 will be evaluated to take the decision on dose escalation.

Cohorts 2&3: These cohorts present a more classical posology (daily treatment of 1 and 3 capsules respectively) in the microbiome capsule category (see section introduction). The CRC will review patient tolerability after treatment completion of the 6 patients of the evaluated cohort, completed with follow up information from of the previous cohorts. Tolerability and DLT events between V1 and V3 will be evaluated to take the decision on dose escalation.

Cohorts 4&5: No more dose regimen escalation are expected. Tolerabiliy of those patients will of course be closely monitored. Because cohort4 will first be completed, a SC will validate the step up to cohort5.

The focus of this meeting will be to review the overall patients' safety and to ensure all DLTs have been identified to decide escalation to the next dose level, or to confirm if the MTD has been reached.

At the time of the decision to escalate the dose, the cohort review committee may recommend one or more of the following with the aim of establishing an MTD and/or enhancing potential safety and tolerability:

- That additional medication is used
- That specific measures are taken in the treatment of side effects based on AEs and building upon the cumulated experience in their management
- That caution is exercised in patients presenting with conditions that may exacerbate any observed toxicities

The cohort review committee may permit enrollment of additional patients in any cohort to ensure that there is the required number of patients with evaluable DLTs at each dose level cohort.

The cohort review committee may review and delay enrollment into the next dose cohort based on observed toxicities.

12.1.4.3 Recommended Phase 2 Dose RP2D

Once the MTD or MED is determined, or once the highest dose has been reached without declaring an MTD, the cohort review committee will select the recommended dose for the following studies with MaaT033 (recommended Phase 2 dose; RP2D).

RP2D usually is the highest dose with a pre‐specified DLT rate. In this study, this dose will be the MTD, a dose below the MTD, or any other dose suggested upon clinical evaluation or all available safety, activity and efficacy parameters.

In case an RP2D is selected that is not the MTD, a minimum of 6 patients will need to be treated and followed-up for 7 days at this dose level (with a maximum of 1 DLT observed), before declaring it as the RP2D.

-

All documentation of **MaaT Pharma** is PROPRIETARY information and may not be forwarded to third parties without prior written consent from **MaaT Pharma.** Reproductions, either in part or in whole, may not be published or copied in any manner, without the explicit written consent of **MaaT Pharma**

13 DATA MANAGEMENT

13.1 DEFINITION OF SOURCE DATA

All evaluations that are reported in the electronic Case Report Form (eCRF) must be supported by appropriately signed identified source documentation related.

13.2 SOURCE DOCUMENT REQUIREMENTS

According to the guidelines on GCP, the Monitoring Team must check the CRF / eCRF entries against the source documents, except for the pre-identified source, data directly recorded/enclosed in/to the Case Report Form. The Informed Consent Form will include a statement by which the subject allows the Sponsor's duly authorized personnel and the regulatory authorities to have direct access to source data which supports the data on the Case Report Forms (e.g. subject's medical file, appointment books, original laboratory records, etc.). These personnel, bound by professional secrecy, will not disclose any personal identity or personal medical information.

13.3 USE AND COMPLETION OF CASE REPORT FORMS (CRFS) AND ADDITIONAL REQUEST

13.3.1 Data collection

All clinical data will be reported electronically by the Investigator or authorized designee on a web-based electronic Case Report Form (eCRF). This eCRF is specifically designed for the study and developed by the Data Management Department of Eurofins Optimed using LifeSphere EDC® 5.0.2. or higher, a validated Electronic Records/Electronic Signature-compliant (21 CFR Part 11) application.

Should a correction be made, the corrected information will be entered in the eCRF and the initial information will be tracked in the audit trail.

13.3.2 Responsibilities

The Investigator or authorized designee is responsible for the timeliness, completeness, and accuracy of all observations and other data pertinent to the clinical investigation in the eCRFs. The Investigator will ensure that all data are entered promptly (within 4 days) after the evaluation has occurred, in accordance with source documents and specific instructions accompanying the eCRFs, designed specifically for the study. The Data Management Department of Eurofins Optimed will provide all tools, instructions, and training necessary to complete the eCRF, and each user will be issued a unique username and password. The data management of Eurofins Optimed will be responsible for data processing, in accordance with the CRO data management procedures.

All documentation of **MaaT Pharma** is PROPRIETARY information and may not be forwarded to third parties without prior written consent from **MaaT Pharma.** Reproductions, either in part or in whole, may not be published or copied in any manner, without the explicit written consent of **MaaT Pharma**

13.4 DATA MANAGEMENT

During the study, through regular data collection and monitoring, clinical data reported in the eCRFs will be integrated into the clinical database. Computerized logic and/or consistency checks will be systematically applied in order to detect errors or omissions. Queries will be generated and submitted through the electronic data capture (EDC) system to the investigator sites for resolution (queries should be answered within 10 days). Correction will be made either automatically from the immediate completion or following the review of the data during the Eurofins Optimed monitoring. An audit trail, which will be initiated at the time of the first data entry, allows tracking all modifications. The Data Management Department of Eurofins Optimed may generate additional requests to which the Investigator must respond electronically by confirming or modifying the data questioned. The requests with their responses will be implemented to the eCRFs. Each step of this process will be monitored through the implementation of individual passwords and regular backups to maintain appropriate database access and to ensure database integrity. When eCRFs are complete and all queries have been answered, the Investigator has to sign the eCRFs. Then eCRFs are locked and no modification is possible anymore. After integration of all corrections in the complete set of data, the database will be locked and saved before being released for statistical analysis. After database lock, a Patient Data Report (PDR) that consists of the printing out an entire casebook for a subject will be generated for each subject in .pdf format. PDRs are sent to site and sponsor via secured exchange platform.

-

All documentation of **MaaT Pharma** is PROPRIETARY information and may not be forwarded to third parties without prior written consent from **MaaT Pharma.** Reproductions, either in part or in whole, may not be published or copied in any manner, without the explicit written consent of **MaaT Pharma**

14 STATISTICAL CONSIDERATIONS

The material of this section is the basis of the Statistical Analysis Plan for the study. This plan may be revised during the study to accommodate Clinical Trial Amendment and to make change to adapt unexpected issue in study execution and data that affect planned analyses. These revisions will be based on review of the study and data, and a final plan will be issued before database lock.

14.1 GENERAL CONSIDERATIONS

This is a single arm multicenter study aiming at evaluating the safety of a freeze dried, full ecosystem, encapsulated full-ecosystem, intestinal microbiota biotherapeutic product (MaaT033).

14.1.1 Endpoints

Detailed definitions of endpoints are given in section 8.3.1.

The primary evaluation criterion is the proportion of patients having experienced drug related adverse events for the determination of a recommended phase 2 dose (RP2D).

Secondary endpoints are:

- Evaluation of microbiota reconstitution post MaaT033
  - Alpha-diversity: Simpson index, Shannon index, Richness index
  - Similarities between samples: Bray-Curtis/Jaccard/UniFrac distances indexes, Sorensen index, Pearson index, Spearman index
- Duration of overall response on the hematologic malignancy (blasts)
- Compliance to allogeneic MaaT033 treatment
- Acceptability of MaaT033 by the patient
- Number of patients receiving the full treatments
- Evaluation of MaaT033 activity and/or impact on:
  - Number and percentage of patients having experienced at least one infection requiring antibiotic/antiviral/antifungal therapy after MaaT033 treatment start
  - Number and percentage of patients having experienced at least one microbiologically documented infection requiring antibiotic/antiviral/antifungal therapy after MaaT033 treatment start
  - Events potentially related to GI infection (fever, diarrhea, abdominal pain, bloating)
  - Fecal resistome and MDRB carriage
  - Evaluation of stools: Bristol stool chart

Exploratory endpoints encompass
- Assessment of a microbiota signature
- Impact of MaaT033 on the immune system

The final main analysis will be performed when all enrolled patients have completed treatment and follow-up period (28 days). Complementary analysis on safety and 12 months' survival follow up will be updated when all patients have had the opportunity to be followed for 12 months.

14.1.2 Handling of missing data

Missing data will not be replaced.

-

All documentation of **MaaT Pharma** is PROPRIETARY information and may not be forwarded to third parties without prior written consent from **MaaT Pharma.** Reproductions, either in part or in whole, may not be published or copied in any manner, without the explicit written consent of **MaaT Pharma**

14.1.3 Type 1 error

The analysis will mainly be descriptive. The 1 error rate will be set at 0.05 and therefore two-sided 95% confidence intervals will be calculated.

14.2 ANALYSIS POPULATIONS

- Screened patients: all patients having signed an informed consent form
- Enrolled patients: all patients having been enrolled at V1
- Intent to treat (ITT) population: enrolled patients
- Treated patients: all enrolled patients having undergone at least one capsule intake (safety set)
- Per protocol (PP) population: all patients belonging to the ITT population with at least 60% of treatment and completion of V1, V2 and V3, with no major protocol violations (PP set)

14.3 STATISTICAL METHODS

14.3.1 Planned analyses

One primary analysis is planned after the completion of the last patient last visit (LPLV)

14.3.2 Descriptive statistics

Continuous variables will be described by the mean, standard deviation, minimum and maximum values, median, first and third quartiles. Categorical variables will be described by the number and percentage of each modality.

14.3.3 Demographics and medical history

Demographic data will be described appropriately. Medical history will be coded using the last version of the MedDRA dictionary and will be described according to the system organ and preferred term. Underlying hematologic malignancy will be described. The analysis will be conducted in the safety set.

14.3.4 Treatment exposure

Exposure to MaaT033 will be described by the number of capsules taken by each patient (categorical variable). Concomitant treatments will be coded with the last version of the WHODRUGS dictionary (WHO-DD) and described according to the ATC code and the drug name or preferred terms. The analysis will be conducted in the safety set.

14.3.5 Analysis of the main criterion

The number and percentage of patients having experienced AEs between V1 and V4 will be described. Relation of AEs to the drug treatment will be assess by the safety committee in a final review. The committee will then provide a conclusive decision on MaaT033 safety.

-

All documentation of **MaaT Pharma** is PROPRIETARY information and may not be forwarded to third parties without prior written consent from **MaaT Pharma.** Reproductions, either in part or in whole, may not be published or copied in any manner, without the explicit written consent of **MaaT Pharma**

14.3.6 Analysis of the secondary criteria

Descriptive statistics will be performed on secondary endpoints, unless otherwise specified in the statistical analysis plan.

14.3.7 Analysis of exploratory endpoints

Descriptive statistics will be performed, based on the development of a microbiota signature.
Results of flow cytometry and multiparameter assay for cytokines will be statistically described to assess the impact of MaaT033 full ecosystem intestinal microbiota biotherapeutic on the immune system.

14.3.8 Interim analyses

No interim analysis is planned.

14.3.9 Sample size justification

Data on safety of MaaT033 in hematologic malignancies are still scarce. As the objective of this study is to assess the safety profile of MaaT033 in patients post intensive chemotherapy for the determination of a recommended phase 2 dose (RP2D), no formal sample size calculation has been made.

21 patients will be allowed estimating various proportions with the following two-sided 95%CI

Proportion	Two-sided 95%CI
10%	[2.1%; 26.5%]
20%	[7.7%;38.6%]
30%	[14.7%;49.4%]
40%	[22.7%;59.4%]
50%	[31.3%;68.7%]
60%	[40.6%;77.3%]
70%	[50.6%;85.3%]
80%	[61.4%;92.3%]
90%	[73.5%;97.9%]

-

All documentation of **MaaT Pharma** is PROPRIETARY information and may not be forwarded to third parties without prior written consent from **MaaT Pharma.** Reproductions, either in part or in whole, may not be published or copied in any manner, without the explicit written consent of **MaaT Pharma**

15 ETHICAL AND REGULATORY STANDARDS

15.1 ETHICAL PRINCIPLES

This Clinical Trial will be conducted in accordance with the principles laid down by the 18th World Medical Assembly (Helsinki, 1964) and all applicable amendments laid down by the World Medical Assemblies, and the International Conference of Harmonization (ICH) guidelines for Good Clinical Practice (GCP).

15.2 LAWS AND REGULATIONS

This Clinical Trial will be conducted in compliance with all international laws and regulations, and national laws and regulations of the country(ies) in which the Clinical Trial is performed, as well as any applicable guidelines.

15.3 INFORMED CONSENT

The Investigator (according to applicable regulatory requirements), or a person designated by the Investigator, and under the Investigator's responsibility, should fully inform the Subject or a Subject's legally acceptable representative of all pertinent aspects of the Clinical Trial including the written information giving approval/favorable opinion by the Ethics Committee (IRB/IEC). All participants should be informed to the fullest extent possible about the study, in language and terms they are able to understand.

Prior to a subject's participation in the Clinical Trial, the written Informed Consent Form should be signed, name filled in and personally dated by the subject or by the subject's legally acceptable representative, and by the person who conducted the informed consent discussion. A copy of the signed and dated written Informed Consent Form will be provided to the subject.

The Informed Consent Form used by the Investigator for obtaining the subject's informed consent must be reviewed and approved by the Sponsor prior to submission to the appropriate Ethics Committee (IRB/IEC) for approval/favorable opinion.

15.4 INSTITUTIONAL REVIEW BOARD/INDEPENDENT ETHICS COMMITTEE (IRB/IEC)

As required by local regulation, the Investigator or the Sponsor must submit this Clinical Trial Protocol to the appropriate Ethics Committee (IRB/IEC), and is required to forward to the respective other party a copy of the written and dated approval/favorable opinion signed by the Chairman with Ethics Committee (IRB/IEC) composition.

The Clinical Trial (study number, Clinical Trial Protocol title and version number), the documents reviewed (Clinical Trial Protocol, Informed Consent Form, Investigator's Brochure, Investigator's CV, etc.) and the date of the review should be clearly stated on the written (IRB/IEC) approval/favorable opinion.

Investigational Product will not be released at the study site and the Investigator will not start the study before the written and dated approval/favorable opinion is received by the Investigator and the Sponsor.

During the Clinical Trial, any amendment or modification to the Clinical Trial Protocol should be submitted to the Ethics Committee (IRB/IEC) before implementation, unless the change is necessary to eliminate an immediate hazard to the subjects, in which case the IRB/IEC should be informed as soon as possible. It

-

All documentation of **MaaT Pharma** is PROPRIETARY information and may not be forwarded to third parties without prior written consent from **MaaT Pharma.** Reproductions, either in part or in whole, may not be published or copied in any manner, without the explicit written consent of **MaaT Pharma**

should also be informed of any event likely to affect the safety of subjects or the continued conduct of the Clinical Trial, in particular any change in safety.
Updates to the Investigator's Brochure will be sent to the Ethics Committees and Regulatory Authorities for review and approval as required per local country regulations and guidelines (France).
A progress report is sent to the Ethics Committee as required per local country regulations and guidelines (France).

-

All documentation of **MaaT Pharma** is PROPRIETARY information and may not be forwarded to third parties without prior written consent from **MaaT Pharma.** Reproductions, either in part or in whole, may not be published or copied in any manner, without the explicit written consent of **MaaT Pharma**

16 STUDY CONDUCT CONSIDERATIONS

16.1 RESPONSIBILITIES OF THE INVESTIGATOR(S)

The Investigator(s) undertake(s) to perform the Clinical Trial in accordance with this Clinical Trial Protocol, ICH guidelines for Good Clinical Practice and the applicable regulatory requirements.

The Investigator is required to ensure compliance with all procedures required by the Clinical Trial Protocol and with all study procedures provided by the Sponsor (including security rules). The Investigator agrees to provide reliable data and all information requested by the Clinical Trial Protocol (with the help of the CRF, Discrepancy Resolution Form (DRF) or other appropriate instrument) in an accurate and legible manner according to the instructions provided and to ensure direct access to source documents by Sponsor representatives.

If any circuit includes transfer of data particular attention should be paid to the confidentiality of the subject's data to be transferred.

The Investigator may appoint such other individuals as he/she may deem appropriate as Sub-Investigators to assist in the conduct of the Clinical Trial in accordance with the Clinical Trial Protocol. All Sub-Investigators shall be appointed and listed in a timely manner. The Sub- Investigators will be supervised by and work under the responsibility of the Investigator. The Investigator will provide them with a copy of the Clinical Trial Protocol and all necessary information.

16.2 RESPONSIBILITIES OF THE SPONSOR

The Sponsor of this Clinical Trial is responsible to Health Authorities for taking all reasonable steps to ensure the proper conduct of the Clinical Trial Protocol as regards ethics, Clinical Trial Protocol compliance, and integrity and validity of the data recorded on the Case Report Forms. Thus, the main duty of the Monitoring Team is to help the Investigator and the Sponsor maintain a high level of ethical, scientific, technical and regulatory quality in all aspects of the Clinical Trial.

At regular intervals during the Clinical Trial, the site will be contacted, through monitoring visits, letters or telephone calls, by a representative of the Monitoring Team to review study progress, Investigator and subject compliance with Clinical Trial Protocol requirements and any emergent problems. These monitoring visits, will include but not be limited to review of the following aspects: subject informed consent, subject recruitment and follow-up, Serious Adverse Event documentation and reporting, AE documentation, Investigational Product allocation, subject compliance with the Investigational Product regimen, Investigational Product accountability, concomitant therapy use and quality of data.

16.3 SOURCE DOCUMENT REQUIREMENTS

According to the ICH guidelines for Good Clinical Practice, the Monitoring Team must check the Case Report Form entries against the source documents, except for the pre-identified source data directly recorded in the Case Report Form. The Informed Consent Form will include a statement by which the subject allows the Sponsor’s duly authorized personnel and the regulatory authorities to have direct access to original medical records which support the data on the Case Report Forms (e.g., subject's medical file, appointment books, original laboratory records, etc.). These personnel, bound by professional secrecy, must maintain the confidentiality of all personal identity or personal medical information (according to confidentiality rules).

Subject's identification details, medical history, associated diseases, data related to the studied pathology, previous and concomitant medications, a statement that the informed consent was signed by the subject,

-

All documentation of **MaaT Pharma** is PROPRIETARY information and may not be forwarded to third parties without prior written consent from **MaaT Pharma.** Reproductions, either in part or in whole, may not be published or copied in any manner, without the explicit written consent of **MaaT Pharma**

study identification (name), dates of study visits, treatment number, dates of administration of IP, examinations and assessments carried out at each visit, adverse events (+ follow-up), date of study discontinuation (if any) and reason.

Source documentation must include the faxed Central lab reports (dated and signed by the Principal Investigator or Sub-Investigator), the screening, screen failure, discontinuation, and end of study).

In case of SAE, the site should file in the source document at least copies of the hospitalization reports and any relevant examination reports documenting the follow-up of the SAE.

NOTE: Patient's involvement in the study should be clearly documented in his/her medical file; details should include the study protocol number, the hospital/unit code, the patient's identification and enrollment number, the patient's consent/assent to take part in the study (with the date of consent/assent), the dates of all study visits. Such essential documents must be retained by the Investigator in accordance with local regulations.

-

All documentation of **MaaT Pharma** is PROPRIETARY information and may not be forwarded to third parties without prior written consent from **MaaT Pharma.** Reproductions, either in part or in whole, may not be published or copied in any manner, without the explicit written consent of **MaaT Pharma**

17 ADMINISTRATIVE RULES

17.1 CURRICULUM VITAE

A current copy of the curriculum vitae describing the experience, qualification and training of each Investigator and Co-Investigator will be provided to the Sponsor prior to the beginning of the Clinical Trial.

17.2 RECORD RETENTION IN STUDY SITES (S)

The Investigator must maintain confidential all study documentation and take measures to prevent accidental or premature destruction of these documents.

It is recommended that the Investigator retain the study documents at least twenty-five (25) years after the completion or discontinuation of the Clinical Trial.

However, applicable regulatory requirements should be considered if a longer period is required.

The Investigator must notify the Sponsor prior to destroying any study essential documents following the Clinical Trial completion or discontinuation.

If the Investigator's personal situation is such that archiving can no longer be ensured by him/her, the Investigator shall inform the Sponsor and the relevant records shall be transferred to a mutually agreed upon designee.

-

All documentation of **MaaT Pharma** is PROPRIETARY information and may not be forwarded to third parties without prior written consent from **MaaT Pharma.** Reproductions, either in part or in whole, may not be published or copied in any manner, without the explicit written consent of **MaaT Pharma**

18 CONFIDENTIALITY

To maintain patient confidentiality and to comply with applicable data protection and privacy laws and regulations, all eCRFs, study reports, and communications relating to the study will identify patients by assigned patient numbers and date of birth, if permissible by local laws and regulations. Access to patient names linked to such numbers shall be limited to the site and study doctor and shall not be disclosed to MaaT Pharma.

All information disclosed or provided by the Sponsor (or any company/institution acting on their behalf), or produced during the Clinical Trial, including, but not limited to, the Clinical Trial Protocol, the CRF, and the results obtained during the Clinical Trial, is confidential, prior to the publication of results. The Investigator and any person under his/her authority agree to undertake to keep confidential and not to disclose the information to any third party without the prior written approval of the Sponsor.

However, the submission of this Clinical Trial Protocol and other necessary documentation to the Ethics Committee (IRB/IEC) is expressly permitted, the IRB/IEC members having the same obligation of confidentiality.

The Sub-Investigators shall be bound by the same obligation as the Investigator. The Investigator shall inform the Sub-Investigators of the confidential nature of the Clinical Trial.

The Investigator and the Sub-Investigators shall use the information solely for the purposes of the Clinical Trial, to the exclusion of any use for their own or for a third party's account.

-

All documentation of **MaaT Pharma** is PROPRIETARY information and may not be forwarded to third parties without prior written consent from **MaaT Pharma.** Reproductions, either in part or in whole, may not be published or copied in any manner, without the explicit written consent of **MaaT Pharma**

19 PROPERTY RIGHTS

All information, documents and Investigational Product provided by the Sponsor or its designee are and remain the sole property of the Sponsor.

The Investigator shall not mention any information or the Product in any application for a patent or for any other intellectual property rights.

All the results, data, documents and inventions, which arise directly or indirectly from the Clinical Trial in any form, shall be the immediate and exclusive property of the Sponsor.

The Sponsor may use or exploit all the results at its own discretion, without any limitation to its property right (territory, field, continuance). The Sponsor shall be under no obligation to patent, develop, market or otherwise use the results of the Clinical Trial.

As the case may be, the Investigator and/or the Sub-Investigators shall provide all assistance required by the Sponsor, at the Sponsor's expense, for obtaining and defending any patent, including signature of legal documents.

-

All documentation of **MaaT Pharma** is PROPRIETARY information and may not be forwarded to third parties without prior written consent from **MaaT Pharma.** Reproductions, either in part or in whole, may not be published or copied in any manner, without the explicit written consent of **MaaT Pharma**

20 DATA PROTECTION

The subject's personal data and Investigator's personal data which may be included in the Sponsor database shall be treated in compliance with all applicable laws and regulations;

When archiving or processing personal data pertaining to the Investigator and/or to the subjects, the Sponsor shall take all appropriate measures to safeguard and prevent access to this data by any unauthorized third party.

All information obtained during the study (except the informed consent form data) will be input onto computer by EUROFINS OPTIMED, subcontracted by the Sponsor in conformity with the “Information Technology and Liberty Law” (Article 40 of 6 January 1978) which respects the European Regulation n°2016/679 on General Data Protection (“GDPR”) and its French application MR-001.

-

All documentation of **MaaT Pharma** is PROPRIETARY information and may not be forwarded to third parties without prior written consent from **MaaT Pharma.** Reproductions, either in part or in whole, may not be published or copied in any manner, without the explicit written consent of **MaaT Pharma**

21 SPONSOR AUDITS AND INSPECTIONS BY REGULATORY AGENCIES

For ensuring compliance with the Clinical Trial Protocol, Good Clinical Practice and applicable regulatory requirements, the Investigator should permit auditing by or on the behalf of the Sponsor and inspection by the EMA, FDA and all applicable regulatory authorities.

The Investigator agrees to allow the auditors/inspectors to have direct access to his/her study records for review, being understood that these personnel is bound by professional secrecy, and as such will not disclose any personal identity or personal medical information.

The Investigator will make every effort to help with the performance of the audits and inspections, giving access to all necessary facilities, data, and documents.

As soon as the Investigator is notified of a planned inspection by the authorities, he will inform the Sponsor and authorize the Sponsor to participate in this inspection.

The confidentiality of the data verified, and the protection of the subjects should be respected during these inspections.

Any result and information arising from the inspections by the regulatory authorities will be immediately communicated by the Investigator to the Sponsor.

The Investigator shall take appropriate measures required by the Sponsor to take corrective actions for all problems found during the audit or inspections.

-

All documentation of **MaaT Pharma** is PROPRIETARY information and may not be forwarded to third parties without prior written consent from **MaaT Pharma.** Reproductions, either in part or in whole, may not be published or copied in any manner, without the explicit written consent of **MaaT Pharma**

22 CLINICAL TRIAL RESULTS

1 The Sponsor will be responsible for preparing a Clinical Study Report and to provide a summary of study results to Investigator;

2 When the data from all investigational sites have been fully analyzed by the Sponsor, the latter will communicate the results of the Clinical Trial to the Investigator(s).

-

All documentation of **MaaT Pharma** is PROPRIETARY information and may not be forwarded to third parties without prior written consent from **MaaT Pharma.** Reproductions, either in part or in whole, may not be published or copied in any manner, without the explicit written consent of **MaaT Pharma**

23 PUBLICATIONS AND COMMUNICATIONS

The Investigator undertakes not to make any publication or release pertaining to the Study and/or results of the Study without the Sponsor's prior written consent, being understood that the Sponsor will not unreasonably withhold approval.

As the Study is being conducted at multiple sites, the Sponsor agrees that, consistent with scientific standards, first presentation or publication of the results of the Study shall be made only as part of a publication of the results obtained by all sites performing the Protocol. However, if no multicenter publication has occurred within twelve (12) months of the completion of this Study at all sites, the Investigator shall have the right to publish or present independently the results of this Study subject to the review procedure set forth herein. The Investigator shall provide the Sponsor with a copy of any such presentation or publication derived from the Study for review and comment at least thirty (30) days in advance of any presentation or submission for publication. In addition, if requested by the Sponsor, any presentation or submission for publication shall be delayed for a limited time, not to exceed ninety (90) days, to allow for filing of a patent application or such other measures as the Sponsor deems appropriate to establish and preserve proprietary rights. The Investigator shall not use the name(s) of the Sponsor and/or its employees in advertising or promotional material or publication without the prior written consent of the Sponsor. The Sponsor shall not use the name(s) of the Investigator and/or the collaborators in advertising or promotional material or publication without having received his/her and/or their prior written consent(s).

The sponsor has the right at any time to publish the results of the study.

-

All documentation of **MaaT Pharma** is PROPRIETARY information and may not be forwarded to third parties without prior written consent from **MaaT Pharma.** Reproductions, either in part or in whole, may not be published or copied in any manner, without the explicit written consent of **MaaT Pharma**

24 CLINICAL TRIAL PROTOCOL AMENDMENTS

All appendices attached hereto and referred to herein are made part of this Clinical Trial Protocol.

The Investigator should not implement any deviation from, or changes of the Clinical Trial Protocol without agreement by the Sponsor and prior review and documented approval/favorable opinion from the IRB/IEC of an amendment, except where necessary to eliminate an immediate hazard(s) to Clinical Trial Subjects, or when the change(s) involves only logistical or administrative aspects of the trial. Any change agreed upon will be recorded in writing, the written amendment will be signed by the Investigator and by the Sponsor and the signed amendment will be filed with this Clinical Trial Protocol.

Any amendment to the Clinical Trial Protocol will be submitted for approval/favorable opinion by the Ethics Committee and regulatory Authorities as required per local country regulations and guidelines.

In some instances, an amendment may require a change to the Informed Consent Form. The Investigator must receive an IRB/IEC approval/favorable opinion concerning the revised Informed Consent Form prior to implementation of the change.

All documentation of **MaaT Pharma** is PROPRIETARY information and may not be forwarded to third parties without prior written consent from **MaaT Pharma.** Reproductions, either in part or in whole, may not be published or copied in any manner, without the explicit written consent of **MaaT Pharma**

25 BIBLIOGRAPHIC REFERENCES

[1] Gu R, Wei H, Wang Y, Liu B, Zhou C, Lin D, et al. [Impact of duration of antibiotic therapy on the prognosis of patients with acute myeloid leukemia who had Gram-negative bloodstream infection in consolidation chemotherapy]. Zhonghua Xue Ye Xue Za Zhi Zhonghua Xueyexue Zazhi 2018;39:471–5. doi:10.3760/cma.j.issn.0253-2727.2018.06.006 .

[2] Hammond SP, Baden LR. Antibiotic prophylaxis during chemotherapy-induced neutropenia for patients with acute leukemia. Curr Hematol Malig R 2007;2:97–103. doi:10.1007/s11899-007-0014-2 .

[3] Aitken SL, Shelburne SA, Galloway-Peña J. 2674. Microbiome and Cumulative Antibiotic Use as Predictors of Stenotrophomonas maltophilia Infection in Patients with Acute Myeloid Leukemia Receiving Remission-Induction Chemotherapy. Open Forum Infect Dis 2019;6:S938–S938. doi:10.1093/ofid/ofz360.2352 .

[4] van den Taur 6 GNMBRR. The effects of intestinal tract bacterial diversity on mortality following allogeneic hematopoietic stem cell transplantation. BLOOD 2014;124:1174–82.

[5] Baktash A, Terveer EM, Zwittink RD, Hornung BV, Corver J, Kuijper EJ, et al. Mechanistic Insights in the Success of Fecal Microbiota Transplants for the Treatment of Clostridium difficile Infections. Front Microbiol 2018;9:1242. doi:10.3389/fmicb.2018.01242 .

[6] Cammarota G, Ianiro G, Tilg H, Rajilić-Stojanović M, Kump P, Satokari R, et al. European consensus conference on faecal microbiota transplantation in clinical practice. Gut 2017;66:569. doi:10.1136/gutjnl-2016-313017 .

[7] Wang S, Xu M, Wang W, Cao X, Piao M, Khan S, et al. Systematic Review: Adverse Events of Fecal Microbiota Transplantation. Plos One 2016;11:e0161174. doi:10.1371/journal.pone.0161174 .

[8] Kleger A, Schnell J, Essig A, Wagner M, Bommer M, Seufferlein T, et al. Fecal Transplant in Refractory Clostridium difficile Colitis. Deutsches Aerzteblatt Online 2013. doi:10.3238/arztebl.2013.0108 .

[9] Smits LP, Bouter K, de Vos WM, Borody TJ, Nieuwdorp M. Therapeutic Potential of Fecal Microbiota Transplantation. Gastroenterology 2013;145:946–53. doi:10.1053/j.gastro.2013.08.058 .

[10] Hamilton MJ, Weingarden AR, Sadowsky MJ, Khoruts A. Standardized Frozen Preparation for Transplantation of Fecal Microbiota for Recurrent Clostridium difficile Infection. Am J Gastroenterol 2012;107:761–7. doi:10.1038/ajg.2011.482 .

[11] Gough E, Shaikh H, Manges AR. Systematic Review of Intestinal Microbiota Transplantation (Fecal Bacteriotherapy) for Recurrent Clostridium difficile Infection. Clin Infect Dis 2011;53:994–1002. doi:10.1093/cid/cir632 .

[12] van Nood E, Dijkgraaf MG, Keller JJ. Duodenal infusion of feces for recurrent Clostridium difficile. New Engl J Medicine 2013;368:2145. doi:10.1056/nejmc1303919 .

[13] Davidovics ZH, Michail S, Nicholson MR, Kociolek LK, Pai N, Hansen R, et al. Fecal Microbiota Transplantation for Recurrent Clostridium difficile Infection and Other Conditions in Children. J Pediatr Gastr Nutr 2019;68:130–43. doi:10.1097/mpg.0000000000002205 .

[14] Kelly CR, Ihunnah C, Fischer M, Khoruts A, Surawicz C, Afzali A, et al. Fecal Microbiota Transplant for Treatment of Clostridium difficile Infection in Immunocompromised Patients. Am J Gastroenterol 2014;109:1065–71. doi:10.1038/ajg.2014.133 .

-

All documentation of **MaaT Pharma** is PROPRIETARY information and may not be forwarded to third parties without prior written consent from **MaaT Pharma.** Reproductions, either in part or in whole, may not be published or copied in any manner, without the explicit written consent of **MaaT Pharma**

[15] Allegretti JR, Fischer M, Sagi SV, Bohm ME, Fadda HM, Ranmal SR, et al. Fecal Microbiota Transplantation Capsules with Targeted Colonic Versus Gastric Delivery in Recurrent Clostridium difficile Infection: A Comparative Cohort Analysis of High and Lose Dose. Digest Dis Sci 2018;64:1672–8. doi:10.1007/s10620-018-5396-6 .

[16] Clancy A, Gunaratne AW, Maistry P, Kingston-Smith H, Saxena M, Borody TJ. Use of Lyophilised Microbiota Capsules for Fecal Microbiota Transplantation in Patients With Clostridium difficile Infection. Am J Gastroenterol 2018;113:S62. doi:10.14309/00000434-201810001-00114 .

[17] Dupont H, Jiang Z-D, Alexander A, Ajami N, Petrosino JF, DuPont AW, et al. Lyophilized Fecal Microbiota Transplantation Capsules for Recurrent Clostridium difficile Infection. Open Forum Infect Dis 2017;4:S381–S381. doi:10.1093/ofid/ofx163.943 .

[18] Staley C, Kaiser T, Vaughn BP, Graiziger C, Hamilton MJ, Kabage AJ, et al. Durable Long-Term Bacterial Engraftment following Encapsulated Fecal Microbiota Transplantation To Treat Clostridium difficile Infection. Mbio 2019;10. doi:10.1128/mbio.01586-19 .

[19] Iqbal U, Anwar H, Karim MA. Safety and efficacy of encapsulated fecal microbiota transplantation for recurrent Clostridium difficile infection. Eur J Gastroen Hepat 2018;30:730–4. doi:10.1097/meg.0000000000001147 .

[20] Rossen NG, MacDonald JK, de Vries EM, D'Haens GR, de Vos WM, Zoetendal EG, et al. Fecal microbiota transplantation as novel therapy in gastroenterology: A systematic review. World J Gastroentero 2015;21:5359–71. doi:10.3748/wjg.v21.i17.5359 .

[21] Mandalia A, Ward A, Tauxe W, Kraft CS, Dhere T. Fecal transplant is as effective and safe in immunocompromised as non-immunocompromised patients for Clostridium difficile. Int J Colorectal Dis 2015;31:1059–60. doi:10.1007/s00384-015-2396-2 .

[22] Kakihana K, Fujioka Y, Suda W, Najima Y, Kuwata G, Sasajima S, et al. Fecal microbiota transplantation for patients with steroid-resistant acute graft-versus-host disease of the gut. Blood 2016;128:2083–8. doi:10.1182/blood-2016-05-717652 .

[23] Webb BJ, Brunner A, Ford CD, Gazdik MA, Petersen FB, Hoda D. Fecal microbiota transplantation for recurrent Clostridium difficile infection in hematopoietic stem cell transplant recipients. Transpl Infect Dis 2016;18:628–33. doi:10.1111/tid.12550 .

[24] Shono Y, Docampo MD, Peled JU, Perobelli SM, Velardi E, Tsai JJ, et al. Increased GVHD-related mortality with broad-spectrum antibiotic use after allogeneic hematopoietic stem cell transplantation in human patients and mice. Sci Transl Med 2016;8:339ra71. doi:10.1126/scitranslmed.aaf2311 .

[25] Spindelboeck W, Schulz E, Uhl B, Kashofer K, Aigelsreiter A, Zinke-Cerwenka W, et al. Repeated fecal microbiota transplantations attenuate diarrhea and lead to sustained changes in the fecal microbiota in acute, refractory gastrointestinal graft- versus -host-disease. Haematologica 2017;102:e210–3. doi:10.3324/haematol.2016.154351 .

All documentation of **MaaT Pharma** is PROPRIETARY information and may not be forwarded to third parties without prior written consent from **MaaT Pharma.** Reproductions, either in part or in whole, may not be published or copied in any manner, without the explicit written consent of **MaaT Pharma**

26 APPENDICES

26.1 APPENDIX 1: GUT MICROBIOTA-PERTURBING ANTIBIOTICS

Intestinal microbiota treatments, such as our MaaT033 full ecosystem biotherapeutic, can be altered by antibiotics that have significant impact on the intestinal microbiota.

MaaT Pharma advises against the use of antibiotics known to have significant digestive excretion that include:

- Beta-lactam antibiotics
  - Piperacillin-tazobactam
  - Ceftazidime
  - Ceftriaxone
  - Oxacillin
  - Imipenem
  - Meropenem
  - Amoxicillin- clavulanate
  - Ampicillin-sulbactam
- Vancomycin (oral only)
- Metronidazole
- Clindamycin
- Tigecycline
- Linezolid
- Daptomycin
- Ciprofloxacin
- Levofloxacin

Some antibiotics may less disrupt the gut microbiota and thus should be considered for patient treatment:

- Beta-lactam antibiotics
  - Cefepime
- Vancomycin (IV only)
- Aztreonal
- Aminoglycosides (gentamicin, tobramycin, amikacin, IV only)
- Polymyxin B (IV)
- Trimethoprim-sulfamethoxazole
- Pentamadine (aerosolized)
- Dapsone
- Atovaquone
- Doxycycline
- Azithromycin
- Clarithromycin
- Rifampicin

Antivirals and antifungals can be given during MaaT033 treatment. Non-systemic antibiotics such as those given topically or by aerosolized route can also be administered concurrently. For systemic antibacterials not specified here, the study investigator will decide as to whether it can be administered – always with the patient's best care in mind.

-

All documentation of **MaaT Pharma** is PROPRIETARY information and may not be forwarded to third parties without prior written consent from **MaaT Pharma.** Reproductions, either in part or in whole, may not be published or copied in any manner, without the explicit written consent of **MaaT Pharma**

26.2 APPENDIX 2: BIRTH CONTROL METHODS

Based on the Clinical Trial Facilitation Group (CTFG) guidelines, methods that can achieve a failure rate of less than 1% per year when used consistently and correctly are considered as highly effective birth control methods. Such methods include:

- combined (estrogen and progestogen containing) hormonal contraception associated with inhibition of ovulation
  - oral
  - intravaginal
  - transdermal
- progestogen-only hormonal contraception associated with inhibition of ovulation
  - oral
  - injectable
  - implantable
- intrauterine device (IUD)
- intrauterine hormone-releasing system (IUS)
- bilateral tubal occlusion
- vasectomised partner
- sexual abstinence

-

All documentation of **MaaT Pharma** is PROPRIETARY information and may not be forwarded to third parties without prior written consent from **MaaT Pharma.** Reproductions, either in part or in whole, may not be published or copied in any manner, without the explicit written consent of **MaaT Pharma**
